# Terpenoids from the Soft Coral *Sinularia* sp. Collected in Yongxing Island

**DOI:** 10.3390/md16040127

**Published:** 2018-04-13

**Authors:** Guo-Fei Qin, Xu-Li Tang, Yan-Ting Sun, Xiang-Chao Luo, Jing Zhang, Leen van Ofwegen, Ping-Jyun Sung, Ping-Lin Li, Guo-Qiang Li

**Affiliations:** 1 Key Laboratory of Marine Drugs, Chinese Ministry of Education, School of Medicine and Pharmacy, Ocean University of China, Yushan Road 5, Qingdao 266003, China; qinguofei@126.com (G.-F.Q.); syt@stu.ouc.edu.cn (Y.-T.S.); luoxc981@163.com (X.-C.L.); meichuan7@163.com (J.Z.); 2 Laboratory of Marine Drugs and Biological Products, National Laboratory for Marine Science and Technology, Qingdao 266235, China; 3 College of Chemistry and Chemical Engineering, Ocean University of China, Songling Road 238, Qingdao 266100, China; tangxuli@ouc.edu.cn; 4 Nationaal Natuurhistorisch Museum, P.O. Box 9517, 2300 BA Leiden, The Netherlands; ofwegen@naturalis.nnm.nl; 5 National Museum of Marine Biology and Aquarium, Pingtung 94450, Taiwan; pjsung@nmmba.gov.tw; 6 Graduate Institute of Marine Biology, National Dong Hwa University, Pingtung 94450, Taiwan

**Keywords:** soft coral, *Sinularia* sp., sesquiterpenoid, cembranoid, antimalarial, cytotoxicities, antiviral, targets inhibitory activities

## Abstract

Three new sesquiterpenoids (sinuketal (**1**), sinulins A and B (**2** and **3**)) and two new cembranoids (sinulins C and D (**4** and **5**)), as well as eight known sesquiterpenoids (**6–13**) and eight known cembranoids (**14–21**), were isolated from the Xisha soft coral *Sinularia* sp. Their structures were elucidated by extensive spectroscopic analysis. Compound **1** possesses an unprecedented isopropyl-branched bicyclo [6.3.0] undecane carbon skeleton with unique endoperoxide moiety, and a plausible biosynthetic pathway of it was postulated. According to the reported biological properties of endoperoxide, the antimalarial, cytotoxic, antiviral, and target inhibitory activities of **1** were tested. Compound **1** showed mild in vitro antimalarial activity against *Plasmodium falciparum* 3D7, weak cytotoxic activities toward Jurkat, MDA-MB-231, and U2OS cell lines, inhibitory effects against influenza A viruses H1N1 and PR8, as well as mild target inhibitory activity against acetylcholinesterase. The other compounds were evaluated for cytotoxicities against HeLa, HCT-116, and A549 tumor cell lines and target inhibitory activities against protein tyrosine phosphatase 1B (PTP1B). Compound **20** exhibited cytotoxicities against HeLa and HCT-116, and compounds **5**, **11**, and **15** showed mild target inhibitory activities against PTP1B.

## 1. Introduction

**Figure 1:**
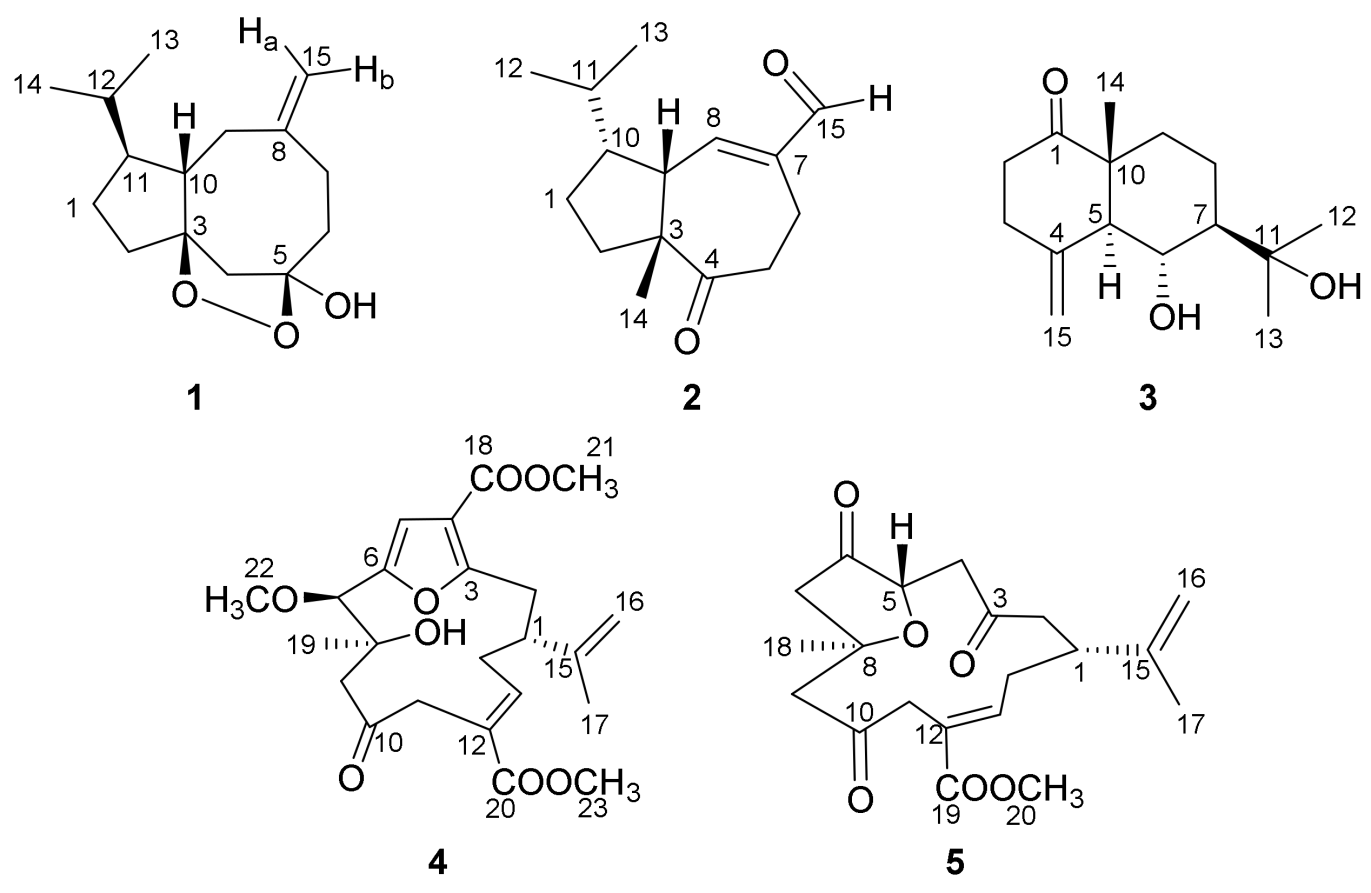
Structures of compounds **1**–**5**.

Alcyonacean octocorals have been recognized as an important source of marine secondary metabolites with a vast array of molecular architectures and diverse bioactivities [1]. As one of the most widely distributed alcyonacean corals, the genus *Sinularia* consists of almost 170 species [2], of which more than 50 have been chemically examined [3]. A wide range of bioactive metabolites—including sesquiterpenoids, diterpenoids, polyhydroxysterols, glycosides, ceramides, and cyclopentenone and butanolide derivatives—from this genus have been described [4,5]. As part of our ongoing search for bioactive compounds from soft corals of the South China Sea [6,7], we collected the soft coral *Sinularia* sp. off the coast of Yongxing Island of Xisha Islands. Herein, we report the chemical investigation of this specimen, which led to the isolation of 3 new sesquiterpenoids (sinuketal (**1**), sinulins A and B (**2** and **3**)), 2 new cembranoids (sinulins C and D (**4** and **5**)) (**1**–**5**, Figure 1), and 16 known terpenoids (**6**–**21**). Compound **1** is the first example of marine-originated isopropyl branched bicyclo [6.3.0.] undecane sesquiterpenoid, which is different from tehranolide, the only similar analogue, because of the position of the methyl and isopropyl groups [8]. As the endoperoxide moiety represents a special pharmacophore [9,10], antimalarial (against *Plasmodium falciparum* 3D7), immune-suppressive (on interleukin (IL-2) secretion), cytotoxic (toward human acute T-cell leukemia (Jurkat), human breast carcinoma (MDA-MB-231), human osteosarcoma cells (U2OS), and human lung carcinoma (A549)), antiviral (against influenza A viruses (H1N1) and (PR8)), and target inhibitory activities (on receptor tyrosine kinase c-Met and acetylcholinesterase) of compound **1** were evaluated. In addition, the other compounds were evaluated for their cytotoxicities (against human cervical epitheloid carcinoma (HeLa), human colon carcinoma (HCT-116), and human lung epithelial carcinoma (A549) tumor cell lines) and target inhibitory activities (against protein tyrosine phosphatase 1B (PTP1B)).

## 2. Results and Discussion

**Figure 2:**
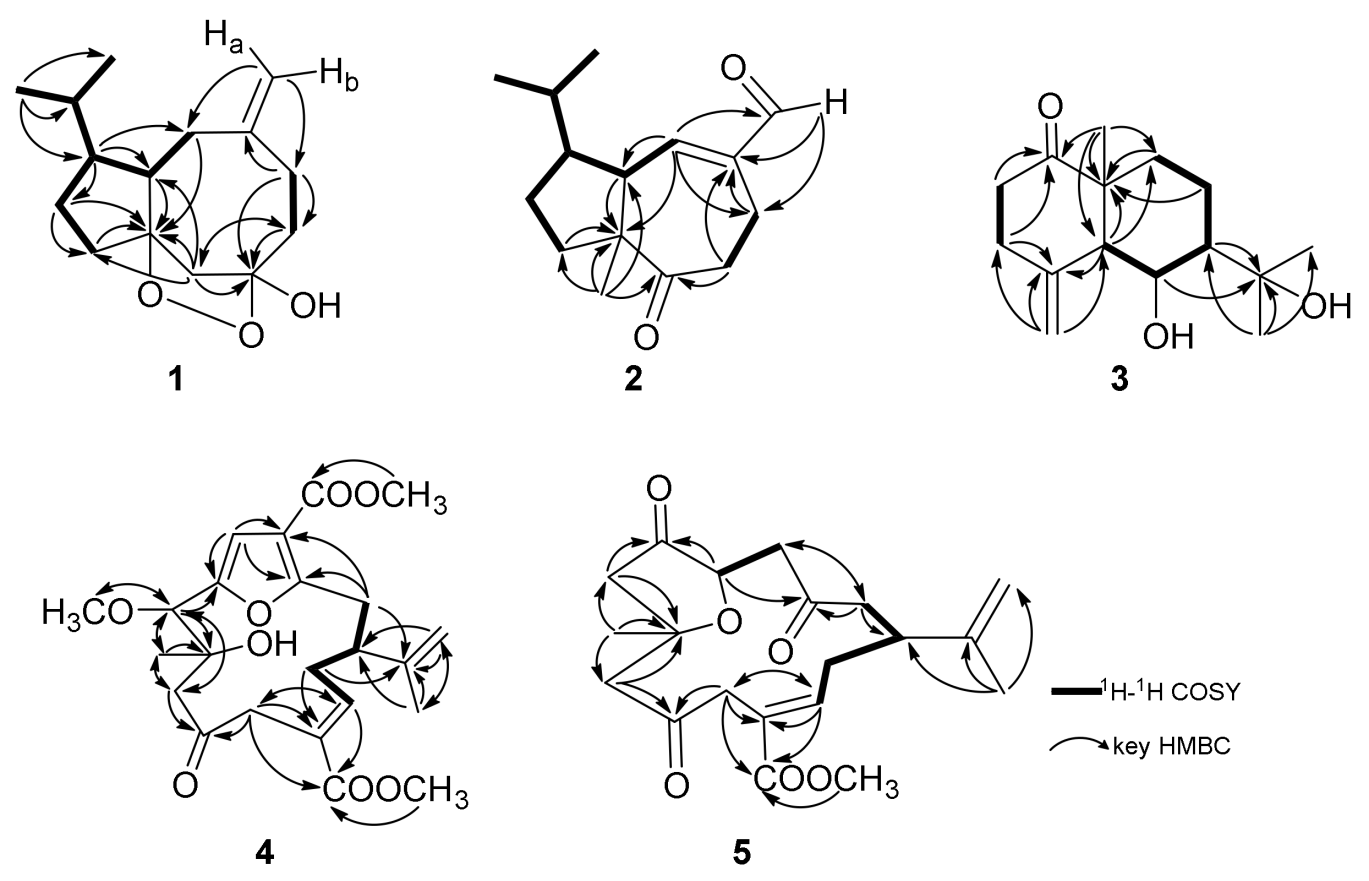
^1^H-^1^H COSY and selected key HMBC correlations of **1**–**5**.

Sinuketal (**1**) was obtained as a light-yellow oil. The molecular formula was determined as C_15_H_24_O_3_ by high-resolution electrospray ionization mass spectrometry (HRESIMS) peak at *m*/*z* 253.1800 [M + H]^+^ (calcd. 253.1798), indicating four degrees of unsaturation. The infrared radiation (IR) spectrum showed the presence of a hydroxyl group (3321 cm^−1^), which was supported by an electrospray ionization mass spectrometry (ESIMS) fragment at *m*/*z* 235 [MH − H_2_O]^+^. The ^1^H NMR spectrum of **1** (Table 1) showed the presence of two methyl doublets (*δ*_H_ 0.89 (3H, d, *J* = 6.7 Hz) and 0.95 (3H, d, *J* = 6.7 Hz)), an AX coupling system (*δ*_H_ 2.28 (1H, d, *J* = 13.4 Hz) and 3.14 (1H, d, *J* = 13.4 Hz)), and two terminal olefinic protons (*δ*_H_ 4.90 (1H, brs) and 4.93 (1H, brs)). Its ^13^C NMR and DEPT spectroscopic data (Table 1) exhibited a total of 15 carbon resonances, including 2 methyls, 6 sp^3^ methylenes, 3 sp^3^ methines, 2 sp^3^ oxygenated unprotonated carbons (including an anomeric carbon *δ*_C_ 107.4 (C)), and 1 terminal double bond (*δ*_C_ 113.0 (CH_2_) and 149.4 (C)), accounting for one degree of unsaturation. The above NMR signals suggested the structure of a tricyclic sesquiterpenoid. All the connections of H and protonated C were assigned by detailed analysis of heteronuclear multiple quantum correlation (HMQC) and heteronuclear multiple-bond correlation (HMBC) spectra. The ^1^H-^1^H correlation spectroscopy (^1^H-^1^H COSY) experiment displayed two spin systems, H_2_-6/H_2_-7 and H_2_-2/H_2_-1/H-11(H-12/H_3_-13(H_3_-14))/H-10/H_2_-9 (Figure 2). This was further supported by HMBC correlations from H_2_-7 to C-6 and from H_3_-14 to C-13, C-12 and C-11, from H-11 to C-1, C-9 and C-10, and from H_2_-1 to C-2 (Figure 2). HMBC correlations from H-1a and H_2_-2 to C-3 and from H-9*α* and H-10 to C-3 indicated the presence of a cyclopentane moiety. HMBC correlations from H_2_-15 to C-9 and C-7, from H_2_-7 to C-8, from H-6*α* and H_2_-7 to C-5, from H-6*β* to C-4, from H-4*β* to C-3, C-5 and C-10, and from H-4*α* to C-6 implied the cyclooctane moiety. Consequently, a 5/8 fused bicyclic system was established, which was further supported by HMBC correlations from H-4*β* to C-2 and from H-11 to C-9. Considering the last degree of unsaturation according to molecular formula, an AX coupling methylene, and downfield chemical shifts of oxygenated C-3 (*δ* 98.0) and anomeric C-5 (*δ* 107.4), a (C-3)–O–O–(C-5)–OH moiety should be present. Thus, the planar structure of **1** was established as shown in Figure 2.

**Figure 3:**
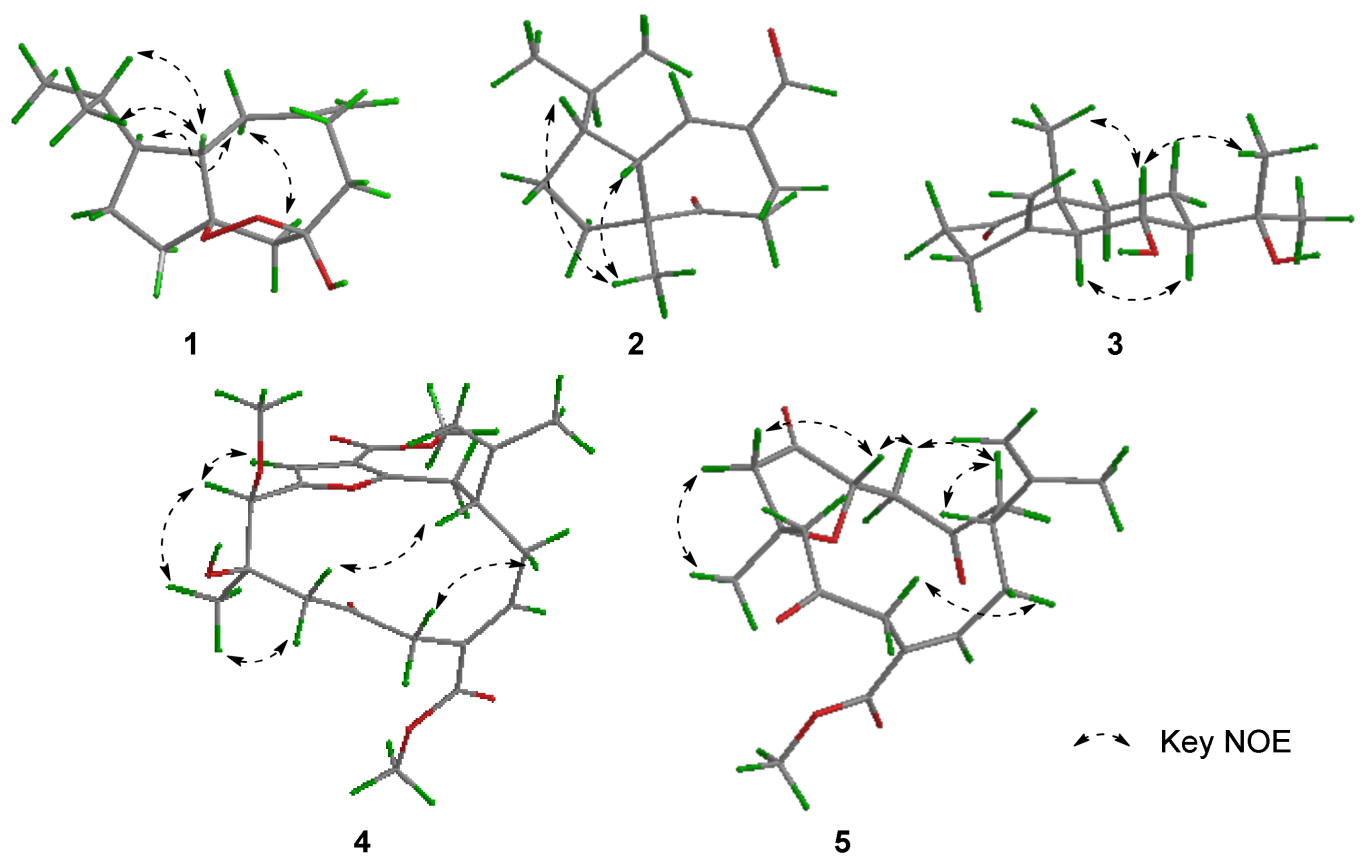
Key NOE correlations of **1**–**5**.

**Figure 4:**
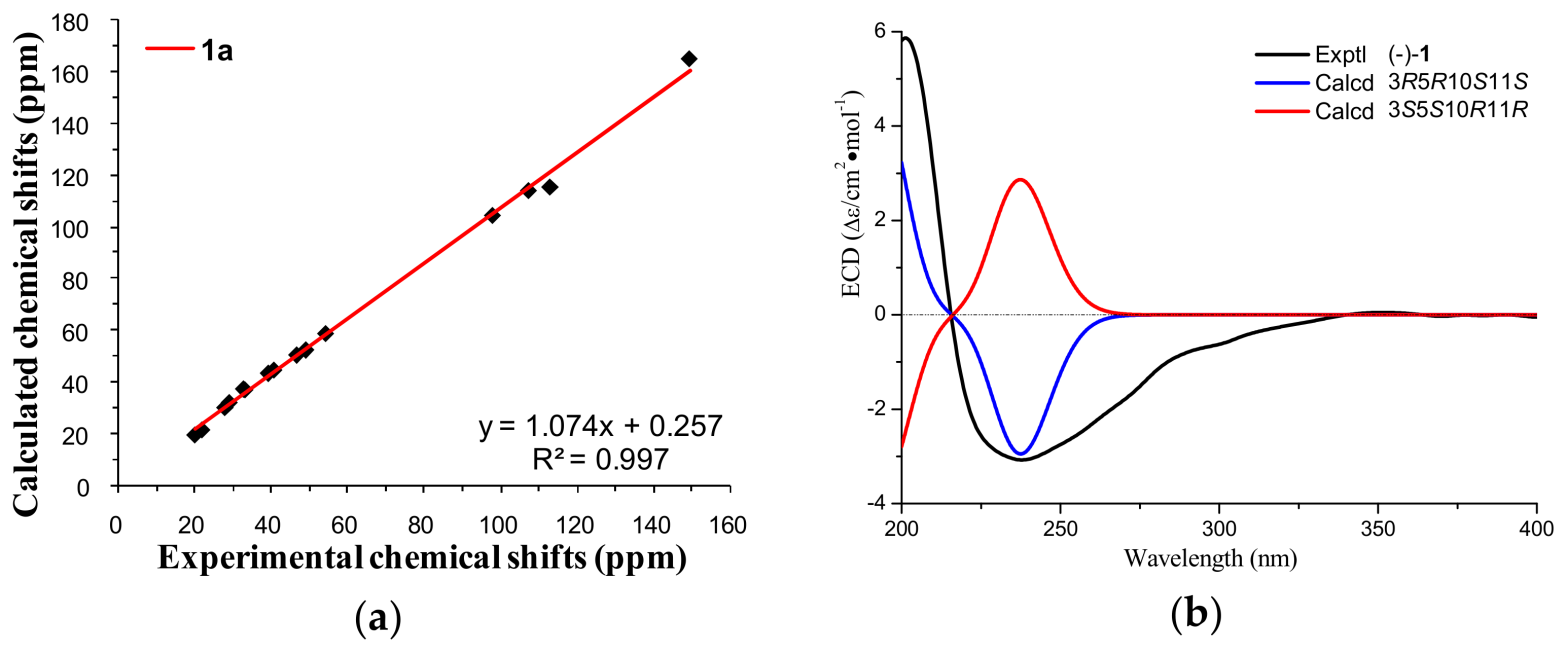
(**a**) Correlation of experimental and calculated chemical shifts of **1a**; (**b**) experimental and theoretical ECD spectra of **1**.

The relative configuration of **1** was established by nuclear Overhauser effect spectroscopy (NOESY) experiment (Figure 3) in combination with conformational analysis, and density functional theory-NMR (DFT-NMR). A *trans* configuration for H-11 and H-10 in the cyclopentane ring was deduced from the NOE correlations of H-10 with H-12 and H_3_-13, which was consistent with the absence NOE effect of H-10 with H-11. The endoperoxide group should be on the same side of the cyclooctane ring, owing to unfavorable distortions on the basis of molecular model analysis. NOE correlations of H-11 with H-9*α* and of H-9*α* with H-4*α* indicated *cis*-fused bicyclo [6.3.0] undecane. The above evidence suggested two stereochemical candidates of **1** (**1a**: 3*R*,5*R*,10*S*,11*S*; **1b**: 3*S*,5*S*,10*R*,11*R*). In order to gain further support for determination of the relative configuration of **1**, ^13^C NMR chemical shifts were calculated by the Gaussian 09 program package. The stable conformations of all the eight isomers of **1** were calculated by DFT gauge-including atomic orbitals (GIAO) model at RB3LYP/6-31+G(2d,p) level [11]. Tables S3 and S4 show the experimental and calculated ^13^C chemical shifts (relative to TMS-resonance calculated at the same level), as well as the calculated data after linear regression scaling. As a result, the calculation data of **1a** (Figure 4a) and its enantiomer **1b** gives the most reasonable correlation coefficient *R*^2^ value of 0.997, with mean absolute error 1.5 ppm and maximum absolute error 5.4 ppm (at C-15). Thus, the GIAO-based ^13^C NMR chemical shifts calculation data strongly supported the assigned carbon and relative configuration of **1**. Therefore, the relative configuration of **1** was unambiguously assigned as 3R*,5R*,10S*,11S*.

The absolute configuration of **1** was determined by time-dependent density functional theory calculation of electronic circular dichroism (TDDFT/ECD) method with the basis set RB3LYP/DGDZVP [12,13,14]. The measured ECD spectrum of **1** in methanol exhibited a negative Cotton effect (CE) at 238 nm, matching well with the calculated ECD spectra for **1a** (Figure 4b). Therefore, the absolute configuration of (−)-**1** was established as 3*R*,5*R*,10*S*,11*S*.

**Scheme 1:**
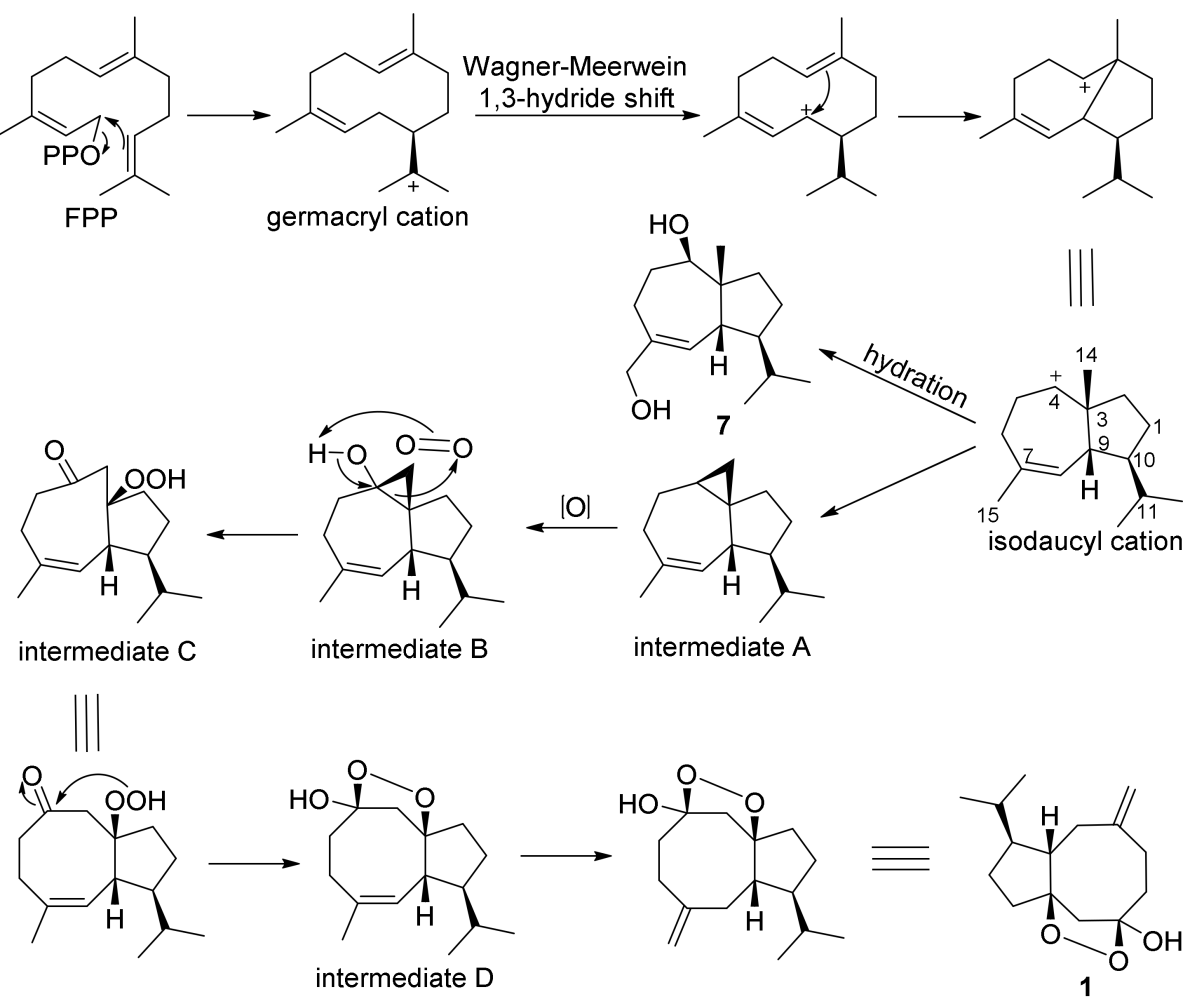
Plausible biosynthetic pathway of **1**.

Structurally, compound **1** has a previously unknown sesquiterpenoid skeleton; for this new skeleton we propose the name “sinulane”. As shown in Scheme 1, a biogenetic pathway of **1** can plausibly be retrospect to the isodaucyl cation, the biosynthetic precursor of isolated (**7**), which can be traced biogenically back to the germacryl cation and farnesyl diphosphate (FPP) [15,16]. The carbocation of the isodaucyl cation can be discharged by enzyme-catalyzed cyclization to generate intermediate A [17], rather than by quenching with a nucleophile to form compound **7**. Then, oxidation of intermediate A yields intermediate B, which subsequently undergoes oxygen-mediated hydroperoxidation [15,18] and concerted ring expansion to form hydroperoxide intermediate C. Finally, the conversion from intermediate C into **1** could be carried out by intramolecular semi-acetalization reaction [19] and Wagner–Meerwein 1,3-hydride shift.

**Figure 5:**
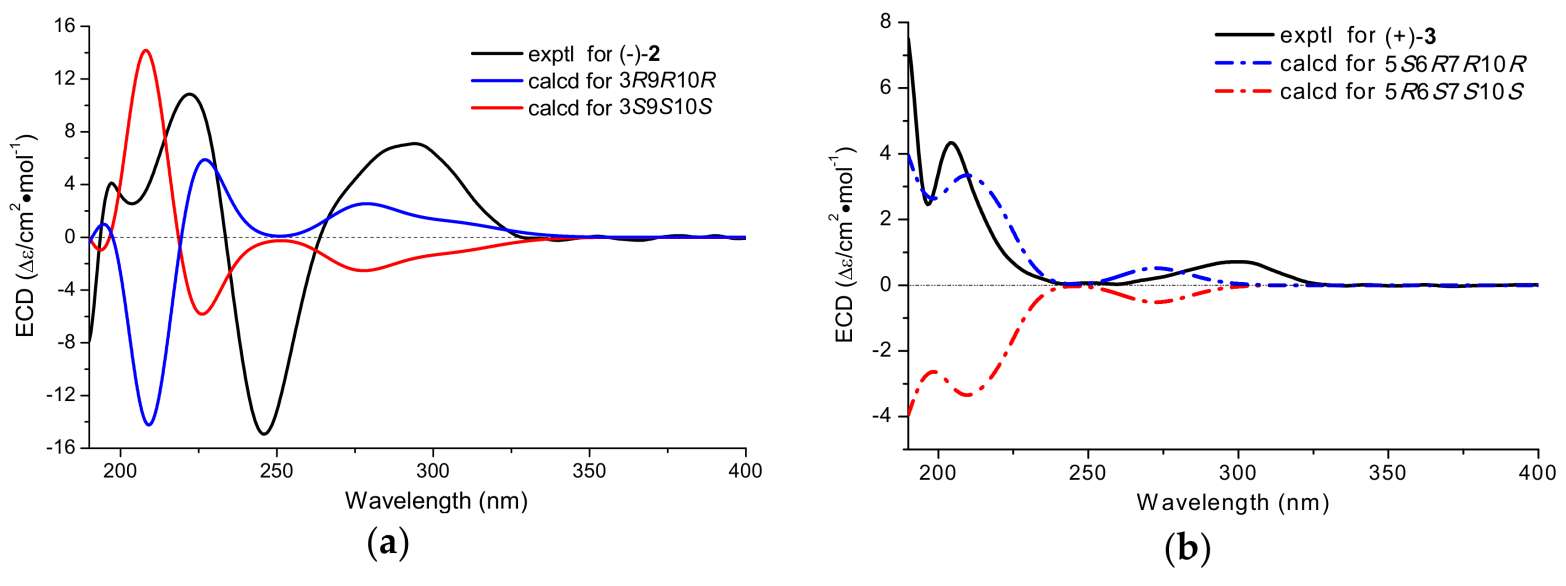
(**a**) Experimental and theoretical ECD spectra of **2**; (**b**) experimental and theoretical ECD spectra of **3**.

Sinulin A (**2**), a C-10 epimer of co-isolated 10-oxo-isodauc-3-en-15-al (**6**) [20], was obtained as a colorless oil. The molecular formula of C_15_H_22_O_2_ and five degrees of unsaturation were inferred from its HRESIMS at *m*/*z* 257.1514 [M + Na]^+^ (calcd. 257.1512). The ^13^C NMR spectra (Table 2) of **2** indicated 15 carbon signals, and a DEPT experiment exhibited the presence of three methyls, four sp^3^ methylenes, three sp^3^ methines, one sp^3^ quaternary, two sp^2^ methines, and two sp^2^ quaternary carbons. The ^13^C NMR and ^1^H NMR spectra (Table 2) implied the presence of an *α*,*β*-unsaturated conjugated aldehyde (*δ*_H_ 6.78 (1H, dd, *J* = 4.8, 1.8 Hz) and 9.49 (1H, s); *δ*_C_ 157.4 (CH), 143.7 (C) and 192.7 (CH)) and a ketone (*δ*_C_ 213.2 (C)). Thus, a bicyclic sesquiterpenoid structure of **2** was revealed. From the ^1^H-^1^H COSY spectrum (Figure 2), two partial structures of consecutive proton systems, H_2_-5/H_2_-6 and H-8/H-9/H-10/(H-11/H_3_-12(H_3_-13))/H_2_-1/H_2_-2, were established. Key HMBC correlations from H_3_-14 to C-2, C-3, C-4 and C-9, from H_2_-5 to C-4, from H-15 to C-6 and C-7, and from H-8 to C-15 assembled the planar structure of **2** (Figure 2), which has a isodaucane skeleton. The relative configuration of **2** was established from NOE experiment (Figure 3). Irradiation of H_3_-14 caused a clear NOE effect with H-9 and H-10, and no NOE effect with H-11, H_3_-12, and H_3_-13, which suggested the same orientation of H_3_-14, H-9, and H-10. The absolute configuration of **2** was deduced by TDDFT-ECD calculations. The experimental ECD curve of (−)-**2** matched well with the calculated ECD curve of 3*R*,9*R*,10*R* (Figure 5a).

Sinulin B (**3**) was isolated as a colorless, amorphous solid with molecular formula of C_15_H_24_O_3_ on the basis of its HRESIMS at *m*/*z* 253.1798 [M + H]^+^ (calcd. 253.1798), requiring four degrees of unsaturation. ^1^H and ^13^C NMR spectral data (Table 3) suggested the presence of a ketone (*δ*_C_ 213.0 (C)) and a terminal double bond (*δ*_H_ 5.28 (1H, s) and 5.05 (1H, s); *δ*_C_ 142.9 (C) and 110.6 (CH_2_)). According to the molecular formula, ^13^C NMR and DEPT spectroscopic data (Table 3), and the functionalities mentioned above, compound **3** was suggested to be a bicyclic sesquiterpenoid. The ^1^H-^1^H COSY correlations H_2_-2/H_2_-3 and H-5/H-6/H-7/H_2_-8/H_2_-9 together with the key HMBC correlations from H_3_-14 to C-1, C-5, C-9, and C-10, from H_3_-13 to C-7, C-11, and C-12, from H_2_-15 to C-3, C-4 and C-5, and from H_2_-2 and H_2_-3 to C-1 (Figure 2) established the planar structure of **3** (Figure 2), with an eudesmane skeleton. The relative configuration of **3** was determined on the basis of NOESY spectrum (Figure 3). NOE correlations of H-6 with H_3_-13 and H_3_-14 revealed that H-6, H_3_-13, and H_3_-14 were on the same face of the molecule. In addition, NOE correlation of H-7 with H-5 implied H-5 was on the opposite side of the molecule. The absolute configuration of (+)-**3** was assigned as 5*S*,6*R*,7*R*,10*R* on the basis of the experimental ECD curve of **3**, and gave good overall mirror-imagine agreement with the calculated ECD curve of 5*S*,6*R*,7*R*,10*R* (Figure 5b).

The molecular formula of sinulin C (**4**), a colorless oil, was established as C_23_H_30_O_8_ from the HRESIMS data [M + Na]^+^ at *m*/*z* 457.1836 (calcd. 457.1833). Analysis of the ^1^H and ^13^C NMR (Table 4) data indicated the presence of an isopropenyl group (*δ*_H_ 4.87 (1H, s), 4.79 (1H, s), and 1.81 (3H, s); *δ*_C_ 145.9 (C), 111.4 (CH_2_), and 20.8 (CH_3_)), a trisubstituted furane ring (*δ*_H_ 6.63 (1H, s); *δ*_C_ 161.0 (C), 149.0 (C), 114.6 (C), and 112.2 (CH)), two methyl esters (*δ*_H_ 3.79 (3H, s) and 3.72 (3H, s); *δ*_C_ 164.0 (C), 167.3 (C), 51.4 (–OCH_3_), and 52.1 (–OCH_3_)), a methoxy group (*δ*_H_ 3.26 (3H, s); *δ*_C_ 57.8 (–OCH_3_)), a ketone (*δ*_C_ 208.6 (C)), a quaternary methyl (*δ*_H_ 1.35 (3H, s); *δ*_C_ 27.9 (CH_3_)) and a trisubstituted double bond (*δ*_H_ 6.96 (1H, dd, *J* = 11.0, 4.0 Hz); *δ*_C_ 144.1 (CH) and 125.1 (C)). Their positions were confirmed by HMBC correlations from H_2_-16 and H_3_-17 to C-1, from H_2_-2 to C-3 and C-4 and from H-7 to C-6, from H_3_-21 to C-18, from H_3_-23 to C-20, from H_3_-22 to C-7, from H_2_-9 and H_2_-11 to C-10, from H_3_-19 to C-8, and from H_2_-11 to C-12 and C-13 (Figure 2), respectively. These data together with ^1^H-^1^H COSY correlations H-13/H_2_-14/H-1/H_2_-2 (Figure 2), established the planar structure of **4** as a furanocembranolide, which is same as the known compound pambanolide C [21]. Per the NOESY experiment shown in Figure 3, the NOE correlation of H-11b with H-14a indicated the 12*E* olefin. NOE correlations of H_3_-19 with H-7 and H-9*α* and of H-9*β* with H-1 indicated H_3_-19 and H-7 were on the same face of the molecule and on the opposite of H-1.

HRESIMS of sinulin D (**5**), a colorless oil, showed a pseudomolecular ion peak [M + H]^+^ at *m*/*z* 363.1801 (calcd. 363.1802), consistent with the molecular formula C_20_H_26_O_6_, and eight degrees of unsaturation. ^13^C NMR and DEPT spectrum (Table 5) showed the presence of three ketones (*δ*_C_ 207.1 (C), 212.1(C), and 205.7 (C)), an isopropenyl group (*δ*_H_ 4.95 (1H, s), 4.75 (1H, s), and 1.75 (3H, s); *δ*_C_ 144.9 (C), 112.2 (CH_2_), and 21.2 (CH_3_)), and one *α*,*β*-unsaturated methyl ester (*δ*_H_ 6.80 (1H, dd, *J* = 7.0, 7.0 Hz), and 3.71 (CH_3_, s); *δ*_C_ 140.9 (CH), 128.6 (C), 167.3 (C), and 52.0 (CH_3_)), implying bicyclic structure of **5**. The ^1^H-^1^H COSY correlations H-13/H_2_-14/H-1/H_2_-2 and H_2_-4/H-5 with the assistance of key HMBC correlations from H_3_-17 to C-1, C-15, and C-16, from H_2_-2 and H-5 to C-3, from H-5 and H_2_-7 to C-6, from H_3_-18 to C-7, C-8, and C-9, from H_2_-9 and H_2_-11 to C-10, and from H-13 to C-11, C-12, and C-19 (Figure 2) established the same norcembrene planar structure as co-isolated norcembrene 5 (**16**) [22]. The relative configuration of **5** was deduced by the NOESY experiment (Figure 3). The NOE correlation of H-11b with H_2_-14 indicated the 12*E* olefin. Key NOE correlations of H_3_-18 with H-7*α*, of H-7*β* with H-5, of H-4*β* with H-5 and H-2*β*, and of H-2*β* with H-1 suggested H-1 and H-5 were on the same face of the molecule and on the opposite of H_3_-18.

**Figure 6:**
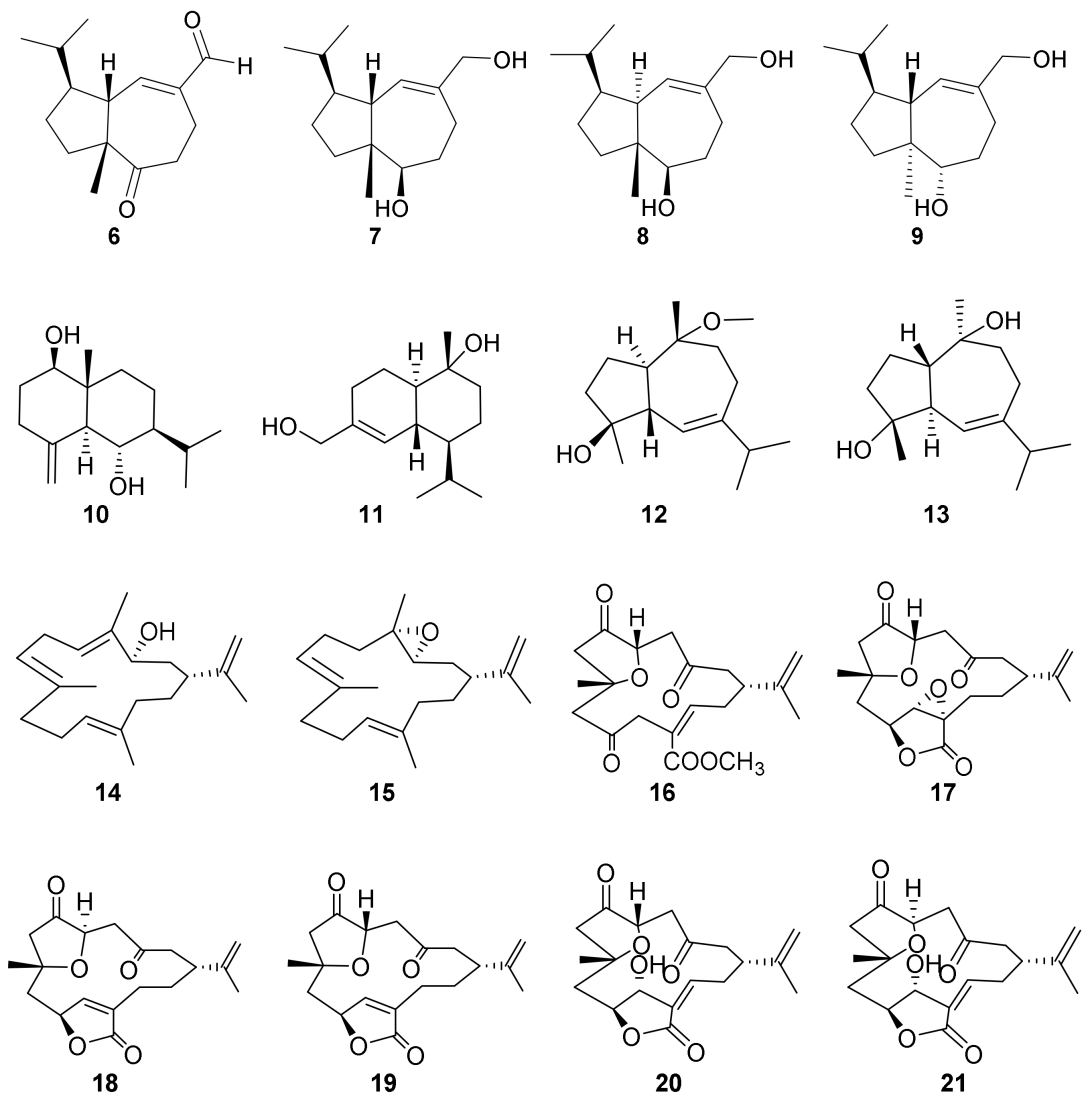
Structures of compounds **6**–**21**.

The known compounds were identified as (−)-10-oxo-isodauc-3-en-15-al (**6**) [20], (3*R*,4*R*,9*R*,10*S*)-isodauc-7-ene-4*β*,15-diol (**7**) [23], (3*R*,4*R*,9*S*,10*S*)-isodauc-7-ene-4*β*,15-diol (**8**) [24,25], (*3S*,*4S*,*9R*,*10S*)-isodauc-7-ene-4*β*,15-diol (**9**) [24,25], 1*β*,6*α*-dihydroxy-4(15)-eudesmene (**10**) [26], 15-hydroxy-*α*-cadinol (**11**) [27], 10-*O*-methyl-alismoxide (**12**) [28], guaianediol (**13**) [29], (1*R*,3*S*)-cembra-4,7,11,15-tetraen-3-ol (**14**) [30], (1*R*,3*S*,4*S*,7*E*,11*E*)-3,4-epoxycembra-7,11,15-triene (**15**) [30], norcembrene 5 (**16**) [22], norcembrenolide C (**17**) [31], sinularcasbane O (**18**) [32], scabrolide F (**19**) [32], 5-episinuleptolide (**20**) [22,33,34], and sinuleptolide (**21**) [33,34] (Figure 6). These compounds were identified by comparison of their NMR spectroscopic data with those reported in the literature.

According to the reported biological properties of endoperoxide [9,10], sinuketal (**1**) was tested for antimalarial (against *P. falciparum* 3D7), immune-suppressive (on interleukin (IL-2) secretion), cytotoxic (toward Jurkat, MDA-MB-231, U2OS, and A549 cell lines), antiviral (against influenza A viruses (H1N1) and (PR8)), and target inhibitory activities (on c-Met and acetylcholinesterase). As a result, compound **1** showed mild in vitro antimalarial activity against *P. falciparum* 3D7 (IC_50_ = 80 μM, 10 nM dihydroartemisinine as positive control), weak cytotoxic activities toward Jurkat, MDA-MB-231, and U2OS cell lines (IC_50_ = 24.9, 32.3, and 41.7 μM, respectively), inhibitory effects with IC_50_ values of 172 μM and 443 μM against influenza A viruses H1N1 and PR8, respectively (ribavirin as positive control, IC_50_ = 103 μM), and 20.2% inhibition rate against acetylcholinesterase (at 50 μg/mL, tacrine as positive control with inhibition rate 75.7% at 66 μg/L). The other compounds were evaluated for cytotoxicities against HeLa, HCT-116, and A549 tumor cell lines and target inhibitory activities against PTP1B. Compound **20** exhibited cytotoxicities against HeLa and HCT-116 with IC_50_ values of 11.6 and 33.3 μM. Compounds **5**, **11**, and **15** showed mild target inhibitory activities against PTP1B with IC_50_ values of 47.5, 22.1, and 12.5 mM (sodium orthovanadate as positive control, IC_50_ = 881 μM).

## 3. Materials and Methods

### 3.1. General Experimental Procedures

Optical rotations were measured on a Jasco P-1020 digital polarimeter (Jasco International Co. Ltd., Tokyo, Japan). UV spectra were recorded on a Beckman DU640 spectrophotometer (Beckman Instruments, Brea, CA, USA). CD spectra were obtained on a Jasco J-810 spectropolarimeter (Jasco International Co., Ltd., Tokyo, Japan). IR spectra were taken on a Nicolet NEXUS 470 FT-IR spectrophotometer (Thermo Nicolet Corporation, Madison, Wisconsin, USA) in KBr discs. NMR spectra were measured by Bruker AVANCE III 500 spectrometers (Bruker, Karlsruhe, Germany), 500 MHz for ^1^H NMR and 125 MHz for ^13^C NMR in CDCl_3_, chemical shifts *δ* in ppm referred to the solvent peaks at *δ*_H_ 7.26 and *δ*_C_ 77.0 for CDCl_3_, and coupling constant *J* in Hz. HRESIMS spectra were measured on Micromass Q-Tof Ultima GLOBAL GAA076LC mass spectrometers (Waters Asia, Ltd., Singapore) or 1290 Infinity II UHPLC/6530 Q-TOF MS (Agilent Technologies Inc., Palo Alto, CA, USA). HPLC separation was performed on a Waters 2695/2998 instrument (Waters Corporations, Milford, MA, USA) with DAD detector, equipped with a semipreparative reversed-phased column (YMC-Pack ODS-A, 250 × 10 mm, 10 μm) or an analytic reversed-phased column (ZORBAX Eclipse XDB-C18, 250 × 4.6 mm, 5 μm). Silica gel ((200–300 mesh, 300–400 mesh, and silica gel H), Qingdao Marine Chemical Inc., Qingdao, China) and Sephadex LH-20 (Amersham Pharmacia Biotech AB, Uppsala, Sweden) were used for column chromatography, and precoated silica gel plates (GF254, Qingdao Marine Chemical Inc., Qingdao, China) were used for TLC, and spots visualized by heating SiO_2_ plates sprayed with 10% H_2_SO_4_ in EtOH.

### 3.2. Animal Material

All collections of the soft coral *Sinularia* sp*.* were carried out in Yongxing Island (16°50′ N, 112°20′ E) of Xisha Islands in the South China Sea in November 2012 and were frozen immediately. The specimen was identified by Dr. Leen van Ofwegen, a co-author of this paper. The voucher specimen (No. XS-2012-04) was deposited at State Key Laboratory of Marine Drugs, Ocean University of China, China.

### 3.3. Extraction and Isolation

The frozen animals of *Sinularia* sp. (5.2 kg, wet weight; 1.4 kg, dry weight) were cut into pieces and extracted with MeOH four times (7 days each time) assisted by ultrasound at room temperature. After removal of solvent in vacuo, the combined organic extracts were desalted four times by re-dissolving with absolute methanol. The desalted residue (114 g) was subjected to vacuum liquid chromatography (VLC), eluting with a gradient of petroleum/acetone (1/0 to 1/1) and subsequently CH_2_Cl_2_/MeOH (20/1 to 0/1), to yield 10 fractions. Fraction 1 was chromatographed on silica gel eluting with a gradient of petroleum/acetone (1/0 to 0/1) to yield seven subfractions (F11–F17). Subfraction F12 was purified in a silica gel column, using petroleum/acetone (200/1), and then was isolated by semipreparative HPLC (MeOH/H_2_O, 82/18) to yield **14** (7.0 mg). Fraction 2 was applied to open octadecyl silane (ODS) column chromatography eluting by MeOH/H_2_O (70–100%) to obtain F21–F24. Subfraction F22 was refined in a silica gel column using petroleum/EtOAc (60/1), then further isolated on ODS column chromatograph using MeOH/H_2_O (40–65%) to give three fractions (F2221–F2223). F2222 was primarily purified by semipreparative HPLC (MeOH/H_2_O, 60/40) to yield a mixture of **2** and **6**, which was elucidated by NMR spectroscopic analysis, and then separated by chiral HPLC (n-hexane/isopropanol, 98/2) on chiral Daicle Chiralpack IC column (250 × 4.6 mm, 5 μm) to give **2** (2.0 mg) and **6** (2.4 mg). Subfraction F23 was purified by silica gel column using petroleum/EtOAc (80/1), and then was further separated by semipreparative HPLC (MeOH/H_2_O, 80/20) to yield **15** (3.4 mg). Fraction 3 was chromatographed on silica gel, eluting with a gradient of petroleum/acetone (50/1 to 5/1) to yield seven subfractions (F31–F37). Subfraction F33 was further separated to four fractions (F331–F334) by open-ODS column chromatography with a gradient of MeOH/H_2_O (50–100%). F332 was repurified with semipreparative HPLC (MeOH/H_2_O, 58/42) to afford **12** (27.2 mg). F333 was initially refined with semipreparative HPLC (MeOH/H_2_O, 60/40), and then further purified by Waters analytical HPLC (MeOH/H_2_O, 50/50) using an analytic reversed-phased column (4.6 × 250 mm, 5 μm) over 100 min, which yielded **1** (3.9 mg). Fraction 4 was chromatographed on a silica gel column with petroleum/acetone (30/1) to yield five subfractions (F41–F45). Subfraction F43 was purified by a silica gel column using petroleum/acetone (30/1) as eluent, and further purified by silica gel column chromatography (petroleum/acetone/EtOAc, 60/1/1), and open-ODS column chromatography (MeOH/H_2_O, 50–80%) to give five fractions (F43421–F43425). F43422 was further purified by open-ODS column chromatography (MeOH/H_2_O, 40–80%) and semipreparative HPLC (MeOH/H_2_O, 38/62) to yield **10** (2.4 mg). F43425 was further subjected to open-ODS column chromatography (MeOH/H_2_O, 70–100%) and semipreparative HPLC (MeOH/H_2_O, 60/40) to yield **4** (2.5 mg). Subfraction F44 was refined with semipreparative HPLC (MeOH/H_2_O, 60/40) to afford **16** (27.0 mg). Fraction 5 was chromatographed on silica gel eluting with petroleum/acetone (20:1), and then on silica gel eluting with CH_2_Cl_2_/MeOH (100:1) to obtain four fractions (F551–F554). F551 was applied to silica gel column eluting with petroleum/acetone (5/1) to afford F5511–F5515. F5513 was further purified by silica gel column (petroleum/acetone, 12/1), open-ODS column chromatography (MeOH/H_2_O, 30–45%), and semipreparative HPLC (MeOH/H_2_O, 35/65) to afford **5** (4.3 mg). F5514 was refined in a silica gel column using petroleum/acetone (7/1) and then further isolated on semipreparative HPLC using MeOH/H_2_O (41/59) to yield **17** (11.0 mg), **18** (9.1 mg), and **19** (4.0 mg). F552 was further subjected to open-ODS column chromatography (MeOH/H_2_O, 40%) and semipreparative HPLC (MeOH/H_2_O, 38/62) to yield **3** (1.5 mg) and **13** (33.7 mg). F553 was further purified by open-ODS column chromatography (MeOH/H_2_O, 40–80%), silica gel column (petroleum/acetone, 10/1), and semipreparative HPLC (MeOH/H_2_O, 53/47) to afford **8** (3.0 mg), **9** (3.0 mg), and **11** (9.5 mg). F554 was refined in a silica gel column using petroleum/acetone (6/1) and then further isolated on semipreparative HPLC using MeOH/H_2_O (58/42) to yield **7** (2.3 mg). Fraction 7 was separated to two fractions (F71 and F72) by Sephadex LH-20 (CH_2_Cl_2_/MeOH 1/1). Semipreparative HPLC of F72 using MeOH/H_2_O (30/70) afforded **20** (16.7 mg) and **21** (13.0 mg).

Sinuketal (**1**): light-yellow oil; ${\left[\alpha \right]}_{D}^{25}$ −46.3 (*c* 0.26, MeOH); IR (KBr) *ν*_max_ 3321, 2958, 2931, 1709 cm^−1^; ^1^H and ^13^C NMR data, see Table 1; HRESIMS: *m*/*z* 253.1800 [M + H]^+^ (calcd. for C_15_H_2__5_O_3_, 253.1798).

Sinulin A (**2**): colorless oil; ${\left[\alpha \right]}_{D}^{20}$ −28.1 (*c* 0.10, MeOH); IR (KBr) *ν*_max_ 2956, 2926, 2971, 1698, 1458, 1385, 1204 cm^−1^; ^1^H and ^13^C NMR data, see Table 2; HRESIMS: *m*/*z* 257.1514 [M + Na]^+^ (calcd. for C_15_H_2__2_O_2_Na, 257.1512).

Sinulin B (**3**): colorless amorphous solid; ${\left[\alpha \right]}_{D}^{25}$ +93.8 (*c* 0.10, MeOH); IR (KBr) *ν*_ma__x_ 3297, 2969, 2941, 1709, 1653, 1452, 1381 cm^−1^; ^1^H and ^13^C NMR data, see Table 3; HRESIMS: *m*/*z* 253.1798 [M + H]^+^ (calcd. for C_15_H_2__5_O_3_, 253.1798).

Sinulin C (**4**): colorless oil; ${\left[\alpha \right]}_{D}^{20}$ −52.0 (*c* 0.10, MeOH); IR (KBr) *ν*_ma__x_ 3447, 2932,1439, 1384, 1211, 1081 cm^−1^; ^1^H and ^13^C NMR data, see Table 4; HRESIMS: *m*/*z* 457.1836 [M + Na]^+^ (calcd. for C_23_H_30_O_8_Na, 457.1833).

Sinulin D (**5**): colorless oil; ${\left[\alpha \right]}_{D}^{25}$ +165.3 (*c* 0.10, MeOH); IR (KBr) *ν*_ma__x_ 3396, 2927, 2854, 1759, 1710, 1644, 1436, 1381, 1331, 1281, 1260, 1199, 1167, 1180 cm^−1^; ^1^H and ^13^C NMR data, see Table 5; HRESIMS: *m*/*z* 363.1801 [M + H]^+^ (calcd. for C_20_H_2__6_O_6_, 363.1802).

### 3.4. Computational Section

The quantum chemical calculations were carried out by Gaussian 09 [35] software (Gaussian Inc. Wallingford, CT, USA) using the density functional theory (DFT). The initial stereochemical structures were built with Spartan 10 software and first minimized based on molecular mechanics calculations. Then, conformational search was performed by Spartan 10 software using MMFF force filed, and conformers occurring within a 10 kcal/mol energy window from the global minimum were chosen for geometry optimization in the gas phase with the DFT method at the B3LYP/DGDZVP level. The B3LYP/DGDZVP harmonic vibrational frequencies were further calculated to confirm their stability. The stable conformations were calculated by DFT GIAO model at RB3LYP/6-31+G(2d,p) level to calculate ^13^C NMR chemical shifts. In the ECD calculations, the spin-allowed excitation energies and rotatory (*R*n) and oscillator strengths (*f*n) of the lowest excited states of stable conformers were calculated using TD-DFT method with the basis set RB3LYP/DGDZVP. Solvent effects of methanol solution were evaluated at the same DFT level by using the SCRF/PCM method in agreement with the experiment condition. Electronic transitions were expanded as Gaussian curves with a full width at half maximum (FWHM) for each peak of 0.32 eV. The ECD spectra were combined after Boltzmann weighting according to their population contribution.

### 3.5. Antimalarial Activity

The in vitro antimalarial activity was assessed against *P. falciparum* (3D7) using an SYBR-Green assay [36] with 10 nM dihydroartemisinine as positive control. Twelve diluted samples (5 µM, 10 µM, 20 µM, 40 µM, 80 µM, 100 µM, 200 µM, 400 µM, 800 µM, 1 mM, 2 mM, and 4 mM) were added to the test wells to obtain the 50% inhibiting concentration.

### 3.6. Immunosuppressive Activity

The determination of immunosuppressive activity of compound **1** on interleukin 2 (IL-2) secretion in Jurkat T-cells was performed as the literature reported [37], using DMSO and FK506 (Tacrolimus) as negative and positive controls, respectively.

### 3.7. Cytotoxic Activities

The cytotoxicities against Jurkat, MDA-MB-231, U2OS, HeLa, HCT-116, and A549 cell lines were assayed by MTT method [38] with Adriamycin as a positive control. All the cell lines were purchased from Shanghai Institute of Cell Biology (Shanghai, China).

### 3.8. Antiviral Activities

The antiviral activities against influenza A virus (H1N1) and (PR8) were evaluated by the CPE inhibition assay as the literature reported [39]. Ribavirin was used as positive control, and compounds with an inhibition rate of >70%, >50%, and <30% at 50 μg/mL were respectively regarded having strong, moderate, and weak activities.

### 3.9. Target Inhibitory Activities

Evaluation of the inhibitory effects on acetylcholinesterase was performed by the spectrophotometric method developed by Ellman with slight modifications as reported [40], and on c-Met and PTP1B by using the enzyme-linked immunosorbent assay (ELISA) methodology [41].

## 4. Conclusions

The chemical study of soft coral *Sinularia* sp. collected in Yongxing Island (China) led to the isolation of 3 new sesquiterpenoids (sinuketal (**1**), sinulins A and B (**2** and **3**)), 2 new cembranoids (sinulins C and D (**4** and **5**)), as well as 16 known terpenoids (**6**–**21**). Among them, compound **1** possesses an unprecedented isopropyl-branched bicyclo [6.3.0] undecane carbon skeleton with unique endoperoxide moiety. In bioactivity assays, compound **1** showed mild in vitro antimalarial activity against *P. falciparum* 3D7, weak cytotoxic activities toward Jurkat, MDA-MB-231, and U2OS cell lines, inhibitory effects against influenza A viruses H1N1 and PR8, as well as mild target inhibitory activity against acetylcholinesterase. Compound **20** exhibited cytotoxicities against HeLa and HCT-116, and compounds **5**, **11**, and **15** showed mild target inhibitory activities against PTP1B.

## Figures and Tables

**Table 1:** NMR spectral data of compound **1**.

No.	*δ*_H_ (*J* in Hz) ^a^	*δ*_C_ (Type) ^b^	^1^H-^1^H COSY	HMBC	NOE
1a 1b	1.72 (1H, m) 1.37 (1H, m)	28.0 (CH_2_)	H-1b, 11 H-1a, 2	C-2, 3, 10 C-2, 11	-
2	1.80 (2H, m)	33.2 (CH_2_)	H-1a	C-1, 3, 10, 11	-
3	-	98.0 (C)	-	-	-
4*α* 4*β*	3.14 (1H, d, 13.4) 2.28 (1H, d, 13.4)	49.2 (CH_2_)	H-4*β* H-4*α*	C-6, 10 C-2, 3, 5, 10	H-9*α* -
5	-	107.4 (C)	-	-	-
6*α* 6*β*	1.92 (1H, td, 13.2, 4.2) 2.06 (1H, m)	39.3 (CH_2_)	H-6*β,* 7*β* H-6*α*,7*α*	C-5, 7, 8 C-4, 5, 8	-
7*α* 7*β*	2.29 (1H, m) 2.44 (1H, td, 13.1, 2.8)	29.1 (CH_2_)	H-6*β,* 7*β* H-6*α*,7*α*	C-5, 6, 8, 9, 15 C-5, 6, 8, 15	-
8	-	149.4 (C)	-	-	-
9*α* 9*β*	2.04 (1H, m) 2.55 (1H, brd, 17.1)	40.8 (CH_2_)	H-9*β*, 10 H-9*α*, 10, 15a, 15b	C-3, 7, 8, 10, 11, 15 -	H-4*α*, 11 H-10
10	2.39 (1H, m)	46.8 (CH)	H-9*α*, 9*β*, 11	C-3, 9, 11, 12	H-9*β*, 12, 13
11	1.31 (1H, m)	54.3 (CH)	H-1a, 10, 12	C-1, 9, 10, 12, 13, 14	H-9*α*
12	1.65 (1H, m)	32.8 (CH)	H-11, 13, 14	C-1, 10, 11, 13, 14	H-10
13	0.95 (3H, d, 6.7)	22.0 (CH_3_)	H-12	C-11, 12, 14	H-10
14	0.89 (3H, d, 6.7)	20.1 (CH_3_)	H-12	C-11, 12, 13	-
15a 15b	4.90 (1H, brs) 4.93 (1H, brs)	113.0 (CH_2_)	H-9*β* H-9*β*	C-7, 9 C-7, 9	-

^a^ Spectrum recorded at 500 MHz in CDCl_3_. ^b^ Spectrum recorded at 125 MHz in CDCl_3_.

**Table 2:** NMR spectral data of compound **2**.

No.	*δ*_H_ (*J* in Hz) ^a^	*δ*_C_ (TYPE) ^b^	^1^H-^1^H COSY	HMBC	NOE
1	1.91 (1H, m) 1.53 (1H, m)	23.9 (CH_2_)	H-2, 10	C-2, 9, 10	-
2	2.24 (1H, m) 1.57 (1H, m)	33.3 (CH_2_)	H-1	C-1, 3, 14	-
3	-	60.3 (C)	-	-	-
4	-	213.2 (C)	-	-	-
5	2.56 (1H, ddd, 13.5, 7.0, 2.5) 2.43 (1H, dd, 13.5, 2.8)	38.0 (CH_2_)	H-6	C-4, 6, 7	-
6	3.00 (1H, ddd, 15.6, 7.1, 3.2) 2.05(1H, m)	20.1 (CH_2_)	H-5	C-4, 5, 7, 8, 15 -	-
7	-	143.7 (C)	-	-	-
8	6.78(1H, dd, 4.8, 1.8)	157.4 (CH)	H-9	C-3, 6, 9, 10, 15	-
9	2.48 (1H, dd, 10.4, 4.3)	49.0 (CH)	H-8, 10	C-3, 4, 7, 8, 10, 11	H-14
10	2.08 (1H, m)	49.4 (CH)	H-1, 9, 11	C-1, 8, 9, 11, 12, 13	H-14
11	1.67 (1H, m)	31.5 (CH)	H-10, 12, 13	C-1, 9, 10, 12, 13	-
12	0.88 (3H, d, 6.7)	18.9 (CH_3_)	H-11	C-10, 11, 13	-
13	0.93 (3H, d, 6.7)	21.5 (CH_3_)	H-11	C-10, 11, 12	-
14	1.02 (3H, s)	19.5 (CH_3_)	-	C-2, 3, 4, 9	H-9, 10
15	9.49 (1H, s)	192.7 (CH)	-	C-6, 7	-

^a^ Spectrum recorded at 500 MHz in CDCl_3_. ^b^ Spectrum recorded at 125 MHz in CDCl_3_.

**Table 3:** NMR spectral data of compound **3**.

No.	*δ*_H_ (*J* in Hz) ^a^	*δ*_C_ (Type) ^b^	^1^H-^1^H COSY	HMBC	NOE
1	-	213.0 (C)	-	-	-
2	2.66 (1H, m) 2.41 (1H, m)	38.3 (CH_2_)	H-3	C-1, 3	-
3	2.60 (1H, m) 2.39 (1H, m)	35.3 (CH_2_)	H-2	C-1, 2, 4, 5, 15	-
4	-	142.9 (C)	-	-	-
5	2.21 (1H, d, 9.9)	54.9 (CH)	H-6	C-1, 3, 4, 6, 9, 10, 14, 15	H-7
6	4.17 (1H, dd, 9.9, 9.9)	68.7 (CH)	H-5, 7	C-4, 5, 7, 11	H-13, 14
7	1.55 (1H, td, 9.6, 3.5)	52.7 (CH)	H-6, 8	C-6, 8, 11	H-5
8	1.73 (1H, ddd, 10.5, 6.6, 3.3) 1.14 (1H, ddd, 26.7, 13.4, 3.4)	21.9 (CH_2_)	H-7, 9	C-7, 10	-
9	1.78 (1H, dt, 13.7, 3.0) 1.62 (1H, td, 13.8, 3.6)	31.5 (CH_2_)	H-8	C-8, 10	-
10	-	49.8 (C)	-	-	-
11	-	74.6 (C)	-	-	-
12	1.26 (3H, s)	30.0 (CH_3_)	-	C-7, 11, 13	-
13	1.30 (3H, s)	24.1 (CH_3_)	-	C-7, 11, 12	H-6
14	1.01 (3H, s)	17.9 (CH_3_)	-	C-1, 5, 9, 10	H-6
15	5.28 (1H, s) 5.05 (1H, s)	110.6 (CH_2_)	H-5	C-3, 4, 5	-

^a^ Spectrum recorded at 500 MHz in CDCl_3_. ^b^ Spectrum recorded at 125 MHz in CDCl_3_.

**Table 4:** NMR spectral data of compound **4**.

No.	*δ*_H_ (*J* in Hz) ^a^	*δ*_C_ (Type) ^b^	^1^H-^1^H COSY	HMBC	NOESY
1	2.49 (1H, m)	44.1 (CH)	H-2, 14	C-2	H-9*β*
2	3.12 (1H, dd, 15.1, 3.0) 3.07 (1H, dd, 15.1, 8.6)	30.6 (CH_2_)	H-1	C-1, 3, 4, 14, 15	-
3	-	161.0 (C)	-	-	-
4	-	114.6 (C)	-	-	-
5	6.63 (1H, s)	112.2 (CH)	-	C-3, 4, 6	H-19
6	-	149.0 (C)	-	-	-
7	4.07 (1H, s)	83.7 (CH)	-	C-5, 8, 9, 19, 22	H-5, 19
8	-	73.1 (C)	-	-	-
9*α* 9*β*	2.47 (1H, d, 17.8) 3.19 (1H, d, 17.8)	46.0 (CH_2_)	-	C-7, 8, 10, 19	H-19 H-1
10	-	208.6 (C)	-	-	-
11a 11b	3.60 (1H, d, 18.2) 3.55 (1H, d, 18.1)	42.0 (CH_2_)	-	C-10, 12, 13, 20	- H-14
12	-	125.1 (C)	-	-	-
13	6.96 (1H, dd, 11.0, 4.0)	144.1 (CH)	H-14	C-1, 11, 14, 20	-
14a 14b	2.45 (1H, m) 2.28 (1H, m)	31.4 (CH_2_)	H-1, 13	C-1, 2, 12, 13, 15	H-11 -
15	-	145.9 (C)	-	-	-
16	4.87 (1H, s) 4.79 (1H, s)	111.4 (CH_2_)	H-17	C-1, 15, 17	-
17	1.81 (3H, s)	20.8 (CH_3_)	H-16	C-1, 15, 16	-
18	-	164.0 (C)	-	-	-
19	1.35 (3H, s)	27.9 (CH_3_)	-	C-7, 8, 9	H-7, 9*α*
20	-	167.3 (C)	-	-	-
21	3.79 (3H, s)	51.4 (-OCH_3_)	-	C-18	-
22	3.26 (3H, s)	57.8 (-OCH_3_)	-	C-7	-
23	3.72 (3H, s)	52.1 (-OCH_3_)	-	C-20	-

^a^ Spectrum recorded at 500 MHz in CDCl_3_. ^b^ Spectrum recorded at 125 MHz in CDCl_3_.

**Table 5:** NMR spectral data of compound **5**.

No.	*δ*_H_ (*J* in Hz) ^a^	*δ*_C_ (Type) ^b^	^1^H-^1^H COSY	HMBC	NOESY
1	2.90 (1H, m)	38.9 (CH)	H-2, 14	C-2	H-2*β*
2*α* *β*	2.70 (1H, m) 2.37 (1H, m)	44.3 (CH_2_)	H-1	C-1, 3, 4	- H-1, 4*β*
3	-	207.1 (C)	-	-	-
4*β* *α*	2.80 (1H, m)2.39 (1H, m)	46.5 (CH_2_)	H-5	C-3, 5	H-5, 2*β* -
5	4.32 (1H, brd, 11.1)	76.8 (CH)	H-4	C-3, 4, 6	H-4*β*, 7*β*
6	-	212.1 (C)	-	-	-
7*β* *α*	2.67 (1H, m) 2.43 (1H, m)	48.8 (CH_2_)	-	C-6, 8, 9, 18	H-5 H-18
8	-	79.8 (C)	-	-	-
9	2.82 (1H, m) 2.40 (1H, m)	50.7 (CH_2_)	-	C-7, 8, 10, 18	-
10	-	205.7 (C)	-	-	-
11a 11b	3.51 (1H, d, 17.6) 3.44 (1H, d, 17.7)	43.5 (CH_2_)	-	C-10, 12, 13, 19	- H-14
12	-	128.6 (C)	-	-	-
13	6.80 (1H, dd, 7.0, 7.0)	140.9 (CH)	H-14	C-1, 11, 12, 19	-
14	2.27 (2H, m)	28.8 (CH_2_)	H-1, 13	C-1, 2, 12, 13, 15	H-11b
15	-	144.9 (C)	-	-	-
16a b	4.95 (1H, s) 4.75 (1H, s)	112.2 (CH_2_)	H-17	C-1, 15, 17	-
17	1.75 (CH_3_, s)	21.2 (CH_3_)	-	C-1, 15, 16	-
18	1.41 (CH_3_, s)	27.5 (CH_3_)	-	C-7, 8, 9	H-7*β*
19	-	167.3 (C)	-	-	-
20	3.71 (CH_3_, s)	52.0 (CH_3_)	-	C-19	-

^a^ Spectrum recorded at 500 MHz in CDCl_3_. ^b^ Spectrum recorded at 125 MHz in CDCl_3_.

## Supplementary Materials

The following are available online at http://www.mdpi.com/1660-3397/16/4/127/s1, animal material information of *Sinularia* sp., computational details of **1**–**3**, and 1D and 2D NMR spectra of new compounds **1**–**5**.
