# Supplementary material for: Terpenoids from the Soft Coral Sinularia sp. Collected in Yongxing Island
Source: Mar Drugs. 2018 Apr 13;16(4):127. doi: 10.3390/md16040127 (PMC5923414; doi:10.3390/md16040127)
Supplement: Supplementary file 1 [file marinedrugs-16-00127-s001.pdf]

Supporting Information

Terpenoids from the Soft Coral *Sinularia* sp. Collected in Yongxing Island

Guo-Fei Qin, Xu-Li Tang, Yan-Ting Sun, Xiang-Chao Luo, Jing Zhang, Leen van Ofwegen,

Ping-Jyun Sung, Ping-Lin Li, and Guo-Qiang Li

Table of Contents

Experimental section		S3
1. Animal material		S3
Computational details		S3
1. Stable conformers of compound (**1-3**)		S3
2. ^{13}C NMR calculations details to support the assigned carbon and relative configuration of (**1**)		S6
3. ECD calculations details for (**1**) to determine the absolute configuration		S16
1D and 2D NMR spectra of new compounds (**1-5**)		S17
**Figure SS1**	The positive ESIMS spectrum of sinuketal (**1**)	S17
**Figure SS2**	The positive HRESIMS spectrum of sinuketal (**1**)	S18
**Figure SS3**	^{1}H NMR (500 MHz, $CDCl_3$) spectrum of sinuketal (**1**)	S19
**Figure SS4**	Amplificatory ^{1}H NMR spectrum of sinuketal (**1**)	S20
**Figure SS5**	^{13}C NMR (125 MHz, $CDCl_3$) spectrum of sinuketal (**1**)	S21
**Figure SS6**	DEPT spectrum of sinuketal (**1**)	S22
**Figure SS7**	HMQC spectrum of sinuketal (**1**)	S23
**Figure SS8**	Partial HMQC spectrum of sinuketal (**1**)	S24
**Figure SS9**	^{1}H-^{1}H COSY spectrum of sinuketal (**1**)	S25
**Figure SS10**	Partial ^{1}H-^{1}H COSY spectrum of sinuketal (**1**)	S26
**Figure SS11**	Key HMBC spectrum of sinuketal (**1**)	S27
**Figure SS12**	Partial HMBC spectrum of sinuketal (**1**) (A)	S28
**Figure SS13**	Partial HMBC spectrum of sinuketal (**1**) (B)	S29
**Figure SS14**	Partial HMBC spectrum of sinuketal (**1**) (C)	S30
**Figure SS15**	NOESY spectrum of sinuketal (**1**)	S31
**Figure SS16**	Key amplificatory NOESY spectrum of sinuketal (**1**)	S32
**Figure SS17**	The positive HRESIMS spectrum of sinulin A (**2**)	S33
**Figure SS18**	^{1}H NMR (500 MHz, $CDCl_3$) spectrum of sinulin A (**2**) and compound (**6**)	S34
**Figure SS19**	^{13}C NMR (125 MHz, $CDCl_3$) spectrum of sinulin A (**2**) and compound (**6**)	S35
**Figure SS20**	DEPT spectrum of sinulin A (**2**) and compound (**6**)	S36
**Figure SS21**	HMQC spectrum of sinulin A (**2**) and compound (**6**)	S37

**Figure SS22**	^{1}H-^{1}H COSY spectrum of sinulin A (**2**) and compound (**6**)	**S38**
**Figure SS23**	HMBC spectrum of sinulin A (**2**) and compound (**6**)	**S39**
**Figure SS24**	NOE spectrum of sinulin A (**2**)	**S40**
**Figure SS25**	The positive HRESIMS spectrum of sinulin B (**3**)	**S41**
**Figure SS26**	^{1}H NMR (500 MHz, $CDCl_3$) spectrum of sinulin B (**3**)	**S42**
**Figure SS27**	^{13}C NMR (125 MHz, $CDCl_3$) spectrum of sinulin B (**3**)	**S43**
**Figure SS28**	DEPT spectrum of sinulin B (**3**)	**S44**
**Figure SS29**	HMQC spectrum of sinulin B (**3**)	**S45**
**Figure SS30**	^{1}H-^{1}H COSY spectrum of sinulin B (**3**)	**S46**
**Figure SS31**	HMBC spectrum of sinulin B (**3**)	**S47**
**Figure SS32**	NOESY spectrum of sinulin B (**3**)	**S48**
**Figure SS33**	The positive HRESIMS spectrum of sinulin C (**4**)	**S49**
**Figure SS34**	^{1}H NMR (500 MHz, $CDCl_3$) spectrum of sinulin C (**4**)	**S50**
**Figure SS35**	^{13}C NMR (125 MHz, $CDCl_3$) spectrum of sinulin C (**4**)	**S51**
**Figure SS36**	DEPT spectrum of sinulin C (**4**)	**S52**
**Figure SS37**	HMQC spectrum of sinulin C (**4**)	**S53**
**Figure SS38**	^{1}H-^{1}H COSY spectrum of sinulin C (**4**)	**S54**
**Figure SS39**	HMBC spectrum of sinulin C (**4**)	**S55**
**Figure SS40**	NOESY spectrum of sinulin C (**4**)	**S56**
**Figure SS41**	The positive HRESIMS spectrum of sinulin D (**5**)	**S57**
**Figure SS42**	^{1}H NMR (500 MHz, $CDCl_3$) spectrum of sinulin D (**5**)	**S58**
**Figure SS43**	^{13}C NMR (125 MHz, $CDCl_3$) spectrum of sinulin D (**5**)	**S59**
**Figure SS44**	DEPT spectrum of sinulin D (**5**)	**S60**
**Figure SS45**	HMQC spectrum of sinulin D (**5**)	**S61**
**Figure SS46**	^{1}H-^{1}H COSY spectrum of sinulin D (**5**)	**S62**
**Figure SS47**	HMBC spectrum of sinulin D (**5**)	**S63**
**Figure SS48**	NOESY spectrum of sinulin D (**5**)	**S64**

Experimental section

1. Animal material.

All collections of the soft coral *Sinularia* sp. were carried out in Yongxing Island of South China Sea in November 2012 and were frozen immediately. The specimen was identified by Dr. Leen van Ofwegen (Nationaal Natuurhistorisch Museum, Leiden, The Netherlands). The voucher specimen (No. XS-2012-04) was deposited at State Key Laboratory of Marine Drugs, Ocean University of China, P. R. China.

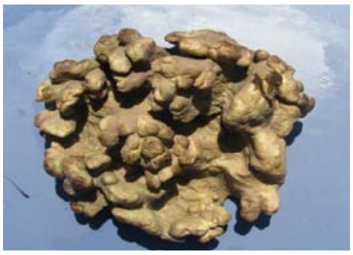

[illegible] sp.

Computational details

1. Stable conformers of compounds of (1-3)

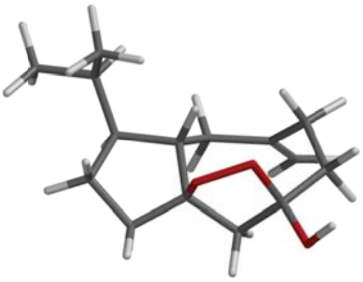

1a1 (23.9%)

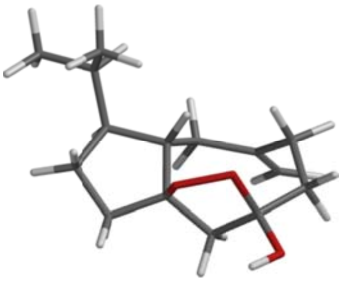

1a2 (23.7%)

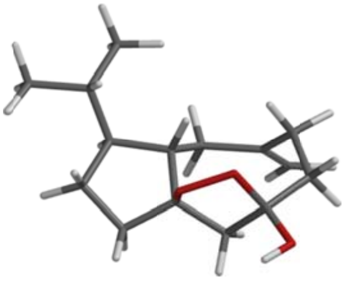

1a3 (17.5%)

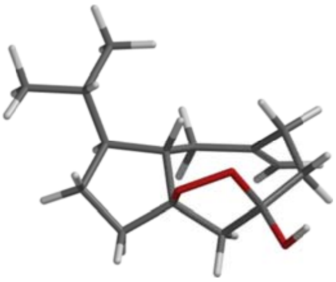

1a4 (16.6%)

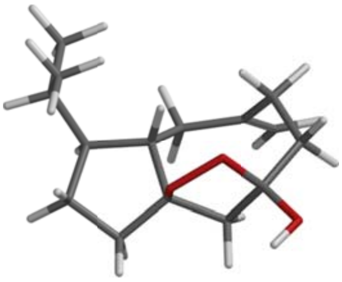

1a5 (10.2%)

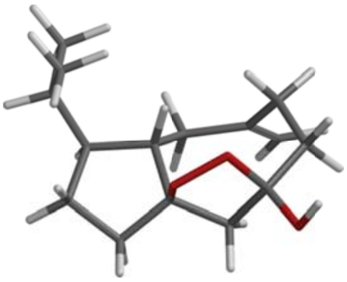

1a6 (8.1%)

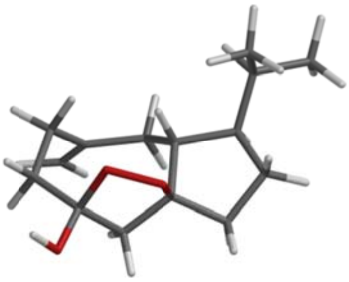

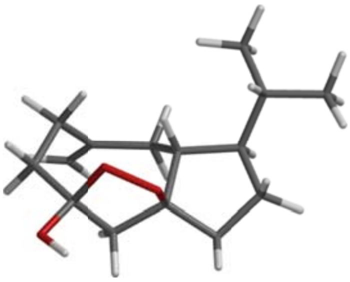

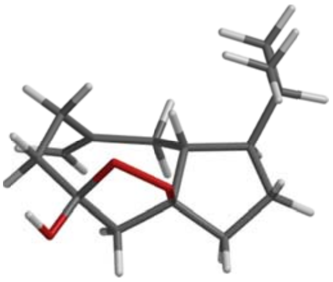

1b1 (36.5%) **1b2** (26.5%) **1b3** (12.4%)

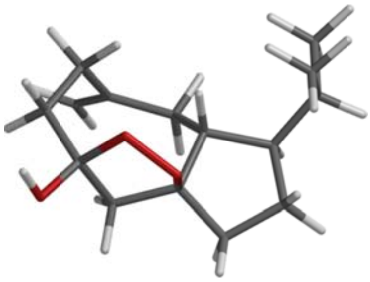

1b4 (12.4%) **1b5** (12.2%)

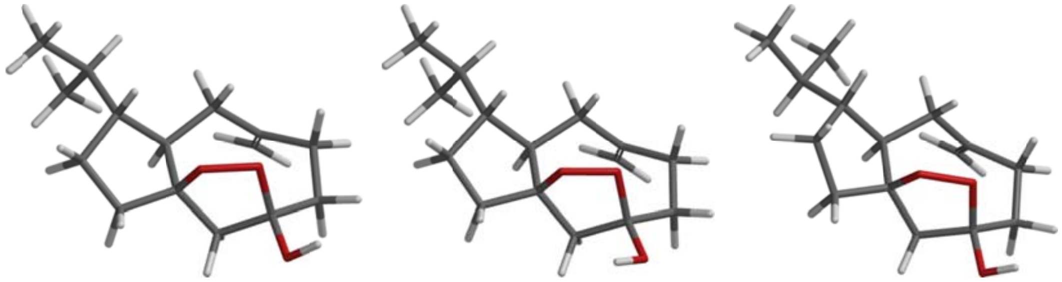

1c1 (55.0%) **1c2** (38.9%) **1c3** (3.1%)

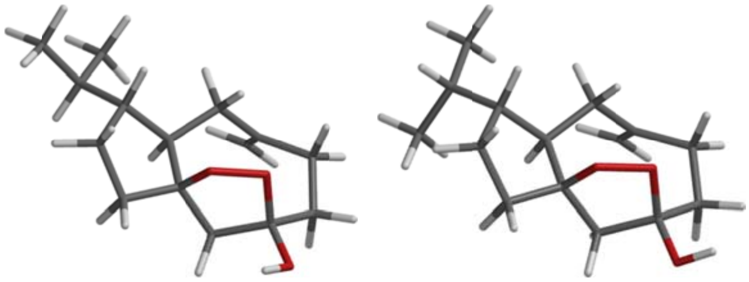

1c4 (2.1%) **1c5** (0.9%)

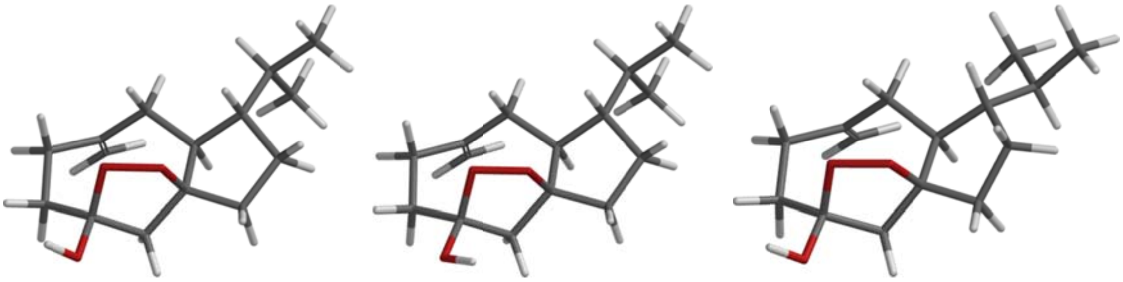

1d1 (55.0%) **1d2** (38.9%) **1d3** (3.1%)

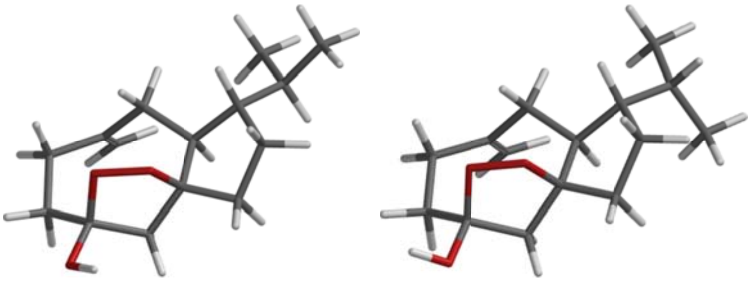

1d4 (2.1%) **1d5** (0.9%)

Figure S1. Stable conformers of compound **1** with **1a**, **1b**, **1c** and **1d** configurations, respectively.

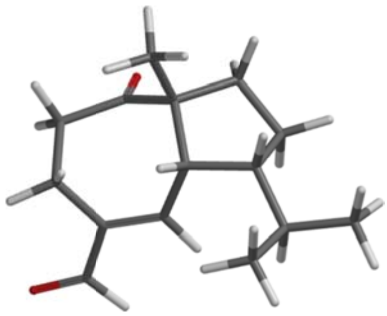

2a1 (59.5%)

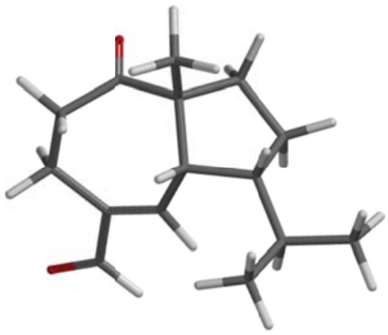

2a2 (38.8%)

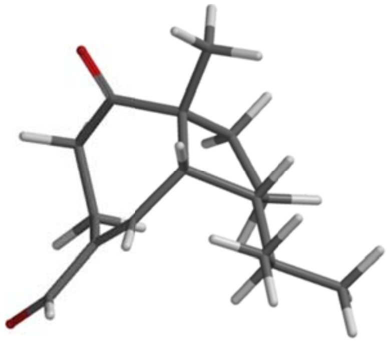

2a3 (1.6%)

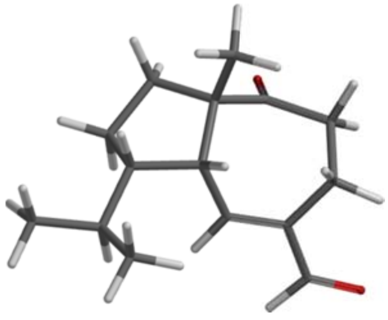

2b1 (58.8%)

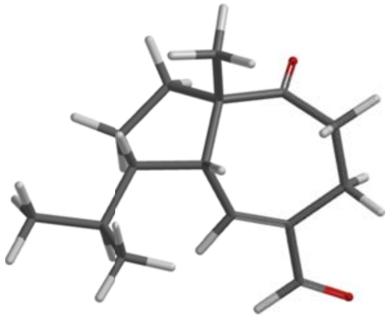

2b2 (38.5%)

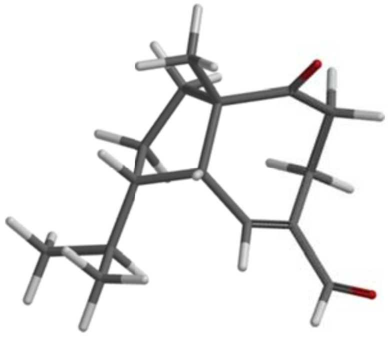

2b3 (1.6%)

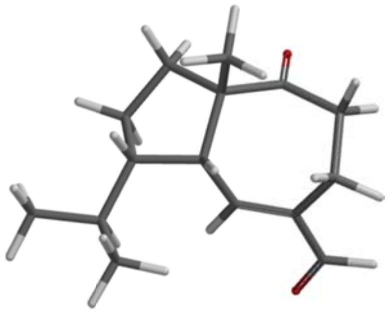

2b4 (1.1%)

Figure S2. Stable conformers of compound **2** with 3*R*9*R*10*R* (**2a**) and 3*S*9*S*10*S* (**2b**) configurations, respectively.

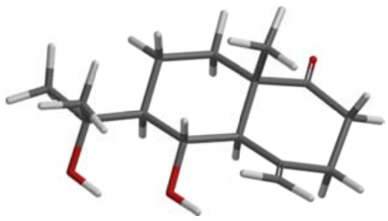

3a1 (51.1%)

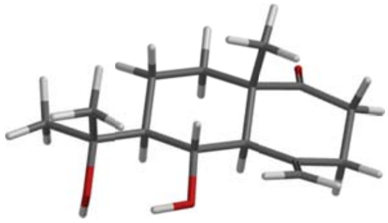

3a2 (29.2%)

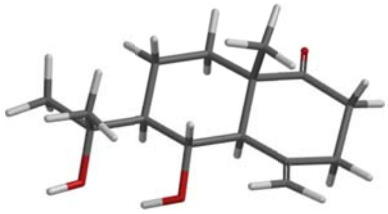

3a3 (19.7%)

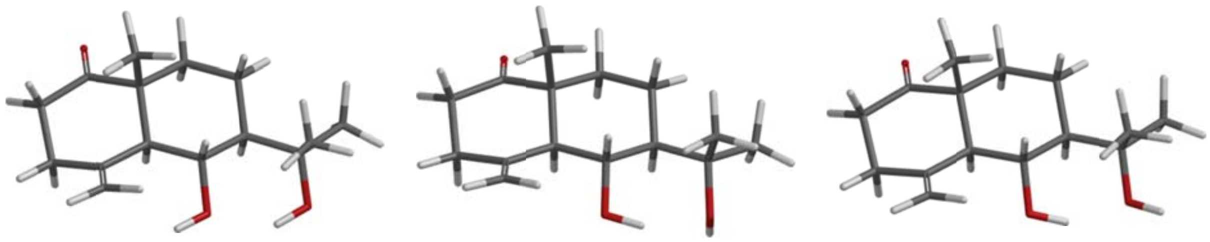

3b1 (51.1%) **3b2** (29.2%) **3b3** (19.7%)

Figure S3. Stable conformers of compound **3** with 5*R*6*S*7*S*10*S* (**3a**) and 5*S*3*R*9*R*10*R* (**3b**) configurations, respectively.

2. ^{13}C NMR calculations details to support the assigned carbon and relative configuration of 1

Table S1. Important thermodynamic parameters (a.u.) of the optimized compound **1** with **1a**, **1b**, **1c** and **1d** at B3LYP/DGDZVP level in the gas phase.

conformations	E+ZPE	G	conformations	E+ZPE	G
**1a1**	-811.279900	-811.323160	**1b1**	-811.279901	-811.323163
**1a2**	-811.279831	-811.323152	**1b2**	-811.280189	-811.322862
**1a3**	-811.280190	-811.322864	**1b3**	-811.278429	-811.322143
**1a4**	-811.280202	-811.322817	**1b4**	-811.278429	-811.322142
**1a5**	-811.278427	-811.322358	**1b5**	-811.278429	-811.322133
**1a6**	-811.278429	-811.322145			
**1c1**	-811.280859	-811.324337	**1d1**	-811.280859	-811.324337
**1c2**	-811.280504	-811.324011	**1d2**	-811.280504	-811.324011
**1c3**	-811.278638	-811.321634	**1d3**	-811.278638	-811.321634
**1c4**	-811.278212	-811.321239	**1d4**	-811.278212	-811.321239
**1c5**	-811.277544	-811.320458	**1d5**	-811.277544	-811.320458

Table S2. Optimized Z-Matrixes of compound **1** in the Gas Phase (Å) at B3LYP/6-31G(d,p) level.

**1a1**				**1a2**				**1a3**			
C	0.130217	-0.891599	-0.653646	C	0.128344	-0.875207	-0.668463	C	-0.181202	-1.028416	0.522675
C	-0.605926	0.362887	-0.083345	C	-0.603844	0.372596	-0.082932	C	0.626918	0.242758	0.110097
C	1.582973	-0.728653	-1.096476	C	1.58475	-0.71988	-1.091258	C	-1.632326	-0.852845	0.956419
C	2.361087	-0.870219	0.225693	C	2.361483	-0.874924	0.241528	C	-2.397861	-0.775145	-0.38745
C	2.975563	0.411157	0.809325	C	2.982739	0.400104	0.81795	C	-2.932552	0.6049	-0.779702
C	2.011105	1.58797	1.086312	C	2.019227	1.576976	1.090164	C	-1.905882	1.757706	-0.846589
C	1.33867	2.161693	-0.152533	C	1.349149	2.160002	-0.145994	C	-1.221625	2.096587	0.471136
C	-0.08715	1.750902	-0.509912	C	-0.08129	1.762539	-0.498839	C	0.182151	1.569753	0.759191
C	1.984735	3.054257	-0.912455	C	1.999602	3.048642	-0.90685	C	-1.832017	2.8899	1.359716
C	-2.107066	0.185599	-0.490841	C	-2.106449	0.202554	-0.48869	C	2.12743	-0.087345	0.433095
C	-2.221297	-1.258735	-1.030459	C	-2.223036	-1.230911	-1.056585	C	2.135971	-1.571199	0.878841
C	-0.844573	-1.517857	-1.65536	C	-0.845422	-1.481574	-1.683293	C	0.740098	-1.806906	1.462023

C	-3.118537	0.591478	0.609259	C	-3.113534	0.585567	0.623553	C	3.114911	0.203893	-0.721332
C	-3.132219	-0.352583	1.822937	C	-3.121268	-0.382743	1.818013	C	4.550734	-0.19945	-0.347858
C	-4.53622	0.749688	0.036947	C	-4.533529	0.754737	0.060272	C	3.091602	1.670876	-1.177078
H	-0.515982	0.28688	1.002883	H	-0.510479	0.286734	1.002097	H	0.509135	0.330416	-0.973243
O	0.277989	-1.868389	0.409436	O	0.268625	-1.870587	0.381096	O	-0.363733	-1.867602	-0.648593
O	1.381519	-1.304078	1.188271	O	1.361811	-1.318955	1.186183	O	-1.414476	-1.149975	-1.378629
O	3.352236	-1.850042	0.022746	O	3.40948	-1.80912	0.150352	O	-3.503526	-1.644374	-0.433326
H	1.870105	-1.567062	-1.735551	H	1.865057	-1.546213	-1.752481	H	-1.963016	-1.749159	1.491176
H	1.795159	0.203689	-1.618206	H	1.815656	0.214015	-1.602381	H	-1.820671	0.009148	1.594662
H	3.465667	0.140399	1.75479	H	3.472352	0.115749	1.755437	H	-3.407331	0.49314	-1.760321
H	3.76476	0.733803	0.122173	H	3.772581	0.71791	0.129192	H	-3.727041	0.85571	-0.068815
H	2.603418	2.37912	1.558803	H	2.612871	2.364305	1.56702	H	-2.451837	2.642686	-1.190709
H	1.268845	1.269232	1.823218	H	1.274443	1.259962	1.825596	H	-1.168479	1.528922	-1.621203
H	-0.763981	2.481892	-0.04185	H	-0.75158	2.491517	-0.018433	H	0.893148	2.334904	0.419638
H	-0.225872	1.882885	-1.590636	H	-0.226466	1.906351	-1.577228	H	0.320383	1.513732	1.846888
H	1.544475	3.462734	-1.818401	H	1.55891	3.463275	-1.809854	H	-1.381431	3.131553	2.318911
H	2.976079	3.415567	-0.651166	H	2.995092	3.400594	-0.648737	H	-2.803204	3.33342	1.15599
H	-2.286412	0.857638	-1.343855	H	-2.289388	0.891599	-1.327071	H	2.432659	0.526904	1.294726
H	-3.042471	-1.378794	-1.743444	H	-3.042482	-1.335031	-1.773962	H	2.935178	-1.794109	1.591187
H	-2.383528	-1.969552	-0.214425	H	-2.389289	-1.957108	-0.255126	H	2.279302	-2.2215	0.00781
H	-0.615134	-2.573214	-1.82873	H	-0.619838	-2.534751	-1.876346	H	0.450527	-2.860002	1.530012
H	-0.750423	-0.990596	-2.61306	H	-0.747603	-0.937507	-2.631096	H	0.660452	-1.371851	2.466191
H	-2.801859	1.582831	0.968265	H	-2.795367	1.569581	1.000662	H	2.799278	-0.417459	-1.573153
H	-3.720137	0.083268	2.637594	H	-3.702648	0.037905	2.645193	H	5.239567	0.004758	-1.174437
H	-2.12741	-0.553799	2.206495	H	-2.114526	-0.594086	2.190866	H	4.631019	-1.263099	-0.106508
H	-3.587823	-1.317089	1.574572	H	-3.581257	-1.34092	1.5535	H	4.902078	0.369019	0.522404
H	-5.234077	1.100826	0.804662	H	-5.228222	1.090519	0.837614	H	3.801907	1.831164	-1.995247
H	-4.558004	1.469479	-0.788837	H	-4.558698	1.49081	-0.750942	H	2.10575	1.978576	-1.53796
H	-4.919756	-0.204608	-0.342148	H	-4.918646	-0.191925	-0.336063	H	3.376804	2.343658	-0.358693
H	3.76225	-2.031372	0.880556	H	3.008657	-2.664863	-0.05996	H	-3.158392	-2.545318	-0.354014
**1a4**				**1a5**				**1a6**			
C	-0.181962	-1.038321	0.510164	C	-0.137875	-0.967705	0.675118	C	-0.142915	-0.982156	0.660364
C	0.628287	0.236769	0.108507	C	0.700971	0.269178	0.219265	C	0.703181	0.256527	0.217001
C	-1.630105	-0.859602	0.962707	C	-1.613251	-0.760125	0.999834	C	-1.614589	-0.762783	1.007045
C	-2.395261	-0.777082	-0.370069	C	-2.290144	-0.780934	-0.392509	C	-2.295018	-0.77323	-0.373254
C	-2.926378	0.607215	-0.772305	C	-2.789562	0.567443	-0.918882	C	-2.785097	0.581451	-0.90702
C	-1.900572	1.762296	-0.847664	C	-1.753019	1.710064	-1.003455	C	-1.742619	1.71993	-1.002049
C	-1.215013	2.09775	0.470178	C	-1.154997	2.145031	0.327917	C	-1.139194	2.148043	0.328986
C	0.184579	1.561406	0.762875	C	0.225578	1.641753	0.741263	C	0.234675	1.628634	0.746141
C	-1.822577	2.896803	1.355492	C	-1.818272	2.99974	1.115969	C	-1.794073	3.011634	1.114337
C	2.128002	-0.096537	0.431796	C	2.172627	-0.042349	0.664339	C	2.172399	-0.06589	0.662153
C	2.135624	-1.585447	0.860393	C	2.148401	-1.485163	1.226296	C	2.142142	-1.518651	1.196942

C	0.739472	-1.828737	1.439313	C	0.714911	-1.673206	1.731158	C	0.709419	-1.708556	1.70261
C	3.118858	0.206907	-0.716739	C	3.285489	0.192013	-0.391131	C	3.291479	0.18348	-0.383475
C	4.553974	-0.198371	-0.342589	C	3.485516	1.68231	-0.709941	C	3.510157	1.678591	-0.665542
C	3.09603	1.677687	-1.160107	C	3.107528	-0.605741	-1.693842	C	3.109877	-0.579646	-1.706369
H	0.513772	0.33061	-0.974826	H	0.643428	0.281305	-0.87092	H	0.649456	0.275963	-0.873374
O	-0.368197	-1.861831	-0.669144	O	-0.249091	-1.892054	-0.439605	O	-0.263939	-1.88848	-0.465489
O	-1.428313	-1.137102	-1.375274	O	-1.246751	-1.229699	-1.287282	O	-1.271096	-1.211368	-1.287117
O	-3.445129	-1.714223	-0.309899	O	-3.396126	-1.649155	-0.445964	O	-3.356379	-1.697035	-0.310818
H	-1.966138	-1.76291	1.477454	H	-1.981565	-1.615155	1.576105	H	-1.990842	-1.624596	1.563506
H	-1.802745	0.000812	1.607182	H	-1.837604	0.145954	1.560974	H	-1.817429	0.142935	1.576535
H	-3.401159	0.5062	-1.758064	H	-3.201169	0.384584	-1.917205	H	-3.199728	0.411977	-1.910368
H	-3.719691	0.863798	-0.062264	H	-3.626274	0.871388	-0.280894	H	-3.617168	0.894978	-0.267732
H	-2.446668	2.648066	-1.189973	H	-2.268507	2.567714	-1.448861	H	-2.253969	2.581418	-1.44513
H	-1.165332	1.530952	-1.623267	H	-0.966804	1.420754	-1.706519	H	-0.961233	1.423065	-1.706956
H	0.900389	2.326334	0.433177	H	0.959593	2.378774	0.388329	H	0.976249	2.36328	0.404388
H	0.315331	1.498248	1.85107	H	0.297164	1.668476	1.836471	H	0.298976	1.646248	1.841937
H	-1.373285	3.136592	2.315699	H	-1.429125	3.310053	2.082346	H	-1.403715	3.318145	2.08137
H	-2.790118	3.346809	1.148467	H	-2.772874	3.42656	0.819261	H	-2.742841	3.449665	0.81511
H	2.430993	0.508053	1.301144	H	2.404202	0.623848	1.508577	H	2.40212	0.583627	1.519969
H	2.934435	-1.816439	1.570685	H	2.901353	-1.633482	2.006927	H	2.897373	-1.685352	1.971729
H	2.280066	-2.225509	-0.018076	H	2.350039	-2.214997	0.4363	H	2.337169	-2.234303	0.392337
H	0.448158	-2.881847	1.492183	H	0.413372	-2.717163	1.86037	H	0.401501	-2.752319	1.813948
H	0.659519	-1.407048	2.449194	H	0.569655	-1.161144	2.690633	H	0.569804	-1.211782	2.671009
H	2.806632	-0.407161	-1.575097	H	4.214654	-0.162328	0.080579	H	4.214815	-0.192729	0.082682
H	5.245212	0.013789	-1.165212	H	4.358772	1.827773	-1.354981	H	4.388827	1.829274	-1.302081
H	4.634403	-1.263992	-0.110377	H	3.640176	2.274498	0.198858	H	3.665944	2.247301	0.257942
H	4.90208	0.362675	0.533776	H	2.621158	2.098634	-1.240092	H	2.653778	2.117197	-1.190737
H	3.80939	1.845584	-1.97415	H	3.985276	-0.475409	-2.33577	H	3.997266	-0.454865	-2.336089
H	2.111231	1.987414	-1.522144	H	2.985405	-1.67764	-1.516929	H	2.959969	-1.652087	-1.55626
H	3.377419	2.343876	-0.335028	H	2.235392	-0.267357	-2.263013	H	2.252349	-0.206568	-2.276165
H	-3.848533	-1.756077	-1.188728	H	-3.063381	-2.542212	-0.276137	H	-3.706306	-1.799672	-1.2074
**1b1**				**1b2**				**1b3**			
C	-0.130142	-0.891437	-0.653879	C	0.1812	-1.028572	0.522589	C	0.142946	-0.98218	0.660382
C	0.605844	0.363018	-0.08345	C	-0.6269	0.242721	0.110044	C	-0.703165	0.256514	0.217017
C	-1.582961	-0.728683	-1.09658	C	1.632288	-0.852938	0.956482	C	1.614597	-0.762716	1.00707
C	-2.360922	-0.870371	0.225739	C	2.397839	-0.77518	-0.387426	C	2.29504	-0.773195	-0.373207
C	-2.97536	0.410925	0.80965	C	2.932655	0.604837	-0.779641	C	2.78512	0.58149	-0.906922
C	-2.010976	1.587829	1.08654	C	1.906127	1.757749	-0.846489	C	1.742605	1.719932	-1.002037
C	-1.338776	2.161675	-0.15239	C	1.221706	2.096539	0.471161	C	1.139122	2.148133	0.32894
C	0.087014	1.751007	-0.509975	C	-0.182108	1.569677	0.7591	C	-0.2347	1.62863	0.746185
C	-1.985073	3.054187	-0.912179	C	1.832008	2.889776	1.35987	C	1.793897	3.011887	1.114198
C	2.107054	0.185901	-0.49081	C	-2.12739	-0.087326	0.4331	C	-2.172384	-0.065934	0.662137

C	2.221386	-1.258204	-1.031035	C	-2.136004	-1.571178	0.878776	C	-2.142126	-1.518688	1.196977
C	0.844631	-1.517263	-1.65586	C	-0.740158	-1.807008	1.46195	C	-0.709368	-1.708627	1.702595
C	3.118275	0.59127	0.609673	C	-3.114888	0.203973	-0.721289	C	-3.291443	0.183336	-0.383541
C	3.131953	-0.35366	1.822655	C	-4.550745	-0.199348	-0.347871	C	-3.109555	-0.579573	-1.706526
C	4.535981	0.75022	0.037655	C	-3.09159	1.670974	-1.177004	C	-3.510443	1.678431	-0.665451
H	0.515768	0.287055	1.002768	H	-0.50917	0.330394	-0.973326	H	-0.649408	0.275941	-0.873355
O	-0.27763	-1.868359	0.409071	O	0.363763	-1.867579	-0.648548	O	0.264006	-1.888489	-0.465485
O	-1.381231	-1.304263	1.188091	O	1.414275	-1.149656	-1.378688	O	1.271124	-1.211351	-1.287091
O	-3.352091	-1.850202	0.022885	O	3.503341	-1.644452	-0.43352	O	3.356391	-1.697017	-0.310789
H	-1.795379	0.203557	-1.618348	H	1.963062	-1.749214	1.491267	H	1.817335	0.143072	1.576502
H	-1.870009	-1.567282	-1.73545	H	1.82045	0.009075	1.594748	H	1.990929	-1.624406	1.563672
H	-3.764769	0.733571	0.122746	H	3.727248	0.855657	-0.068854	H	3.617115	0.895017	-0.267533
H	-3.465192	0.139982	1.75521	H	3.407347	0.493059	-1.760314	H	3.199891	0.412067	-1.910218
H	-1.268602	1.269183	1.823366	H	1.168824	1.529254	-1.621281	H	2.25393	2.581405	-1.445176
H	-2.603339	2.378905	1.559094	H	2.452323	2.642676	-1.190374	H	0.961251	1.422977	-1.706944
H	0.22565	1.883052	-1.590703	H	-0.89307	2.334872	0.419582	H	-0.976364	2.36323	0.404537
H	0.763848	2.482016	-0.041924	H	-0.320356	1.513663	1.846794	H	-0.298903	1.646164	1.841987
H	-1.544996	3.462755	-1.818172	H	1.381373	3.13131	2.319079	H	2.742624	3.449975	0.814928
H	-2.97642	3.415367	-0.650728	H	2.803211	3.333303	1.156268	H	1.40348	3.318462	2.081187
H	2.286601	0.858385	-1.343443	H	-2.432563	0.526879	1.294783	H	-2.402155	0.583604	1.519924
H	3.04249	-1.377867	-1.744158	H	-2.279379	-2.221429	0.007717	H	-2.337231	-2.234369	0.392414
H	2.383842	-1.969337	-0.215303	H	-2.935245	-1.794111	1.591092	H	-2.897312	-1.685329	1.971818
H	0.615378	-2.572576	-1.8297	H	-0.450674	-2.860143	1.529983	H	-0.569722	-1.211951	2.671037
H	0.750246	-0.989586	-2.61333	H	-0.660499	-1.371961	2.466115	H	-0.401439	-2.7524	1.813821
H	2.801233	1.582287	0.969282	H	-2.799256	-0.417347	-1.573145	H	-4.214724	-0.193139	0.082504
H	2.127096	-0.555557	2.205728	H	-5.239584	0.005321	-1.174335	H	-3.9971	-0.455289	-2.336129
H	3.587997	-1.317808	1.573697	H	-4.901965	0.368806	0.522646	H	-2.252342	-0.205884	-2.276393
H	3.71948	0.081811	2.637798	H	-4.631216	-1.263083	-0.10696	H	-2.958928	-1.651931	-1.556544
H	5.233685	1.101026	0.805667	H	-3.801942	1.831278	-1.995129	H	-4.389067	1.828983	-1.302086
H	4.919799	-0.203724	-0.342043	H	-2.105775	1.978694	-1.537975	H	-2.654096	2.117295	-1.190483
H	4.557655	1.470552	-0.78766	H	-3.376728	2.343764	-0.358598	H	-3.666485	2.246998	0.258079
H	-3.762014	-2.031652	0.880719	H	3.15831	-2.545397	-0.353848	H	3.705707	-1.80027	-1.207535
**1b4**				**1b5**				**1c1**			
C	0.142972	-0.982209	0.660344	C	0.142952	-0.982009	0.660623	C	-0.190538	-1.025399	0.396144
C	-0.703187	0.256459	0.216983	C	-0.703031	0.25664	0.217122	C	0.699994	0.226029	0.203053
C	1.614605	-0.7627	1.00707	C	1.614681	-0.762749	1.007101	C	-1.512946	-0.809392	1.136798
C	2.295088	-0.77313	-0.373174	C	2.294853	-0.773143	-0.373301	C	-2.554186	-0.668191	0.008591
C	2.785129	0.581573	-0.906867	C	2.784957	0.581533	-0.907144	C	-3.275218	0.687255	-0.090511
C	1.742571	1.71997	-1.002016	C	1.742498	1.720023	-1.002176	C	-2.449383	1.866618	-0.6534
C	1.139045	2.148152	0.328943	C	1.139301	2.148118	0.329001	C	-1.079393	2.112448	-0.041316
C	-0.234746	1.62855	0.746211	C	-0.234802	1.629181	0.745603	C	0.127022	1.386721	-0.633347
C	1.793725	3.012001	1.114173	C	1.794586	3.011115	1.114666	C	-0.927077	2.99632	0.952257

C	-2.172387	-0.066049	0.662099	C	-2.172309	-0.065648	0.662173	C	2.010912	-0.386196	-0.34963
C	-2.142088	-1.518842	1.196819	C	-2.142095	-1.518114	1.197719	C	2.195068	-1.675576	0.495773
C	-0.709351	-1.708746	1.702503	C	-0.709312	-1.708088	1.70314	C	0.777981	-2.065935	0.990543
C	-3.29147	0.183281	-0.38353	C	-3.291221	0.182661	-0.383892	C	3.241697	0.547123	-0.426978
C	-3.510591	1.678395	-0.665228	C	-3.512161	1.677538	-0.665416	C	3.636146	1.192005	0.911422
C	-3.1095	-0.579434	-1.706606	C	-3.10773	-0.579507	-1.707149	C	4.442945	-0.181553	-1.052015
H	-0.64944	0.275898	-0.873389	H	-0.64916	0.275819	-0.873221	H	0.891027	0.607409	1.217536
O	0.264094	-1.888511	-0.465529	O	0.263842	-1.888457	-0.465117	O	-0.62209	-1.586137	-0.868469
O	1.271225	-1.211338	-1.2871	O	1.270716	-1.211277	-1.287004	O	-1.813505	-0.806029	-1.209308
O	3.356483	-1.696905	-0.3107	O	3.356145	-1.696983	-0.311096	O	-3.476554	-1.720806	0.18146
H	1.990931	-1.624412	1.563648	H	1.990977	-1.624655	1.56342	H	-1.782341	-1.69053	1.723314
H	1.817337	0.143061	1.576547	H	1.81761	0.14287	1.576666	H	-1.483136	0.060619	1.794152
H	3.617085	0.895146	-0.267448	H	3.617105	0.895037	-0.267918	H	-3.654585	0.924615	0.909107
H	3.199938	0.412175	-1.910152	H	3.199592	0.411969	-1.910459	H	-4.157076	0.558436	-0.731666
H	0.961245	1.422987	-1.706942	H	0.960973	1.423034	-1.706855	H	-2.327644	1.71311	-1.730555
H	2.253873	2.58146	-1.445152	H	2.253773	2.581458	-1.445451	H	-3.057499	2.769664	-0.529139
H	-0.298884	1.645998	1.84202	H	-0.300034	1.647861	1.841312	H	-0.113282	1.014688	-1.633091
H	-0.976447	2.363172	0.404688	H	-0.976168	2.363417	0.402412	H	0.928926	2.124297	-0.754517
H	1.403274	3.318578	2.081147	H	1.404284	3.317477	2.081766	H	0.047536	3.213783	1.382607
H	2.742404	3.450191	0.814897	H	2.743581	3.448731	0.815592	H	-1.770656	3.539791	1.369849
H	-2.402148	0.583405	1.519954	H	-2.402349	0.584325	1.519567	H	1.787314	-0.7001	-1.378634
H	-2.33711	-2.234477	0.392194	H	-2.337556	-2.234126	0.39351	H	2.656734	-2.475433	-0.089181
H	-2.897321	-1.685569	1.971601	H	-2.897175	-1.684291	1.972764	H	2.854545	-1.490144	1.349911
H	-0.569755	-1.21214	2.670979	H	-0.569454	-1.211087	2.671389	H	0.71595	-2.027704	2.082492
H	-0.401373	-2.752517	1.813643	H	-0.401454	-2.751833	1.814662	H	0.469205	-3.069085	0.684056
H	-4.214711	-0.193348	0.082471	H	-4.214218	-0.195115	0.081678	H	2.975422	1.365022	-1.111812
H	-4.389193	1.828969	-1.301889	H	-4.390457	1.827067	-1.302756	H	4.497024	1.85499	0.774221
H	-2.654254	2.117418	-1.190146	H	-2.655999	2.117763	-1.189622	H	2.825552	1.793207	1.335371
H	-3.666735	2.246803	0.258381	H	-3.669754	2.245636	0.258141	H	3.921355	0.442881	1.658525
H	-3.997115	-0.455297	-2.336144	H	-3.996395	-0.458854	-2.335864	H	5.277857	0.508183	-1.215609
H	-2.252419	-0.205465	-2.276492	H	-2.252734	-0.20205	-2.277868	H	4.180818	-0.625531	-2.018642
H	-2.958596	-1.651771	-1.55675	H	-2.952276	-1.65122	-1.557493	H	4.804579	-0.986838	-0.402814
H	3.706095	-1.799862	-1.207366	H	3.704668	-1.800812	-1.208072	H	-4.048572	-1.735217	-0.599247
**1c2**				**1c3**				**1c4**			
C	-0.189187	-1.022942	0.408078	C	-0.201907	-1.049877	0.363336	C	-0.200753	-1.049108	0.372215
C	0.698123	0.228272	0.204699	C	0.726535	0.177508	0.170366	C	0.724571	0.178509	0.170471
C	-1.516669	-0.808345	1.133647	C	-1.485117	-0.800287	1.159717	C	-1.488683	-0.802125	1.156126
C	-2.561773	-0.663966	-0.00561	C	-2.570195	-0.623563	0.078085	C	-2.579624	-0.617178	0.066051
C	-3.28397	0.686183	-0.07873	C	-3.247402	0.757021	0.007142	C	-3.253604	0.760116	0.023374
C	-2.456007	1.85722	-0.65278	C	-2.411134	1.896993	-0.61881	C	-2.416456	1.890668	-0.61554
C	-1.086038	2.110093	-0.042314	C	-1.005195	2.10428	-0.078195	C	-1.009085	2.102837	-0.079668
C	0.124411	1.388707	-0.63201	C	0.148294	1.315764	-0.699097	C	0.145955	1.315963	-0.700096

C	-0.935781	2.995563	0.950299	C	-0.778149	3.008069	0.88295	C	-0.780627	3.008183	0.879822
C	2.008626	-0.385494	-0.347678	C	2.07785	-0.418148	-0.345625	C	2.076433	-0.417481	-0.34446
C	2.196892	-1.669855	0.504544	C	2.001859	-1.946984	-0.014871	C	2.003245	-1.944788	-0.007359
C	0.782326	-2.055629	1.010398	C	0.757927	-2.125508	0.872203	C	0.763066	-2.119081	0.885706
C	3.238014	0.549035	-0.432443	C	3.35475	0.230144	0.252654	C	3.353367	0.234139	0.250461
C	3.633919	1.202101	0.901512	C	4.619107	-0.317661	-0.431723	C	4.617141	-0.313831	-0.434927
C	4.438943	-0.181623	-1.055792	C	3.380883	1.765253	0.194686	C	3.377881	1.769103	0.189412
H	0.890752	0.612316	1.217939	H	0.894317	0.579376	1.18063	H	0.89283	0.58309	1.179613
O	-0.6236	-1.597675	-0.851362	O	-0.695289	-1.574391	-0.89483	O	-0.698964	-1.585365	-0.880756
O	-1.795123	-0.795508	-1.214486	O	-1.89178	-0.781686	-1.172612	O	-1.878072	-0.771709	-1.178352
O	-3.563807	-1.649887	0.069314	O	-3.518204	-1.645199	0.292259	O	-3.613508	-1.564946	0.187286
H	-1.782487	-1.681549	1.737324	H	-1.754698	-1.674722	1.756099	H	-1.753724	-1.671081	1.76621
H	-1.497167	0.062308	1.790855	H	-1.405133	0.066905	1.81633	H	-1.417725	0.063377	1.816351
H	-3.639613	0.926287	0.928923	H	-3.559355	1.019873	1.023558	H	-3.53821	1.025439	1.047116
H	-4.172111	0.545344	-0.702497	H	-4.169136	0.655021	-0.580626	H	-4.182545	0.647976	-0.544292
H	-2.335356	1.694003	-1.728563	H	-2.348679	1.720224	-1.697389	H	-2.358143	1.704732	-1.692747
H	-3.064221	2.761045	-0.536474	H	-2.980903	2.82291	-0.48148	H	-2.985368	2.817785	-0.484293
H	-0.110877	1.019486	-1.633799	H	-0.158935	0.893207	-1.658933	H	-0.158992	0.894996	-1.66115
H	0.925586	2.128087	-0.746787	H	0.958097	2.01991	-0.909539	H	0.956221	2.020489	-0.907173
H	0.038454	3.217508	1.37943	H	0.219969	3.200046	1.268904	H	0.218276	3.202898	1.262611
H	-1.780944	3.536794	1.367593	H	-1.582707	3.595614	1.317739	H	-1.584985	3.595175	1.31571
H	1.783591	-0.705082	-1.374515	H	2.11746	-0.288113	-1.435428	H	2.114684	-0.291817	-1.434705
H	2.653853	-2.474037	-0.07806	H	1.885942	-2.531553	-0.93188	H	1.8834	-2.53348	-0.921173
H	2.861158	-1.480405	1.353933	H	2.905984	-2.307714	0.483672	H	2.909201	-2.303214	0.48931
H	0.725036	-2.004304	2.102136	H	0.991434	-1.917598	1.923045	H	0.999471	-1.903511	1.934265
H	0.476281	-3.064301	0.717707	H	0.310731	-3.121798	0.816798	H	0.320711	-3.118675	0.839972
H	2.968971	1.362333	-1.121451	H	3.39161	-0.059005	1.315257	H	3.392287	-0.052729	1.31365
H	4.493905	1.865004	0.758845	H	5.523337	0.082233	0.039576	H	5.521622	0.087927	0.034193
H	2.823586	1.805256	1.323123	H	4.678787	-1.408857	-0.389795	H	4.678342	-1.404912	-0.391092
H	3.921335	0.457834	1.652628	H	4.640334	-0.02567	-1.488822	H	4.636378	-0.023788	-1.492557
H	5.272587	0.508345	-1.224447	H	4.314306	2.144359	0.624952	H	4.311711	2.149475	0.617582
H	4.175686	-0.630804	-2.019693	H	2.557966	2.220505	0.752408	H	2.555608	2.22485	0.7476
H	4.803066	-0.983028	-0.403128	H	3.329308	2.125053	-0.839912	H	3.324516	2.126993	-0.845694
H	-3.121807	-2.509799	0.016331	H	-4.126492	-1.638017	-0.460605	H	-3.208165	-2.441385	0.115746
**1c5**				**1d1**				**1d2**			
C	-0.163128	-1.078043	0.221025	C	0.190538	-1.025399	0.396144	C	0.189187	-1.022942	0.408078
C	0.789888	0.123975	-0.014766	C	-0.699994	0.226029	0.203053	C	-0.698123	0.228272	0.204699
C	-1.352875	-0.812747	1.147019	C	1.512946	-0.809392	1.136798	C	1.516669	-0.808345	1.133647
C	-2.527786	-0.548961	0.183537	C	2.554186	-0.668191	0.008591	C	2.561773	-0.663966	-0.00561
C	-3.148605	0.858911	0.227734	C	3.275218	0.687255	-0.090511	C	3.28397	0.686183	-0.07873
C	-2.331133	1.987725	-0.441451	C	2.449383	1.866618	-0.6534	C	2.456007	1.85722	-0.65278
C	-0.871915	2.123096	-0.036502	C	1.079393	2.112448	-0.041316	C	1.086038	2.110093	-0.042314

C	0.184032	1.315138	-0.790636	C	-0.127022	1.386721	-0.633347	C	-0.124411	1.388707	-0.63201
C	-0.513425	2.982426	0.925464	C	0.927077	2.99632	0.952257	C	0.935781	2.995563	0.950299
C	2.06588	-0.501667	-0.666776	C	-2.010912	-0.386196	-0.34963	C	-2.008626	-0.385494	-0.347678
C	1.949111	-2.039932	-0.412666	C	-2.195068	-1.675576	0.495773	C	-2.196892	-1.669855	0.504544
C	0.796074	-2.212776	0.589998	C	-0.777981	-2.065935	0.990543	C	-0.782326	-2.055629	1.010398
C	3.431358	0.071112	-0.192168	C	-3.241697	0.547123	-0.426978	C	-3.238014	0.549035	-0.432443
C	3.655651	1.534941	-0.6014	C	-3.636146	1.192005	0.911422	C	-3.633919	1.202101	0.901512
C	3.701059	-0.104167	1.312059	C	-4.442945	-0.181553	-1.052015	C	-4.438943	-0.181623	-1.055792
H	1.052597	0.487988	0.987364	H	-0.891027	0.607409	1.217536	H	-0.890752	0.612316	1.217939
O	-0.796858	-1.531539	-1.001794	O	0.62209	-1.586137	-0.868469	O	0.6236	-1.597675	-0.851362
O	-1.980751	-0.684404	-1.132613	O	1.813505	-0.806029	-1.209308	O	1.795123	-0.795508	-1.214486
O	-3.494668	-1.53985	0.452116	O	3.476554	-1.720806	0.18146	O	3.563807	-1.649887	0.069314
H	-1.602023	-1.699548	1.733936	H	1.782341	-1.69053	1.723314	H	1.782487	-1.681549	1.737324
H	-1.173365	0.023046	1.82447	H	1.483136	0.060619	1.794152	H	1.497167	0.062308	1.790855
H	-3.347262	1.096212	1.27833	H	3.654585	0.924615	0.909107	H	3.639613	0.926287	0.928923
H	-4.127573	0.815159	-0.267495	H	4.157076	0.558436	-0.731666	H	4.172111	0.545344	-0.702497
H	-2.381691	1.847179	-1.525925	H	2.327644	1.71311	-1.730555	H	2.335356	1.694003	-1.728563
H	-2.846477	2.928956	-0.219464	H	3.057499	2.769664	-0.529139	H	3.064221	2.761045	-0.536474
H	-0.225256	0.944148	-1.73371	H	0.113282	1.014688	-1.633091	H	0.110877	1.019486	-1.633799
H	1.002436	1.994301	-1.046794	H	-0.928926	2.124297	-0.754517	H	-0.925586	2.128087	-0.746787
H	0.525832	3.120332	1.214131	H	-0.047536	3.213783	1.382607	H	-0.038454	3.217508	1.37943
H	-1.244623	3.584617	1.458711	H	1.770656	3.539791	1.369849	H	1.780944	3.536794	1.367593
H	2.01514	-0.32539	-1.748432	H	-1.787314	-0.7001	-1.378634	H	-1.783591	-0.705082	-1.374515
H	1.696778	-2.553299	-1.344904	H	-2.656734	-2.475433	-0.089181	H	-2.653853	-2.474037	-0.07806
H	2.885807	-2.475529	-0.052016	H	-2.854545	-1.490144	1.349911	H	-2.861158	-1.480405	1.353933
H	1.140484	-2.060567	1.619548	H	-0.71595	-2.027704	2.082492	H	-0.725036	-2.004304	2.102136
H	0.3011	-3.186463	0.538304	H	-0.469205	-3.069085	0.684056	H	-0.476281	-3.064301	0.717707
H	4.188591	-0.525439	-0.722576	H	-2.975422	1.365022	-1.111812	H	-2.968971	1.362333	-1.121451
H	4.687674	1.838903	-0.394167	H	-4.497024	1.854991	0.774221	H	-4.493905	1.865004	0.758845
H	3.471237	1.688872	-1.670135	H	-2.825552	1.793207	1.335371	H	-2.823586	1.805256	1.323123
H	3.002127	2.216365	-0.045666	H	-3.921355	0.442881	1.658525	H	-3.921335	0.457834	1.652628
H	4.724287	0.203278	1.552646	H	-5.277857	0.508183	-1.215609	H	-5.272587	0.508345	-1.224447
H	3.588445	-1.143615	1.635809	H	-4.180818	-0.625531	-2.018642	H	-4.175686	-0.630804	-2.019693
H	3.02962	0.511345	1.921349	H	-4.804579	-0.986838	-0.402814	H	-4.803066	-0.983028	-0.403128
H	-4.170776	-1.478681	-0.237806	H	4.048572	-1.735217	-0.599247	H	3.121807	-2.509799	0.016331
**1d3**				**1d4**				**1d5**			
C	0.201907	-1.049877	0.363336	C	0.200753	-1.049108	0.372215	C	0.163128	-1.078043	0.221025
C	-0.726535	0.177508	0.170366	C	-0.724571	0.178509	0.170471	C	-0.789888	0.123975	-0.014766
C	1.485117	-0.800287	1.159717	C	1.488683	-0.802125	1.156126	C	1.352875	-0.812747	1.147019
C	2.570195	-0.623563	0.078085	C	2.579624	-0.617178	0.066051	C	2.527786	-0.548961	0.183537
C	3.247402	0.757021	0.007142	C	3.253604	0.760116	0.023374	C	3.148605	0.858911	0.227734
C	2.411134	1.896993	-0.61881	C	2.416456	1.890668	-0.61554	C	2.331133	1.987725	-0.441451

C	1.005195	2.10428	-0.078195	C	1.009085	2.102837	-0.079668	C	0.871915	2.123096	-0.036502
C	-0.148294	1.315764	-0.699097	C	-0.145955	1.315963	-0.700096	C	-0.184032	1.315138	-0.790636
C	0.778149	3.008069	0.88295	C	0.780627	3.008183	0.879822	C	0.513425	2.982426	0.925464
C	-2.07785	-0.418148	-0.345625	C	-2.076433	-0.417481	-0.34446	C	-2.06588	-0.501667	-0.666776
C	-2.001859	-1.946984	-0.014871	C	-2.003245	-1.944788	-0.007359	C	-1.949111	-2.039932	-0.412666
C	-0.757927	-2.125508	0.872203	C	-0.763066	-2.119081	0.885706	C	-0.796074	-2.212776	0.589998
C	-3.35475	0.230144	0.252654	C	-3.353367	0.234139	0.250461	C	-3.431358	0.071112	-0.192168
C	-4.619107	-0.317661	-0.431723	C	-4.617141	-0.313831	-0.434927	C	-3.655651	1.534941	-0.6014
C	-3.380883	1.765253	0.194686	C	-3.377881	1.769103	0.189412	C	-3.701059	-0.104167	1.312059
H	-0.894317	0.579376	1.18063	H	-0.89283	0.58309	1.179613	H	-1.052597	0.487988	0.987364
O	0.695289	-1.574391	-0.89483	O	0.698964	-1.585365	-0.880756	O	0.796858	-1.531539	-1.001794
O	1.89178	-0.781686	-1.172612	O	1.878072	-0.771709	-1.178352	O	1.980751	-0.684404	-1.132613
O	3.518204	-1.645199	0.292259	O	3.613508	-1.564946	0.187286	O	3.494668	-1.53985	0.452116
H	1.754698	-1.674722	1.756099	H	1.753724	-1.671081	1.76621	H	1.602023	-1.699548	1.733936
H	1.405133	0.066905	1.81633	H	1.417725	0.063377	1.816351	H	1.173365	0.023046	1.82447
H	3.559355	1.019873	1.023558	H	3.53821	1.025439	1.047116	H	3.347262	1.096212	1.27833
H	4.169136	0.655021	-0.580626	H	4.182545	0.647976	-0.544292	H	4.127573	0.815159	-0.267495
H	2.348679	1.720224	-1.697389	H	2.358143	1.704732	-1.692747	H	2.381691	1.847179	-1.525925
H	2.980903	2.82291	-0.48148	H	2.985368	2.817785	-0.484293	H	2.846477	2.928956	-0.219464
H	0.158935	0.893207	-1.658933	H	0.158992	0.894996	-1.66115	H	0.225256	0.944148	-1.73371
H	-0.958097	2.01991	-0.909539	H	-0.956221	2.020489	-0.907173	H	-1.002436	1.994301	-1.046794
H	-0.219969	3.200046	1.268904	H	-0.218276	3.202898	1.262611	H	-0.525832	3.120332	1.214131
H	1.582707	3.595614	1.317739	H	1.584985	3.595175	1.31571	H	1.244623	3.584617	1.458711
H	-2.11746	-0.288113	-1.435428	H	-2.114684	-0.291817	-1.434705	H	-2.01514	-0.32539	-1.748432
H	-1.885942	-2.531553	-0.93188	H	-1.8834	-2.53348	-0.921173	H	-1.696778	-2.553299	-1.344904
H	-2.905984	-2.307714	0.483672	H	-2.909201	-2.303214	0.48931	H	-2.885807	-2.475529	-0.052016
H	-0.991434	-1.917598	1.923045	H	-0.999471	-1.903511	1.934265	H	-1.140484	-2.060567	1.619548
H	-0.310731	-3.121798	0.816798	H	-0.320711	-3.118675	0.839972	H	-0.3011	-3.186463	0.538304
H	-3.39161	-0.059005	1.315257	H	-3.392287	-0.052729	1.31365	H	-4.188591	-0.525439	-0.722576
H	-5.523337	0.082233	0.039576	H	-5.521622	0.087927	0.034193	H	-4.687674	1.838903	-0.394167
H	-4.678787	-1.408857	-0.389795	H	-4.678342	-1.404912	-0.391092	H	-3.471237	1.688872	-1.670135
H	-4.640334	-0.02567	-1.488822	H	-4.636378	-0.023788	-1.492557	H	-3.002127	2.216365	-0.045666
H	-4.314306	2.144359	0.624952	H	-4.311711	2.149475	0.617582	H	-4.724287	0.203278	1.552646
H	-2.557966	2.220505	0.752408	H	-2.555608	2.22485	0.7476	H	-3.588445	-1.143615	1.635809
H	-3.329308	2.125053	-0.839912	H	-3.324516	2.126993	-0.845694	H	-3.02962	0.511345	1.921349
H	4.126492	-1.638017	-0.460605	H	3.208165	-2.441385	0.115746	H	4.170776	-1.478681	-0.237806

Table S3. Calculated ^{13}C NMR chemical shifts for **1a**.

No.	**1a1**	**1a2**	**1a3**	**1a4**	**1a5**	**1a6**
1	35.2523	35.41	26.1684	26.2986	32.6373	32.9805
2	37.0144	36.7614	38.0516	37.9095	36.3566	36.0586

3	104.9012	105.2844	104.2063	104.6173	105.3647	105.6498
4	54.3011	51.2485	54.3891	51.1147	54.1606	50.919
5	115.088	113.7436	115.3219	113.8164	114.9313	113.6122
6	42.3717	45.2555	42.4407	45.2173	42.1852	45.0658
7	32.0634	33.0684	31.8604	32.8861	32.0003	32.9953
8	165.3782	165.3066	165.4338	165.3587	165.5551	165.5259
9	45.2072	45.0963	44.4643	44.3267	46.2978	46.1964
10	52.75	53.4826	50.8691	51.6025	45.0695	45.7676
11	59.4167	59.4525	59.4519	59.4799	57.883	58.0145
12	42.581	42.6586	34.8496	34.7622	35.8356	35.939
13	22.8103	22.831	23.5171	23.5212	15.7807	15.8377
14	22.4086	22.4504	16.2172	16.2323	24.3689	24.4718
15	115.8088	115.8888	115.9306	115.9952	115.5483	115.6679

Table S4. Experimental and calculated ^{13}C NMR chemical shifts of **1a**.

No.	δ_{exp}	δ_{calc}	δ_{scalc}
1	28.0	30.5	28.2
2	33.2	37.3	34.5
3	98.0	104.8	97.4
4	49.2	52.8	48.9
5	107.4	114.5	106.4
6	39.3	43.7	40.5
7	29.1	32.4	30.0
8	149.4	165.4	153.8
9	40.8	45.0	41.7
10	46.8	50.8	47.1
11	54.3	59.2	54.9
12	32.8	37.7	34.8
13	22.0	21.9	20.1
14	20.1	19.8	18.2
15	113.0	115.9	107.6

δ_{calc}: unscaled chemical shifts of **1a** relative to TMS at the same level of theory;
δ_{scalc}: calculated chemical shifts of **1a** after linear scaling.

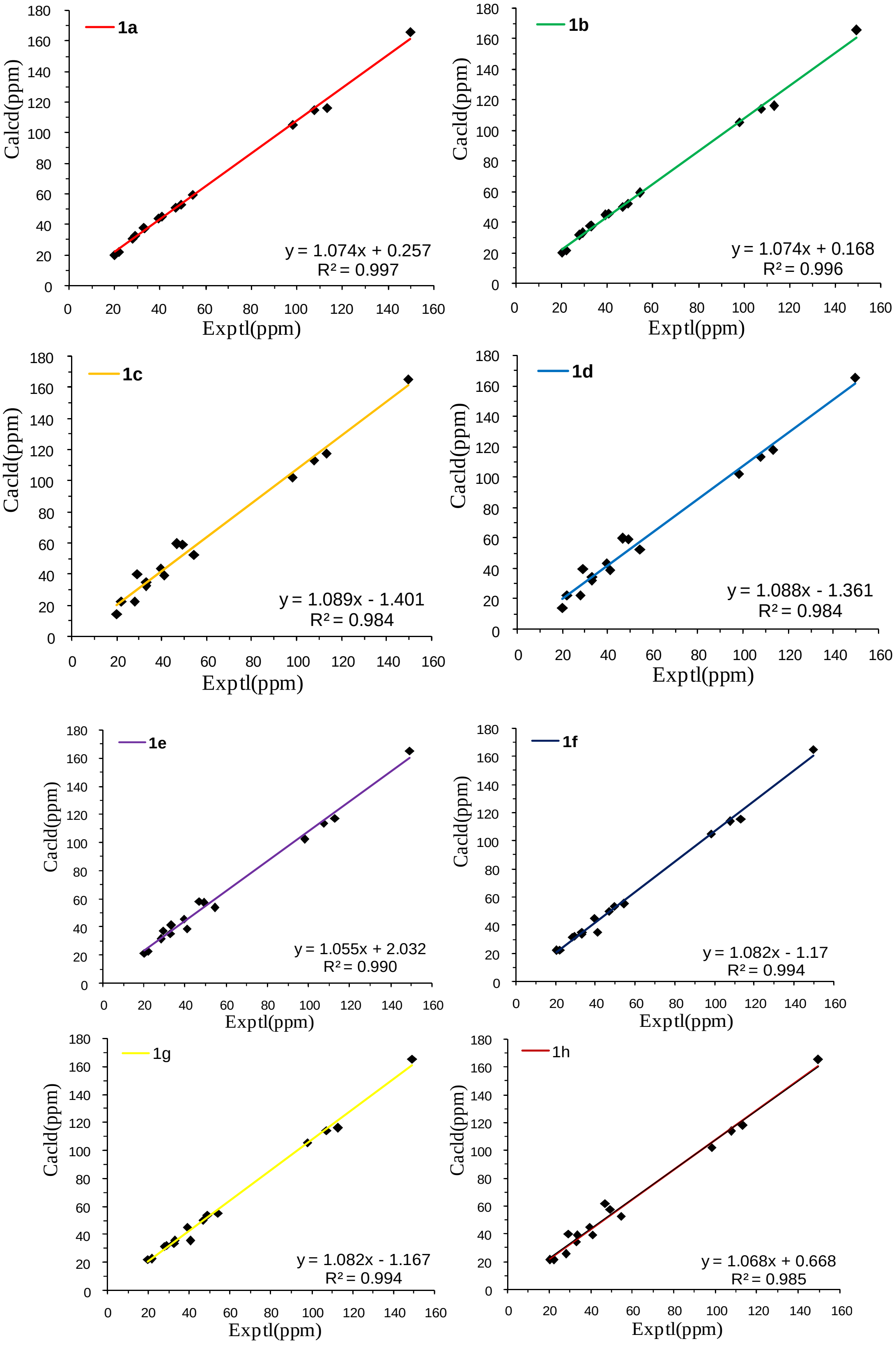

Figure S4. Correlation of experimental and calculated chemical shifts of compound **1** with **1a-1h**

NOTE: 1a: 3*R*,5*R*,10*S*,11*S*; **1b**: 3*S*,5*S*,10*R*,11*R*; **1c:** 3*R*,5*R*,10*R*,11*R*; **1d**: 3*S*,5*S*,10*S*,11*S*; **1e:** 3*R*,5*R*,10*R*,11*S*; **1f**: 3*R*,5*R*,10*S*,11*R*; **1g:** 3*S*,5*S*,10*R*,11*S*; **1h**: 3*S*,5*S*,10*S*,11*R*

3. ECD calculations details for 1 to determine the absolute configuration

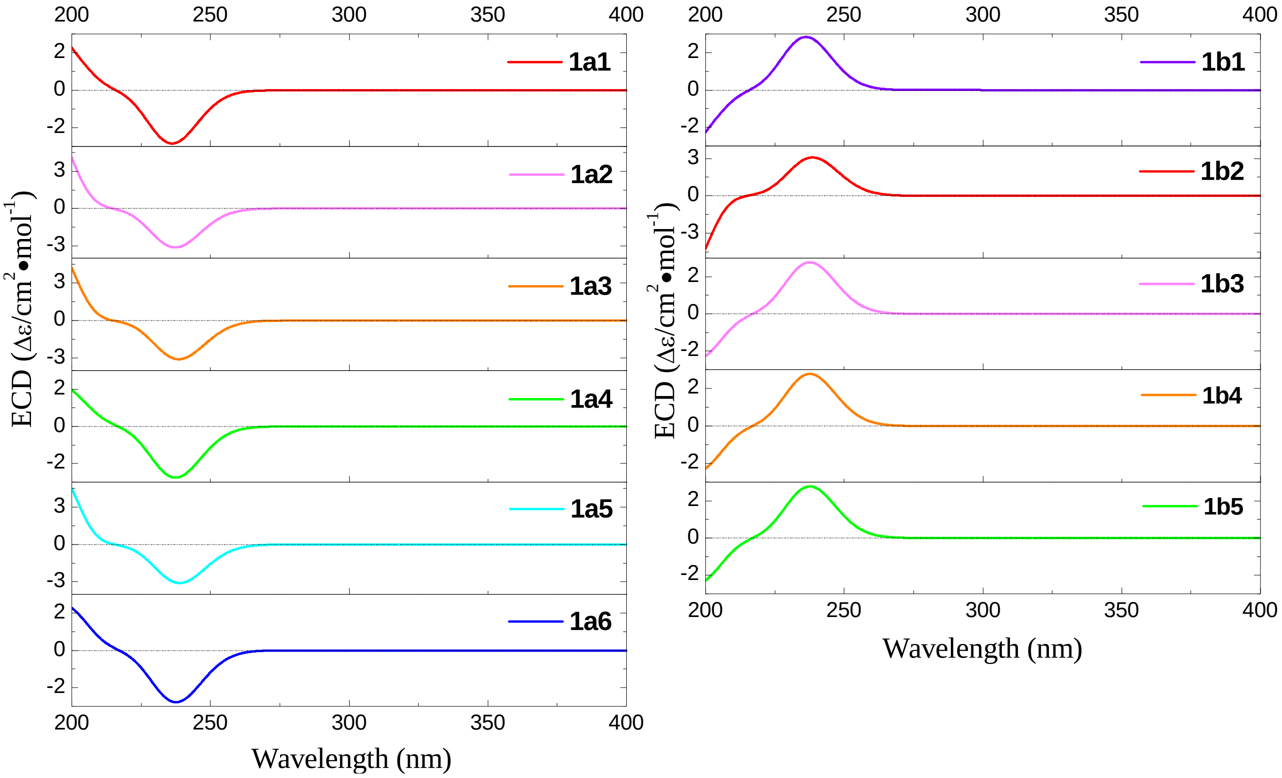

Figure S5. The corresponding calculated ECD of structural candidates with respective **1a** and **1b** configurations.

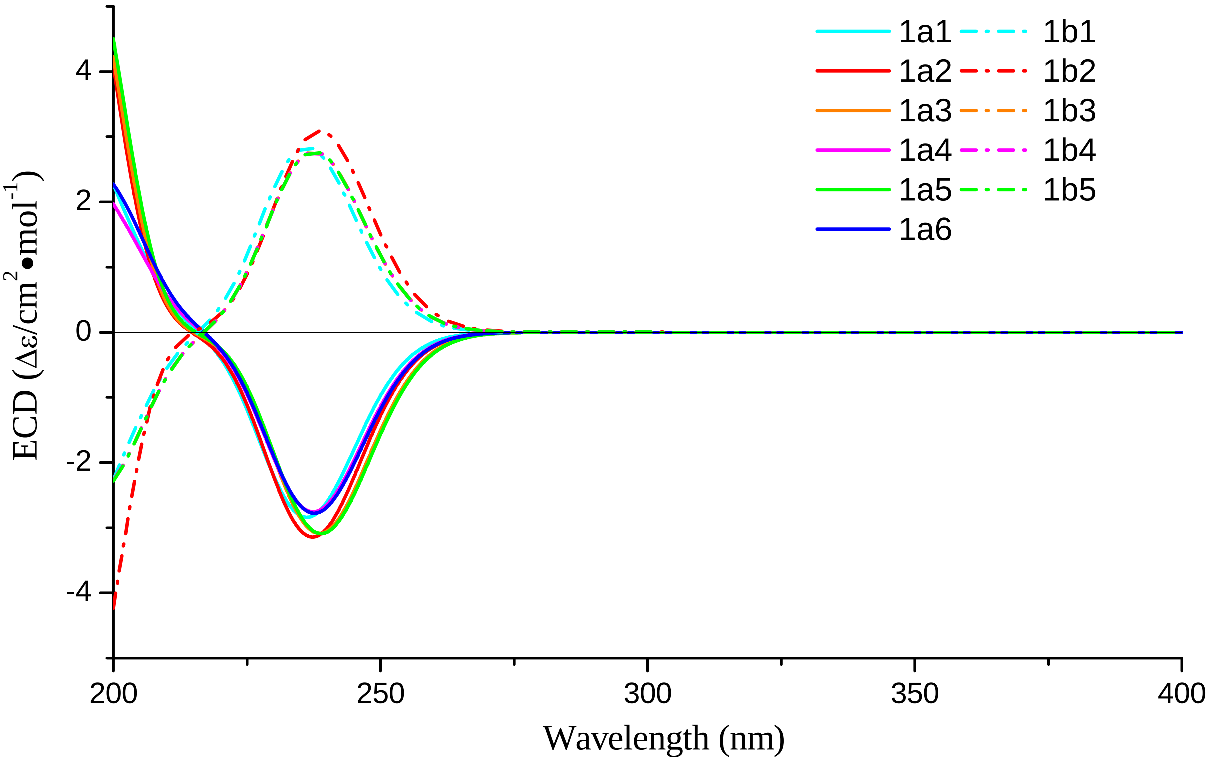

Figure S6. The overlaid ECD calculated for all the structural candidates.

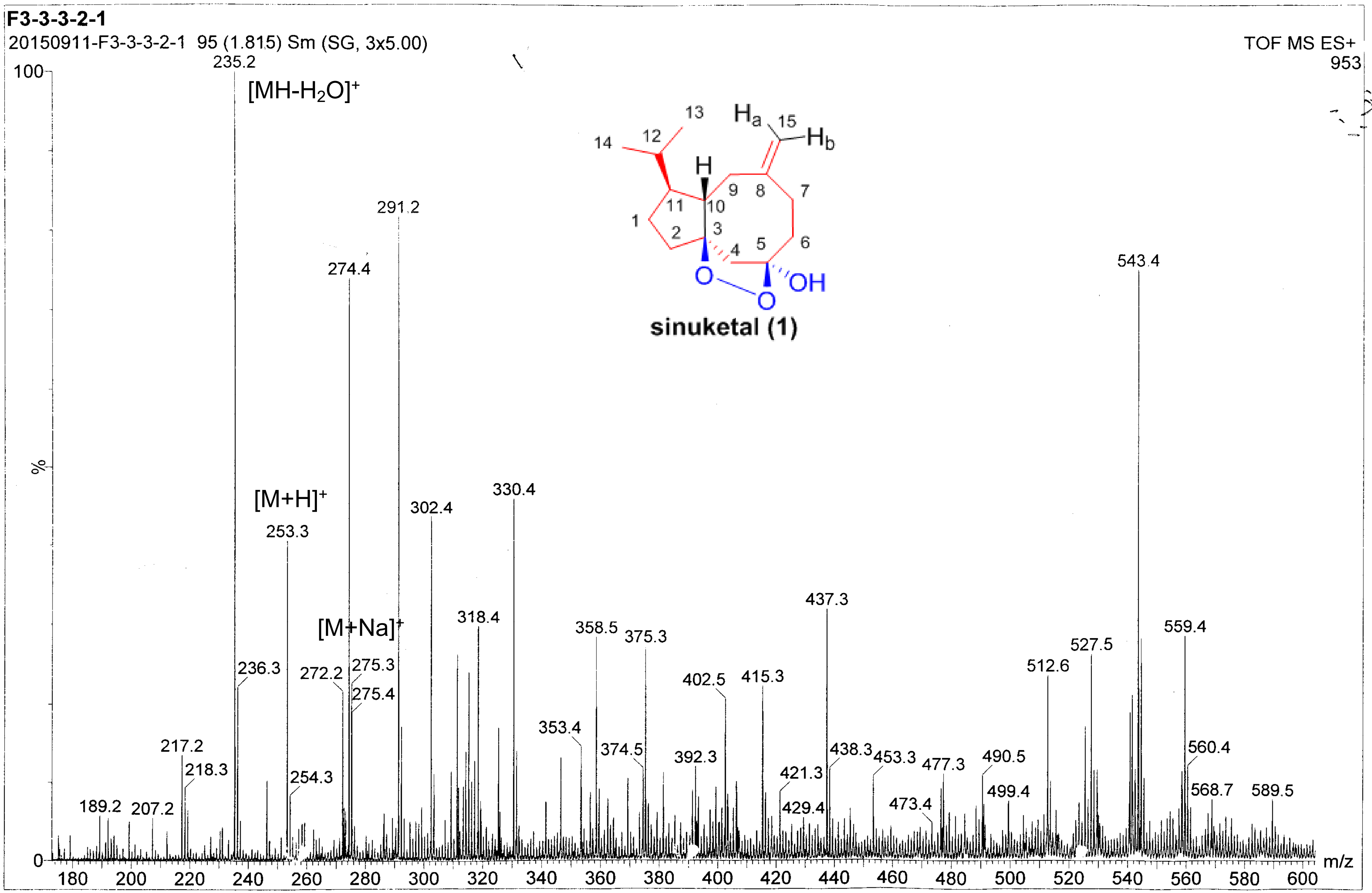

Figure SS1 The positive ESIMS spectrum of sinuketal (1)

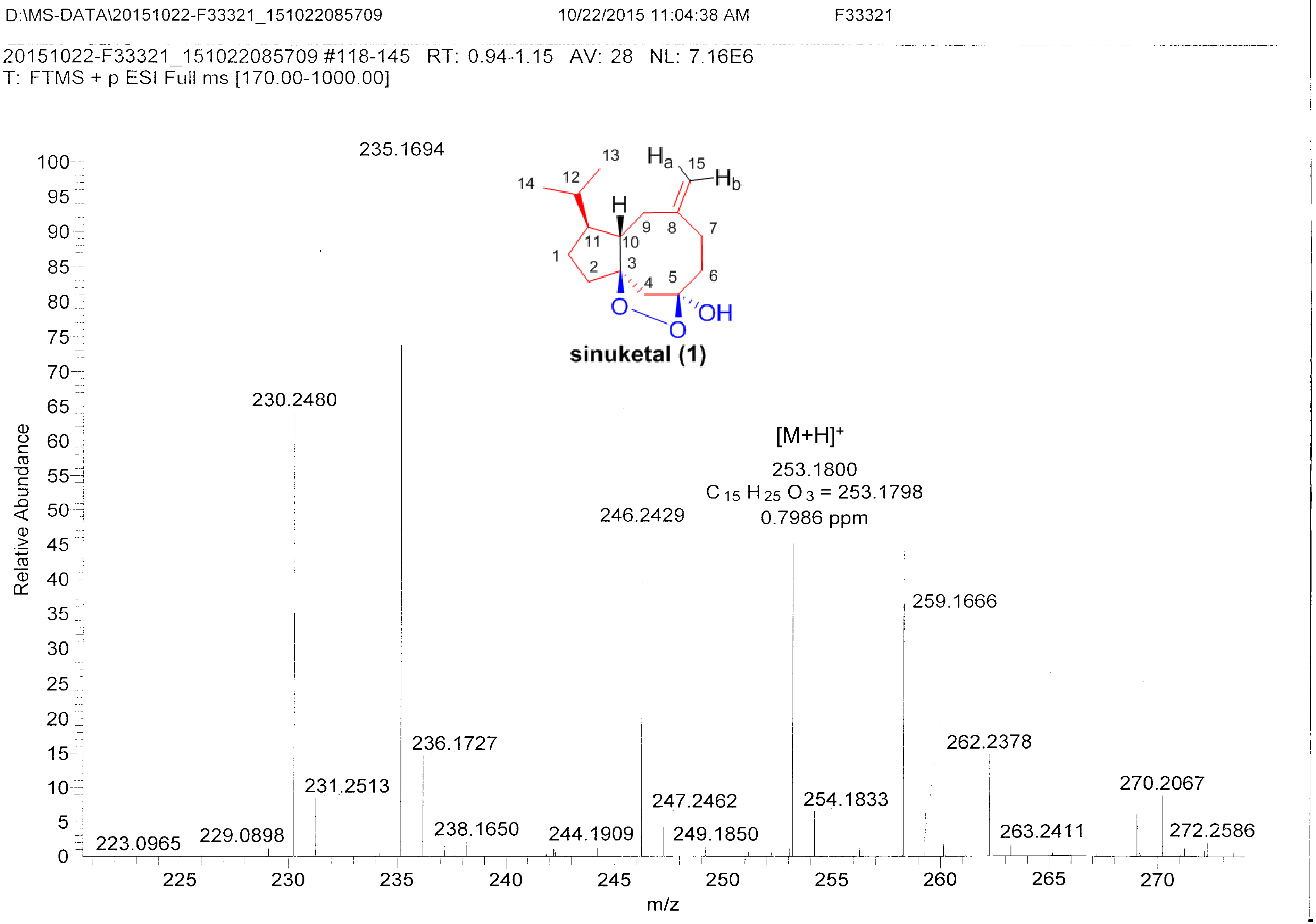

Figure SS2 The positive HRESIMS spectrum of sinuketal (1)

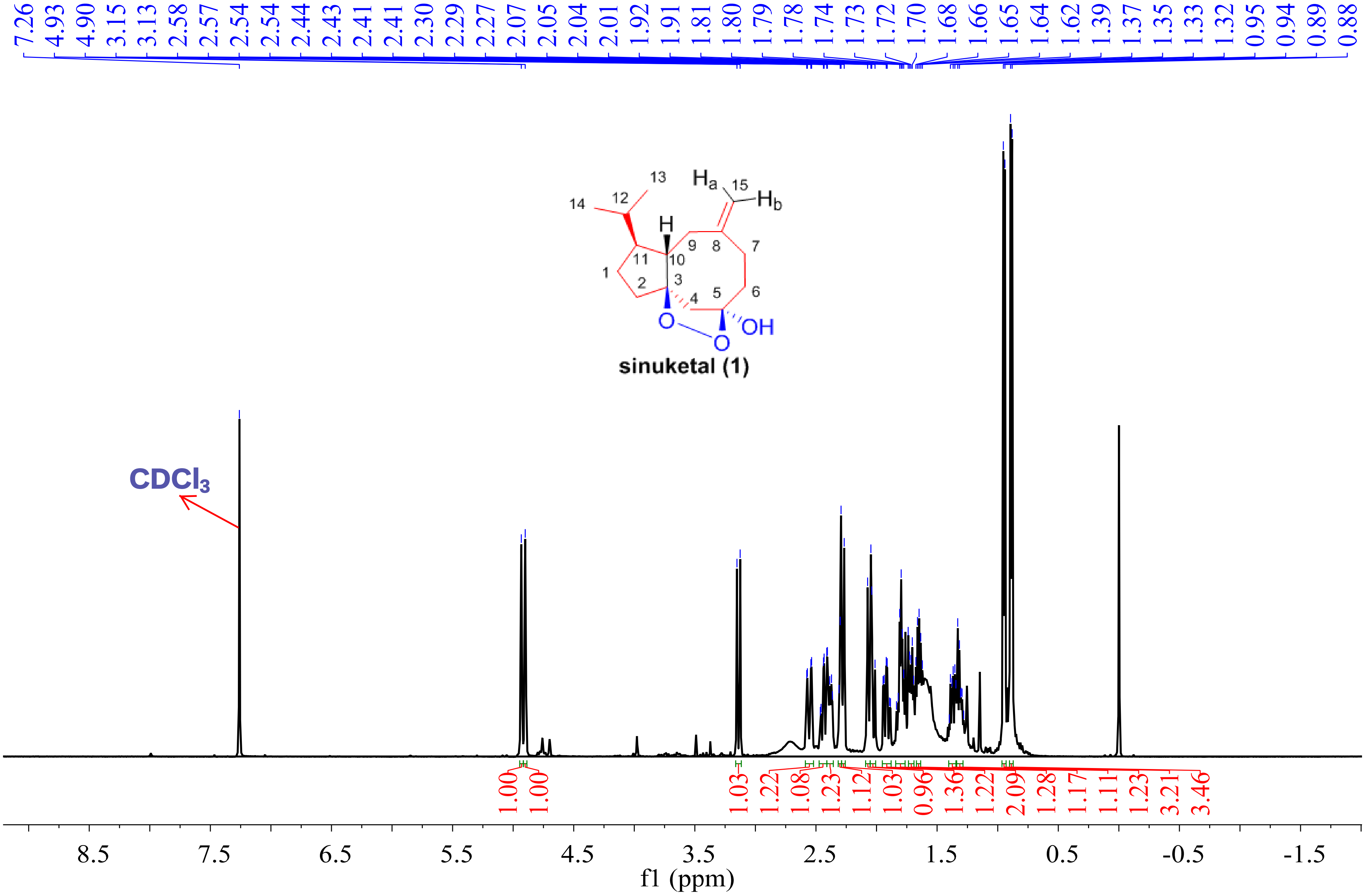

Figure SS3 ^{1}H NMR (500MHz, $CDCl_3$) spectrum of sinuketal (1)

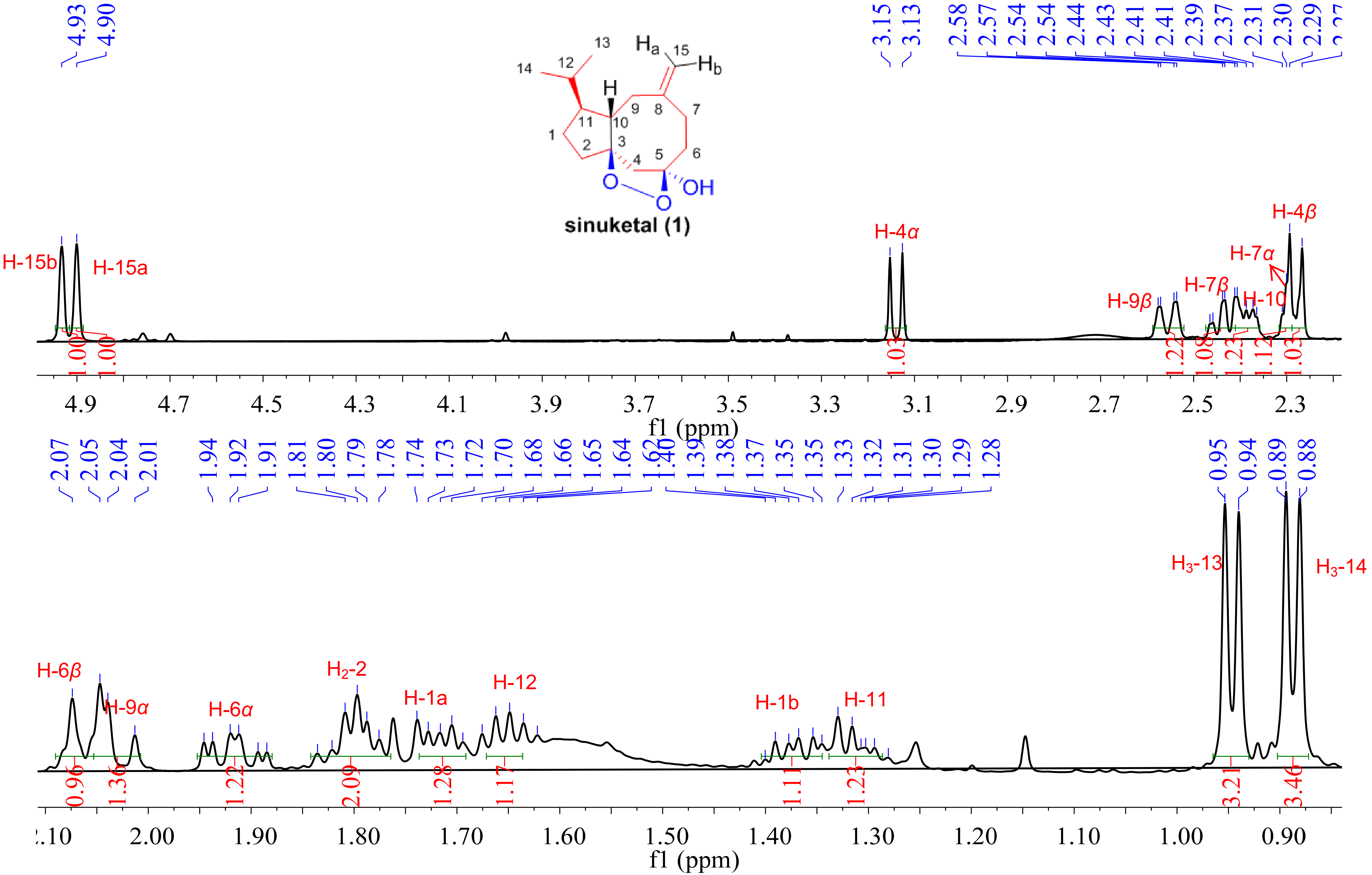

Figure SS4 Amplificatory ^{1}H NMR spectrum of sinuketal (1)

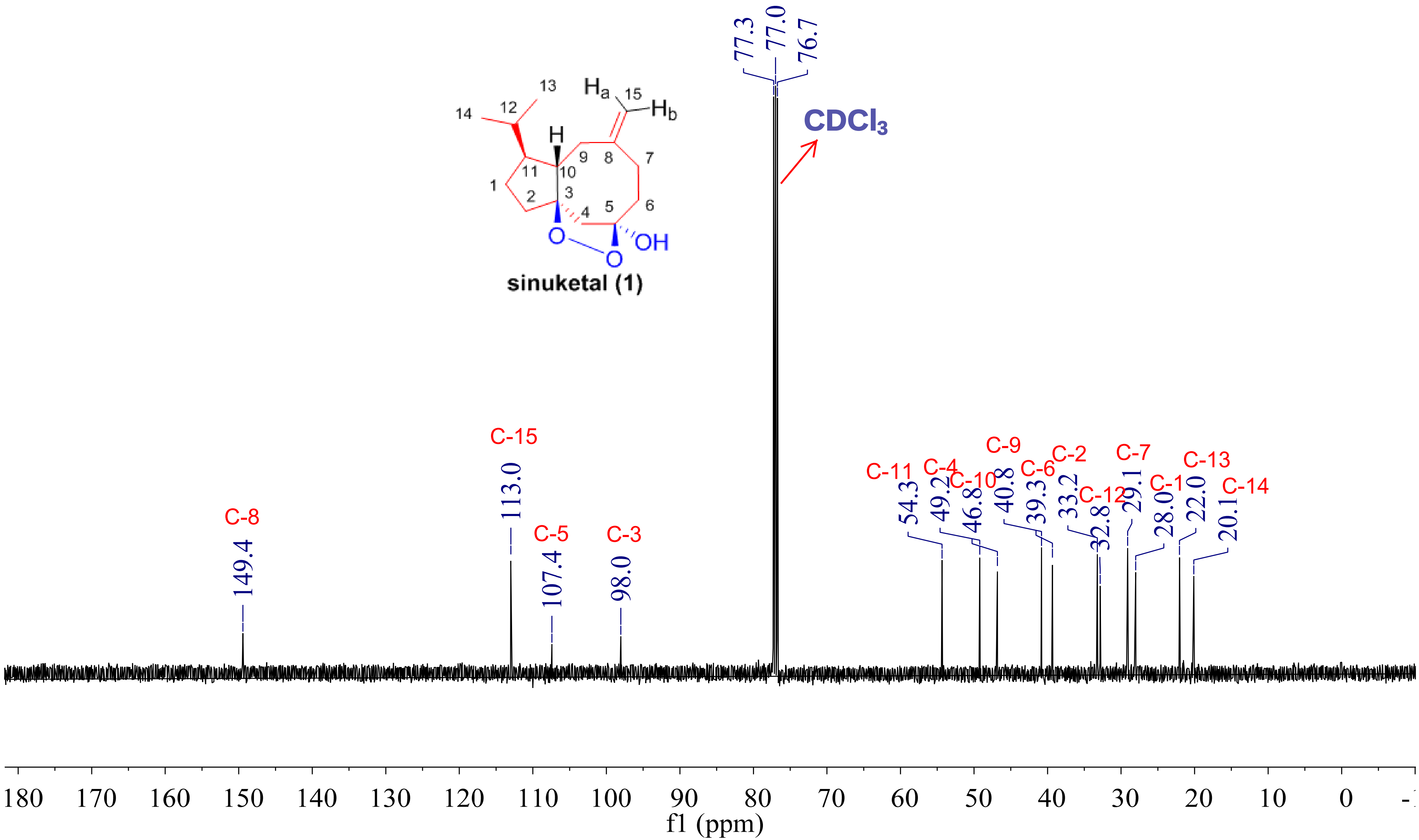

Figure SS5 ^{13}C NMR (125MHz, $CDCl_3$) spectrum of sinuketal (1)

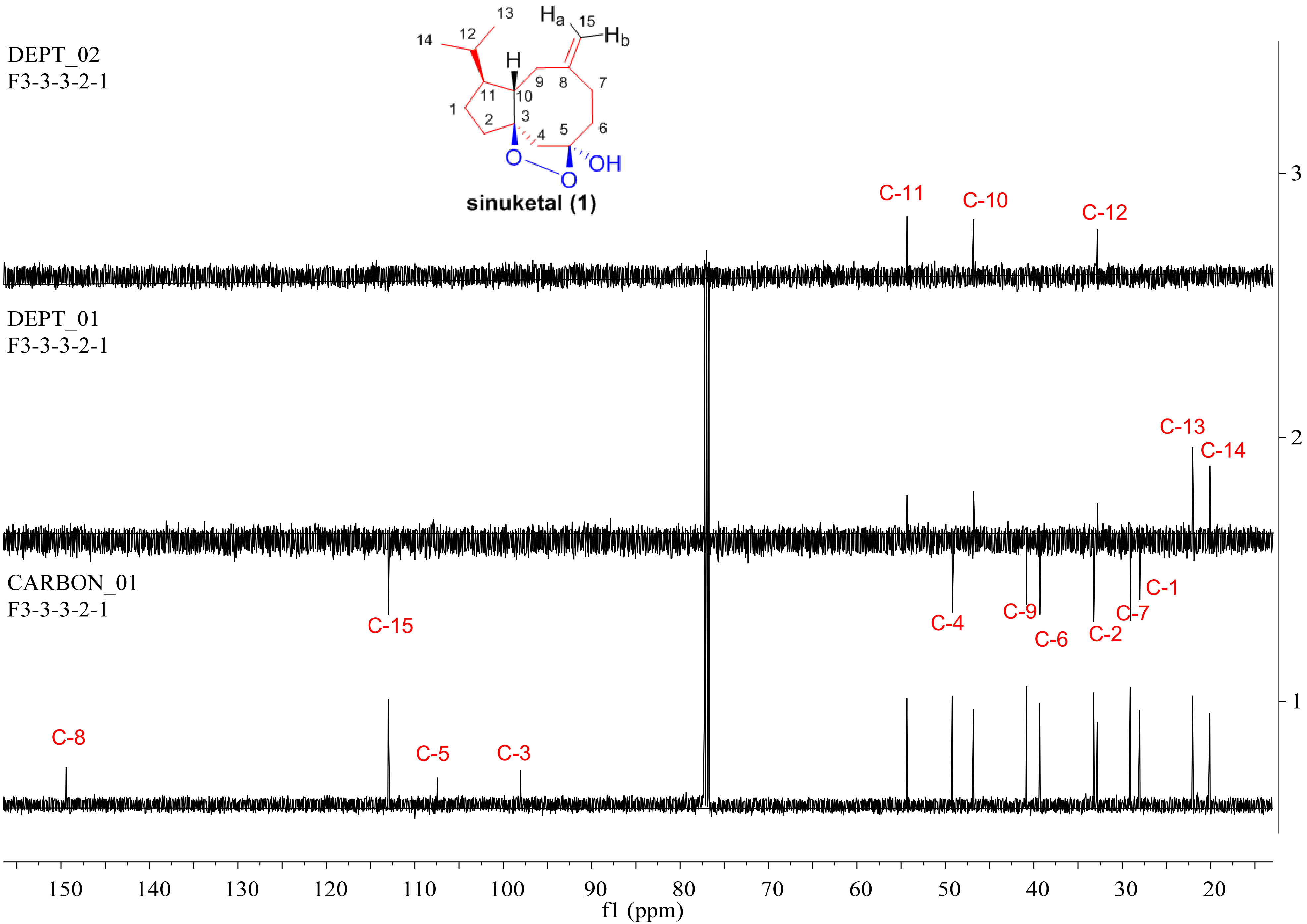

Figure SS6 DEPT spectrum of sinuketal (1)

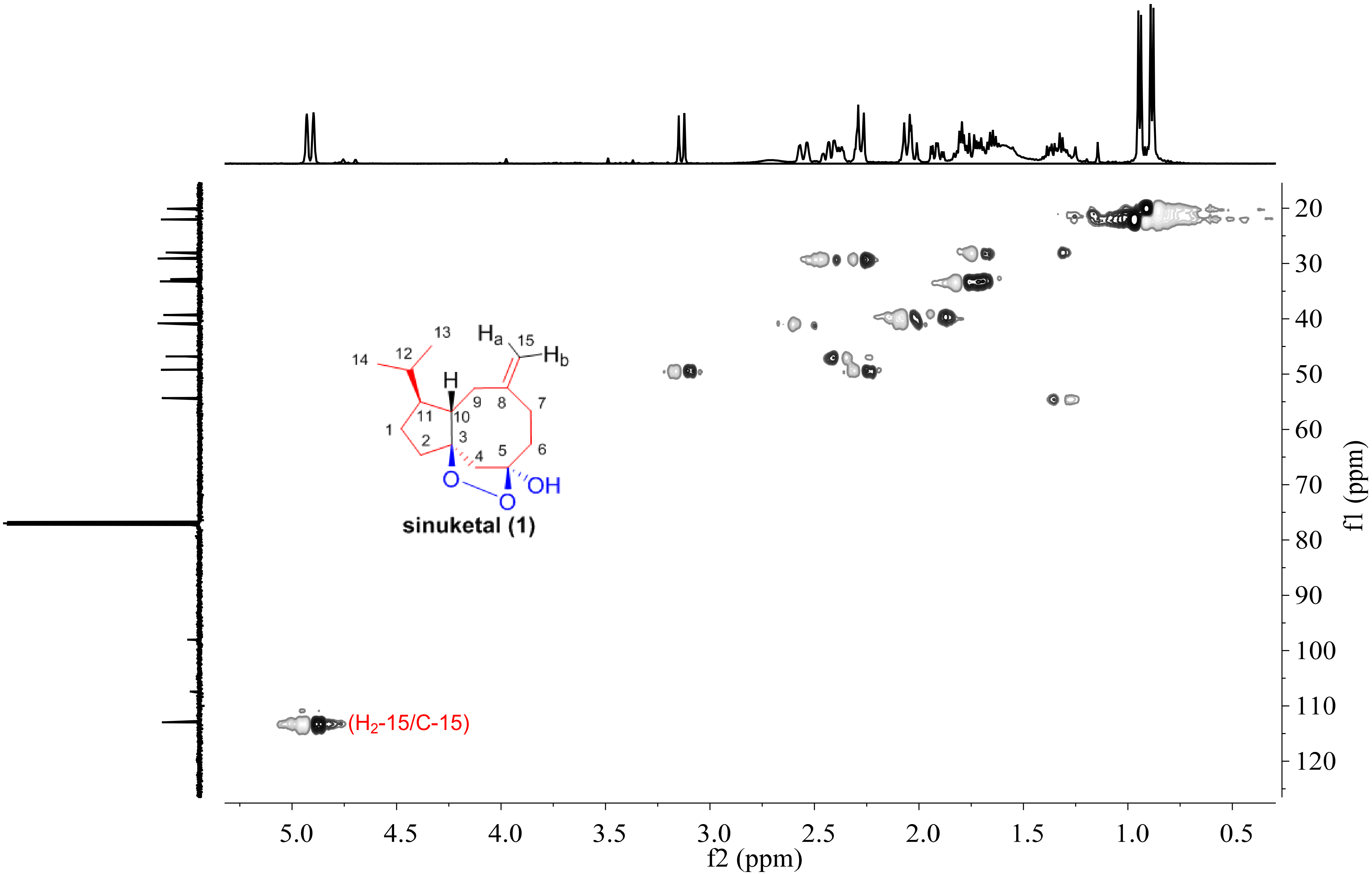

Figure SS7 HMQC spectrum of sinuketal (1)

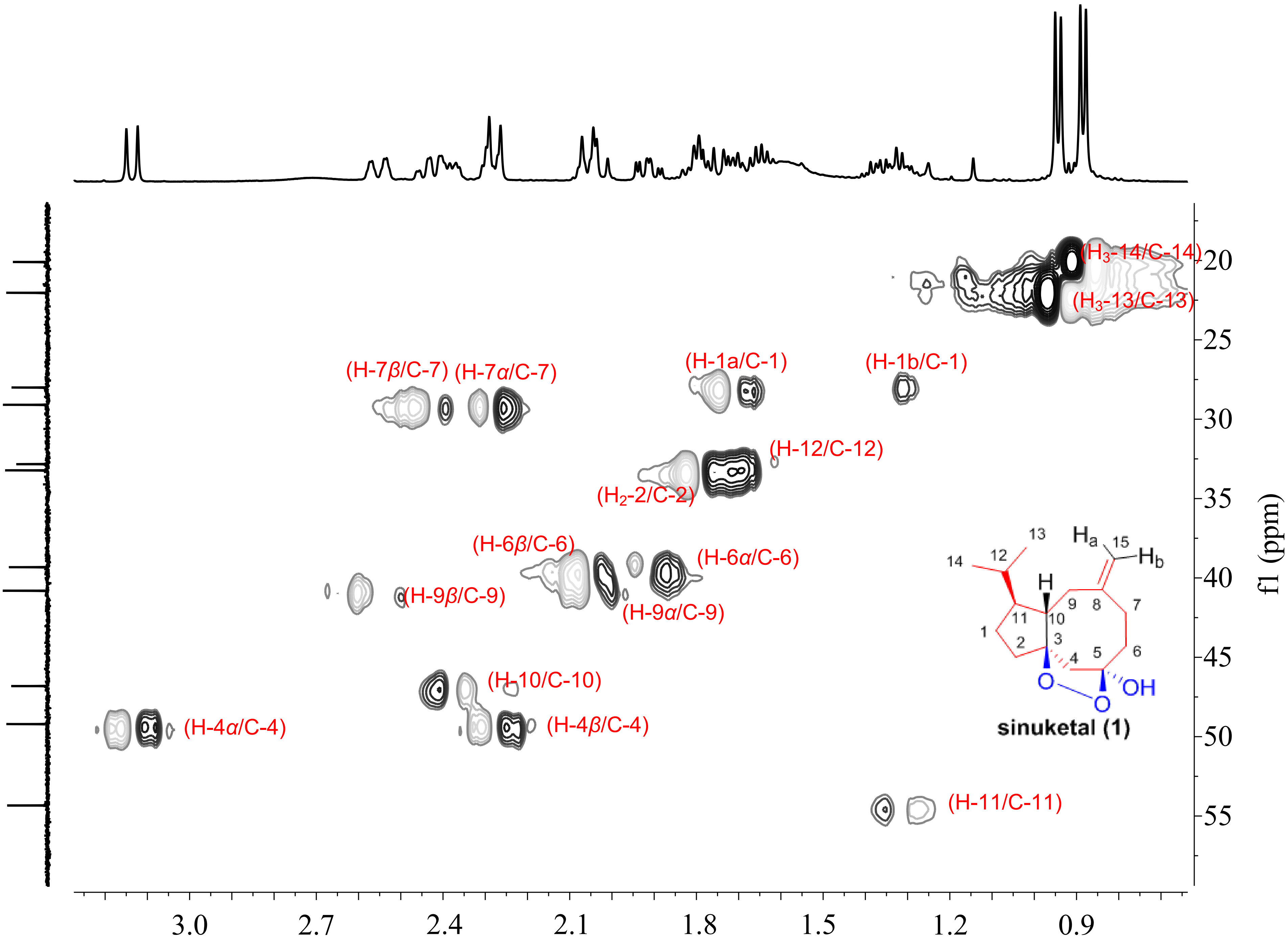

Figure SS8 Partial HMQC spectrum of sinuketal (1)

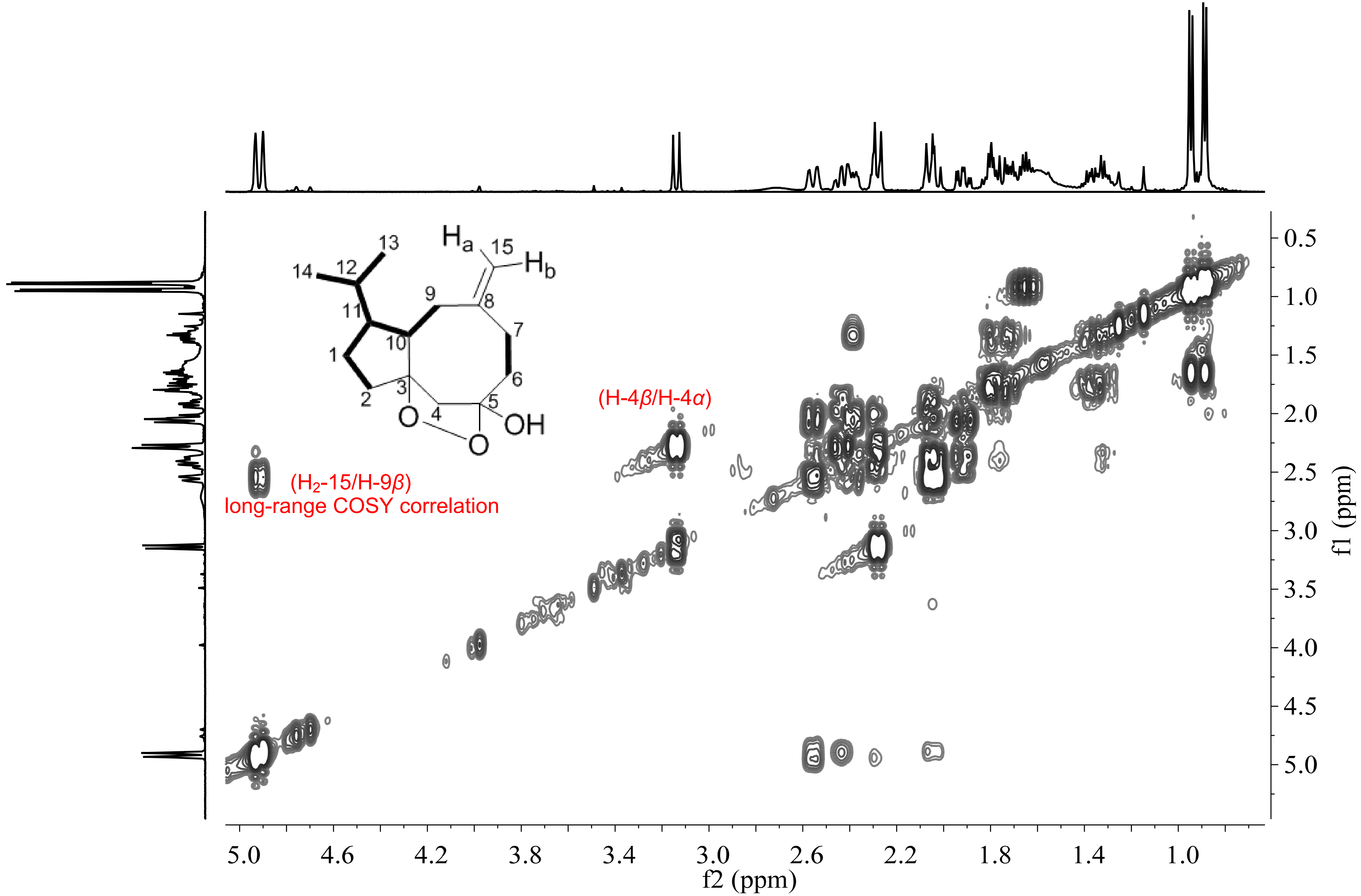

Figure SS9 ^{1}H-^{1}H COSY spectrum of sinuketal (1)

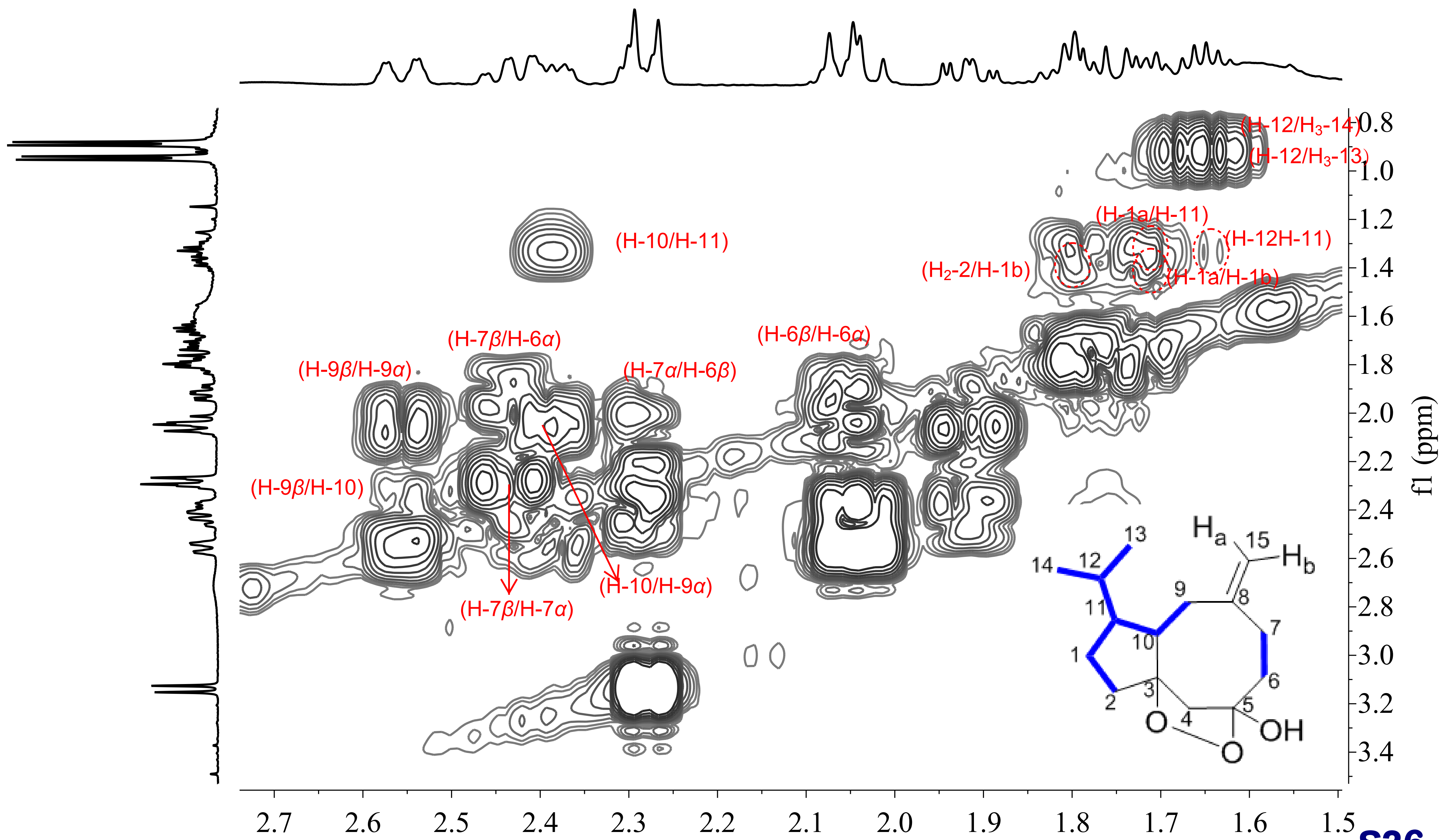

Figure SS10 Partial ^1H-^1H COSY spectrum of sinuketal (1)

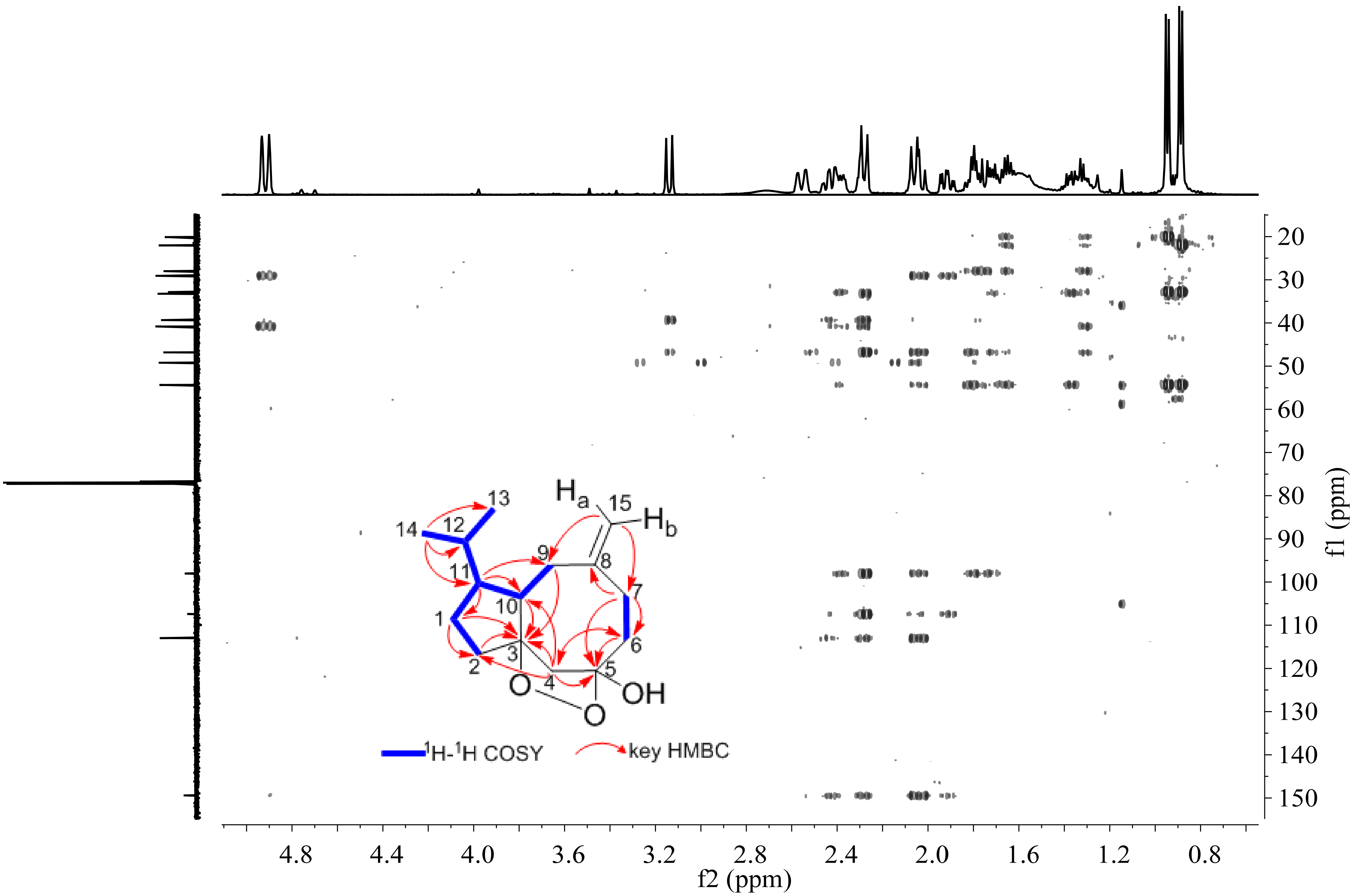

Figure SS11 Key HMBC spectrum of sinuketal (1)

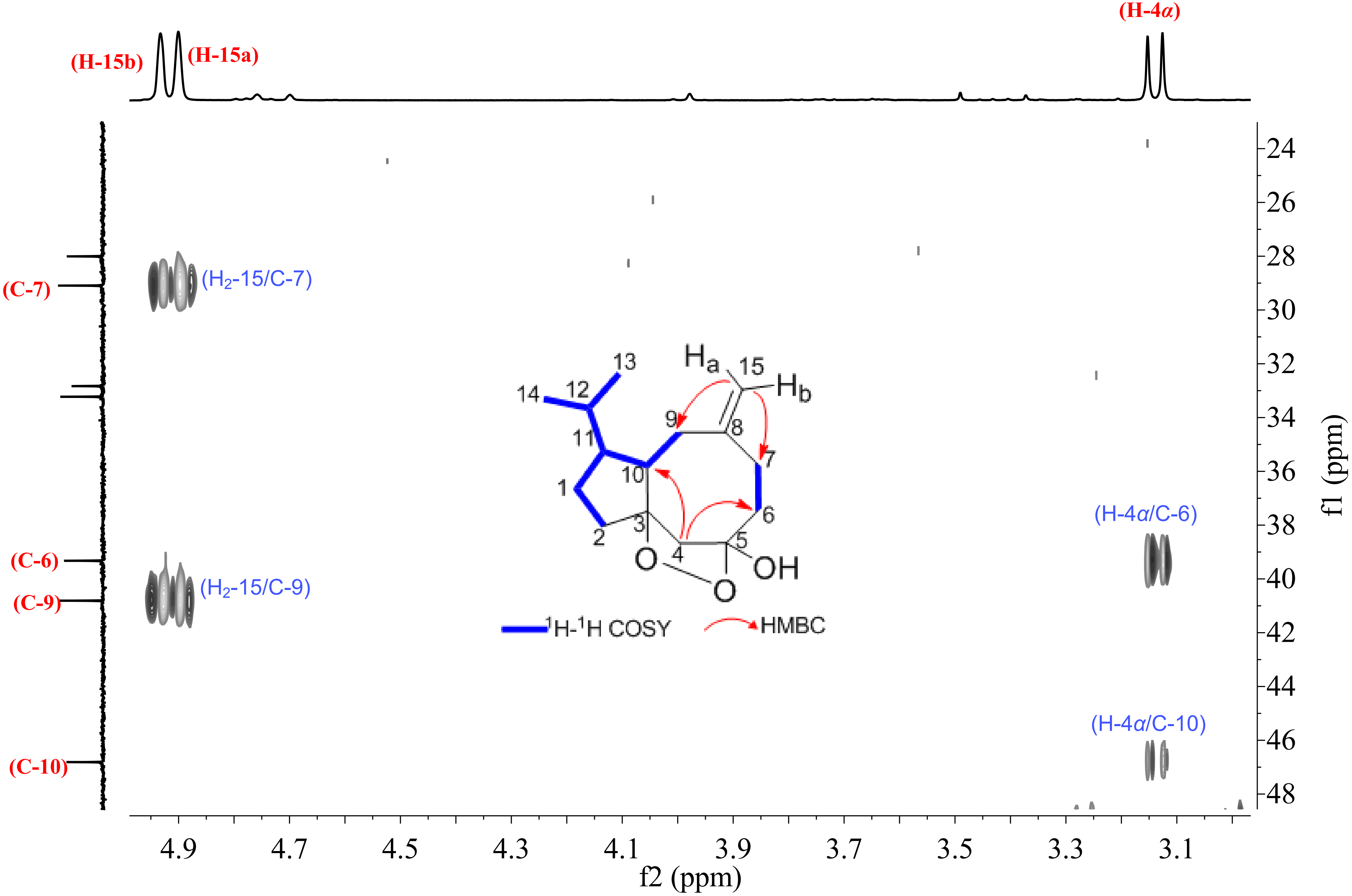

Figure SS12 Partial HMBC spectrum of sinuketal (1) (A)

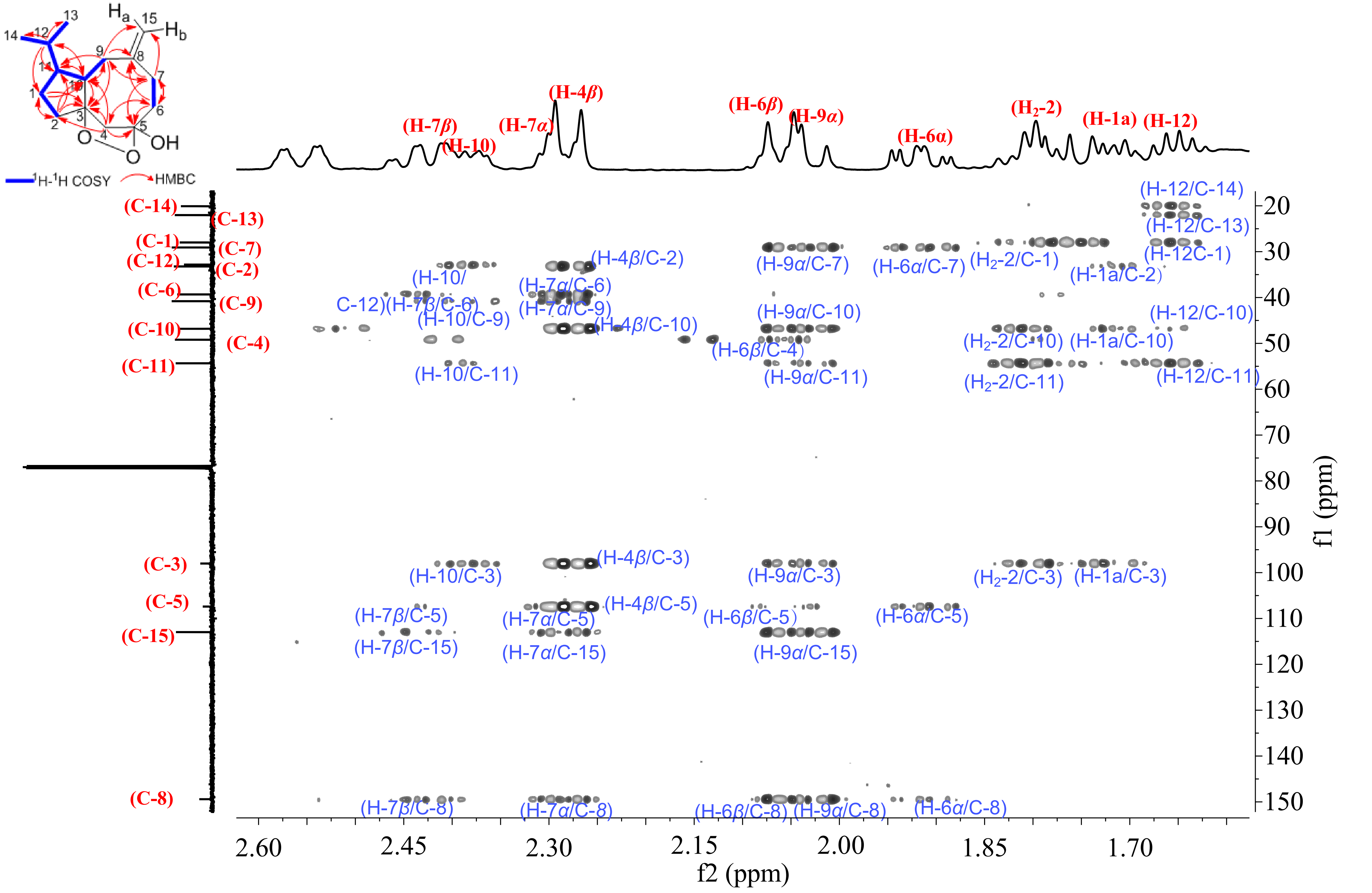

Figure SS13 Partial HMBC spectrum of sinuketal (1) (B)

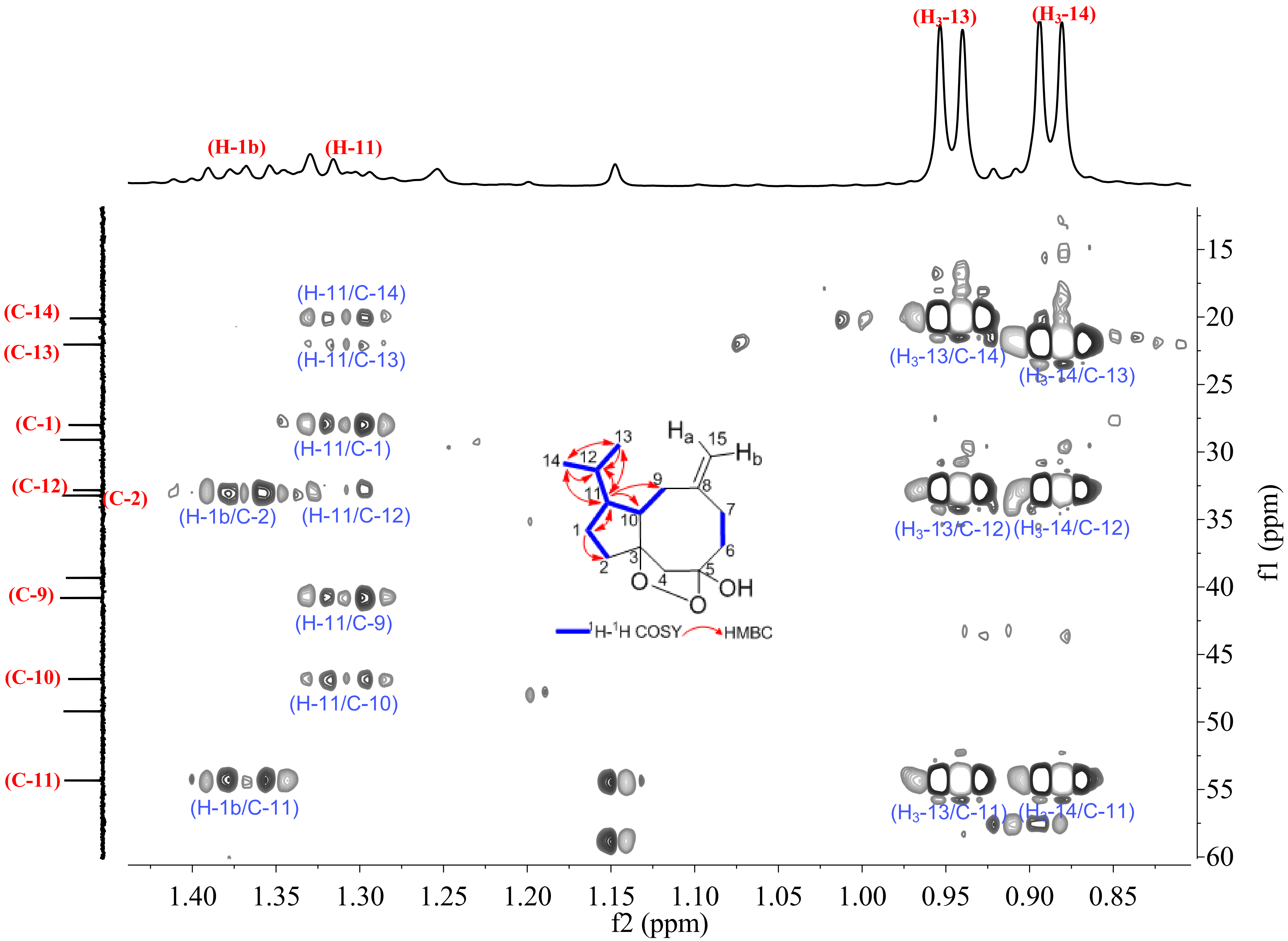

Figure SS14 Partial HMBC spectrum of sinuketal (1) (C)

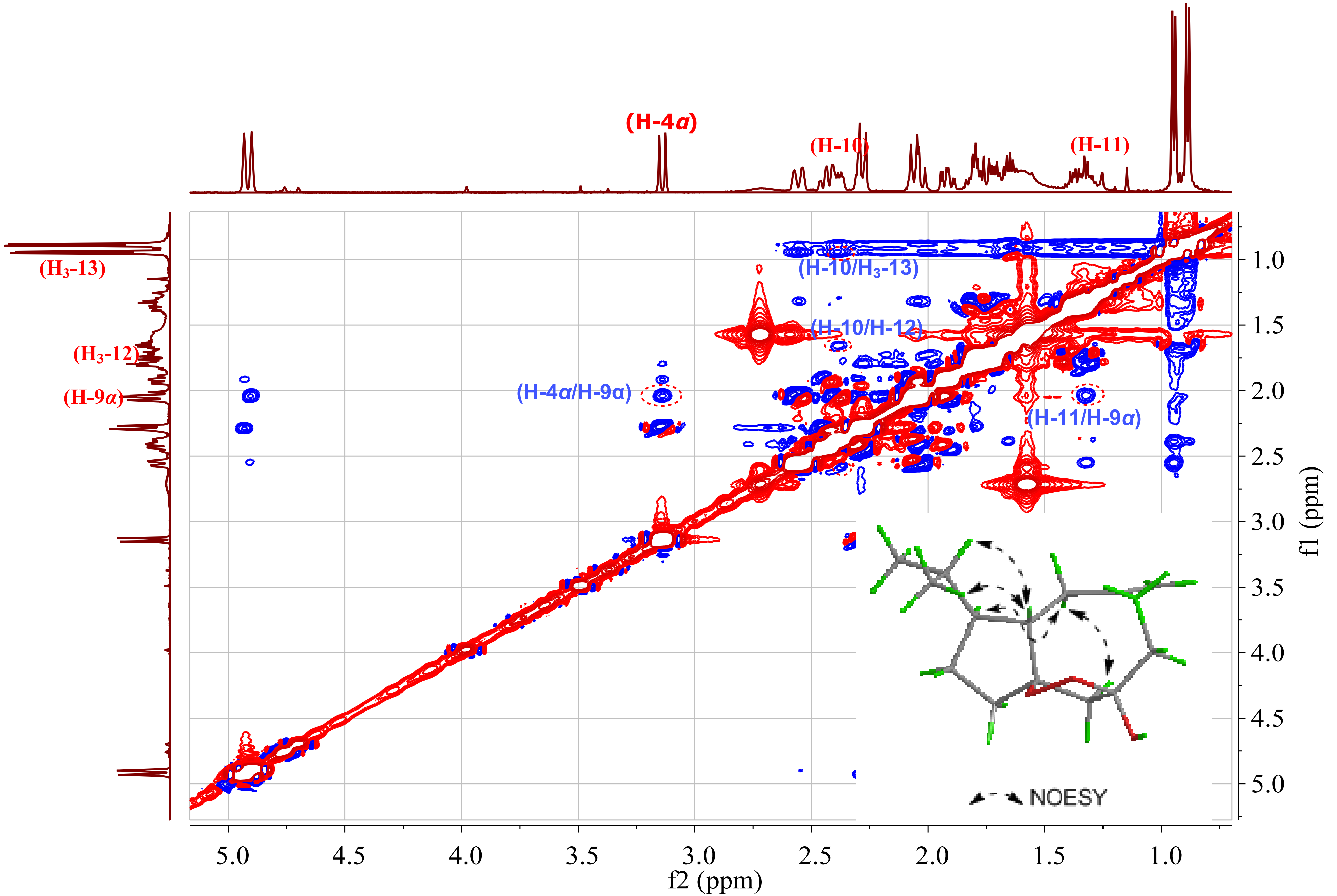

Figure SS15 NOESY spectrum of sinuketal (1)

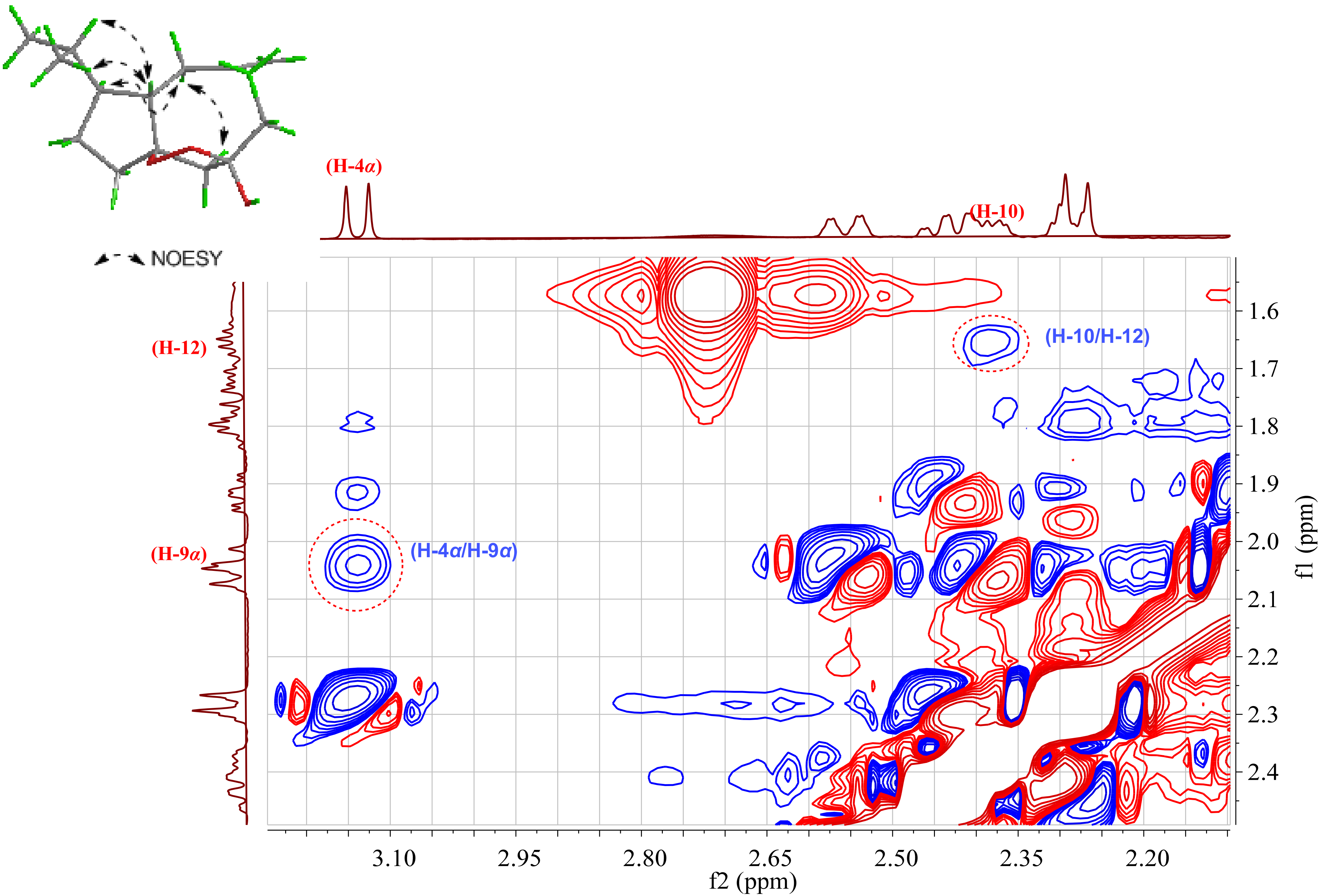

Figure SS16 Key amplificatory NOESY spectrum of sinuketal (1)

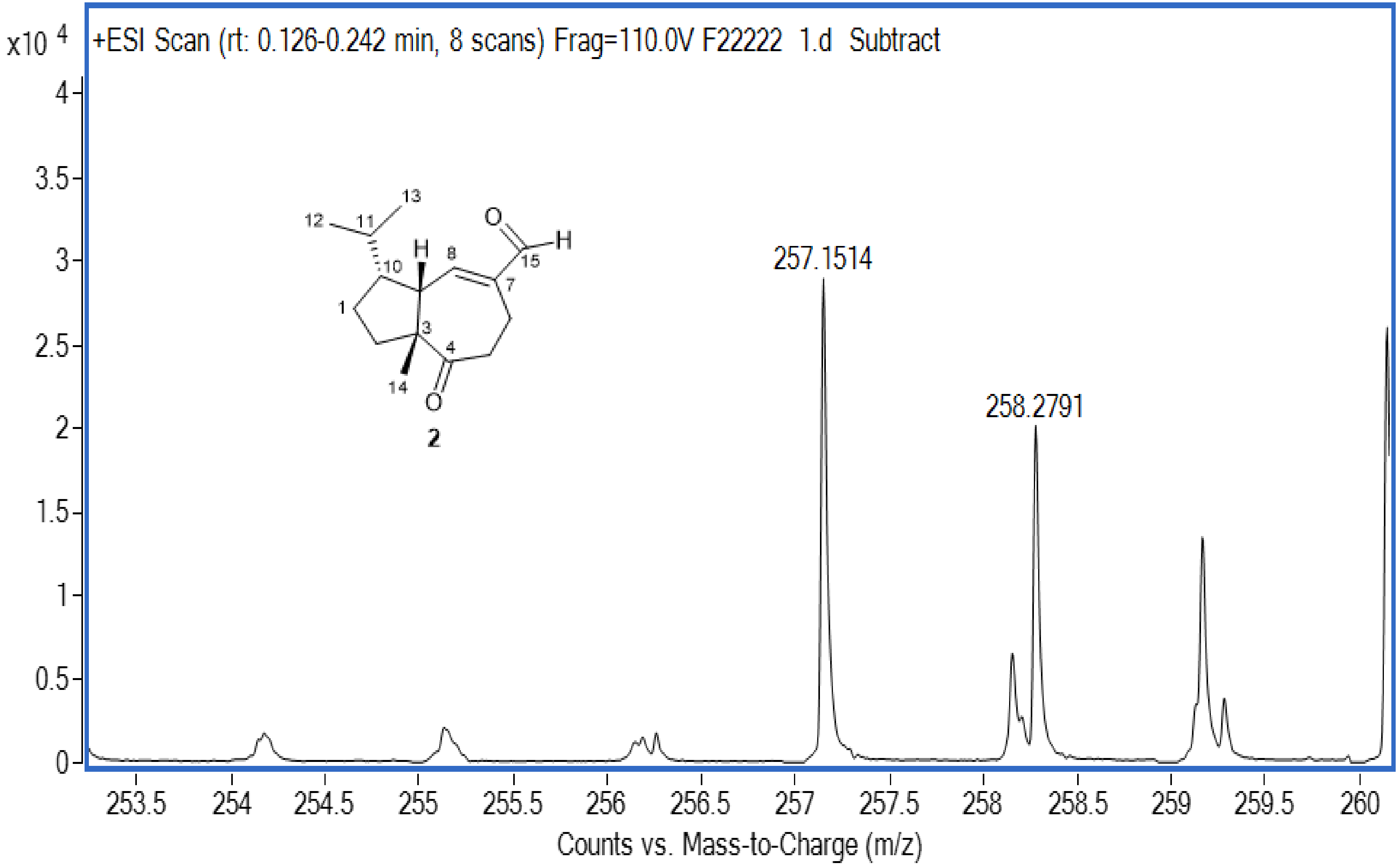

Figure SS17 The positive HRESIMS spectrum of sinulin A (2)

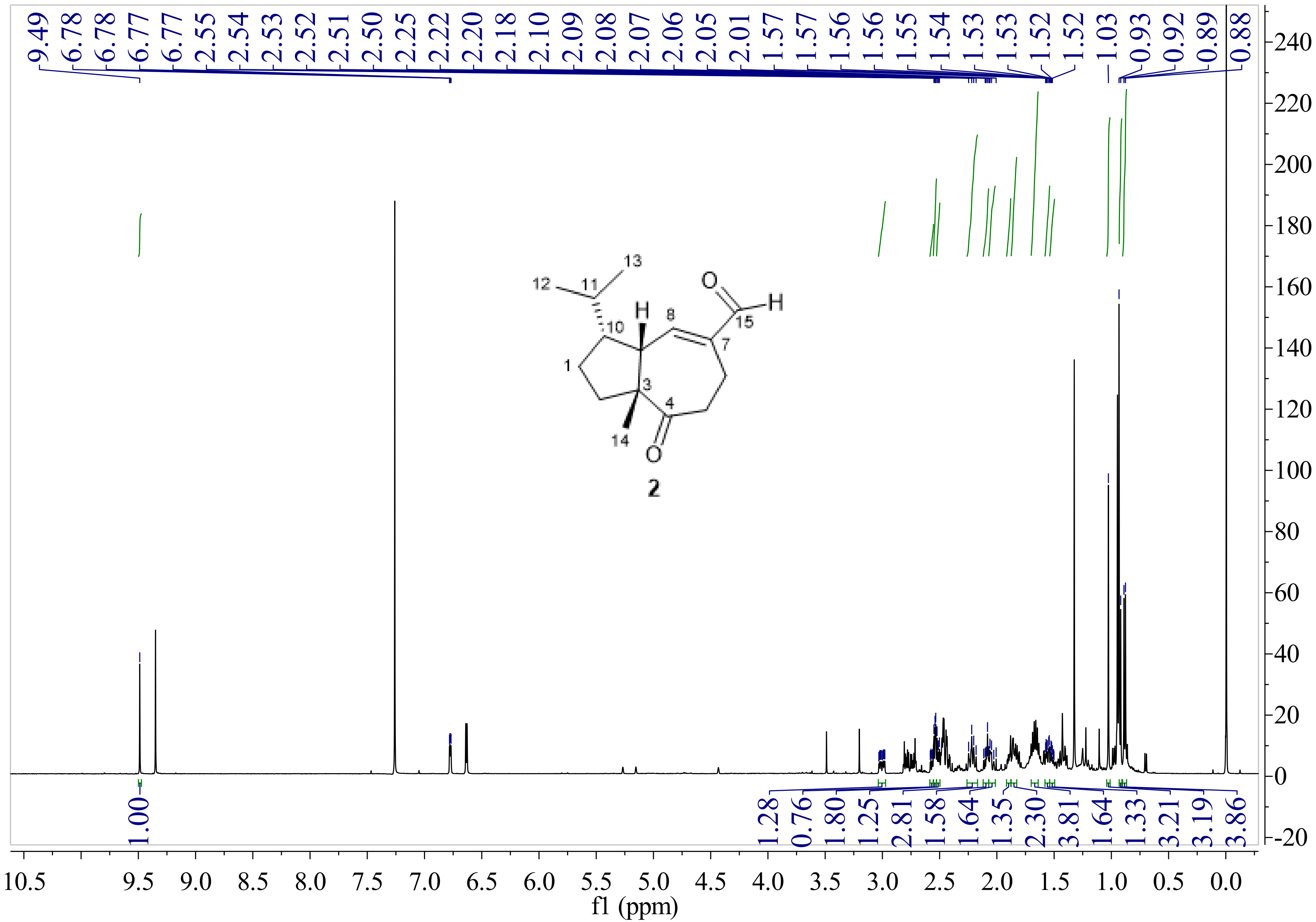

Figure SS18 ^{1}H NMR (500MHz, $CDCl_3$) spectrum of sinulin A (2) and compound (6)

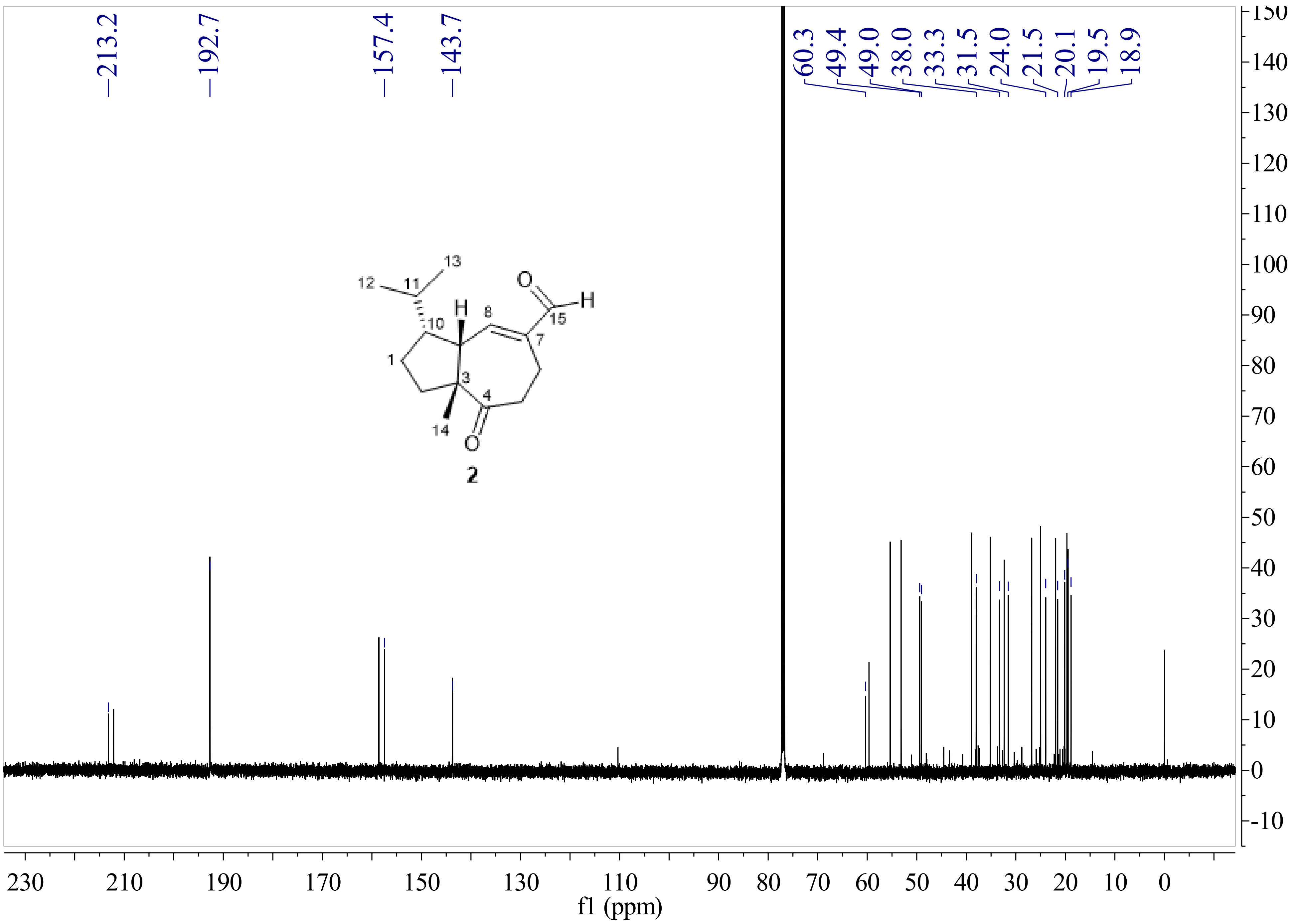

Figure SS19 ^{13}C NMR (125MHz, $CDCl_3$) spectrum of sinulin A (2) and compound (6)

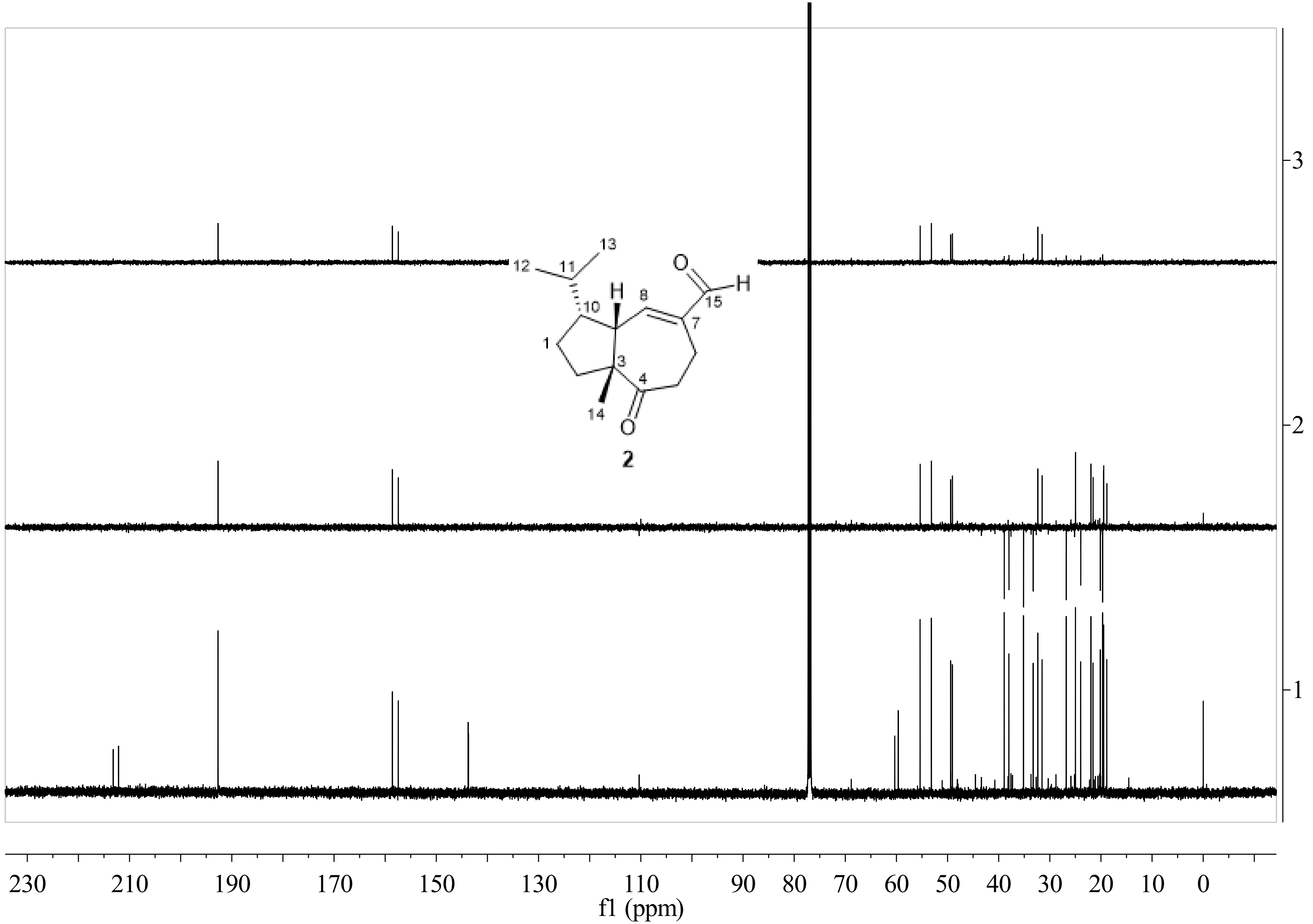

Figure SS20 DEPT spectrum of sinulin A (2) and compound (6)

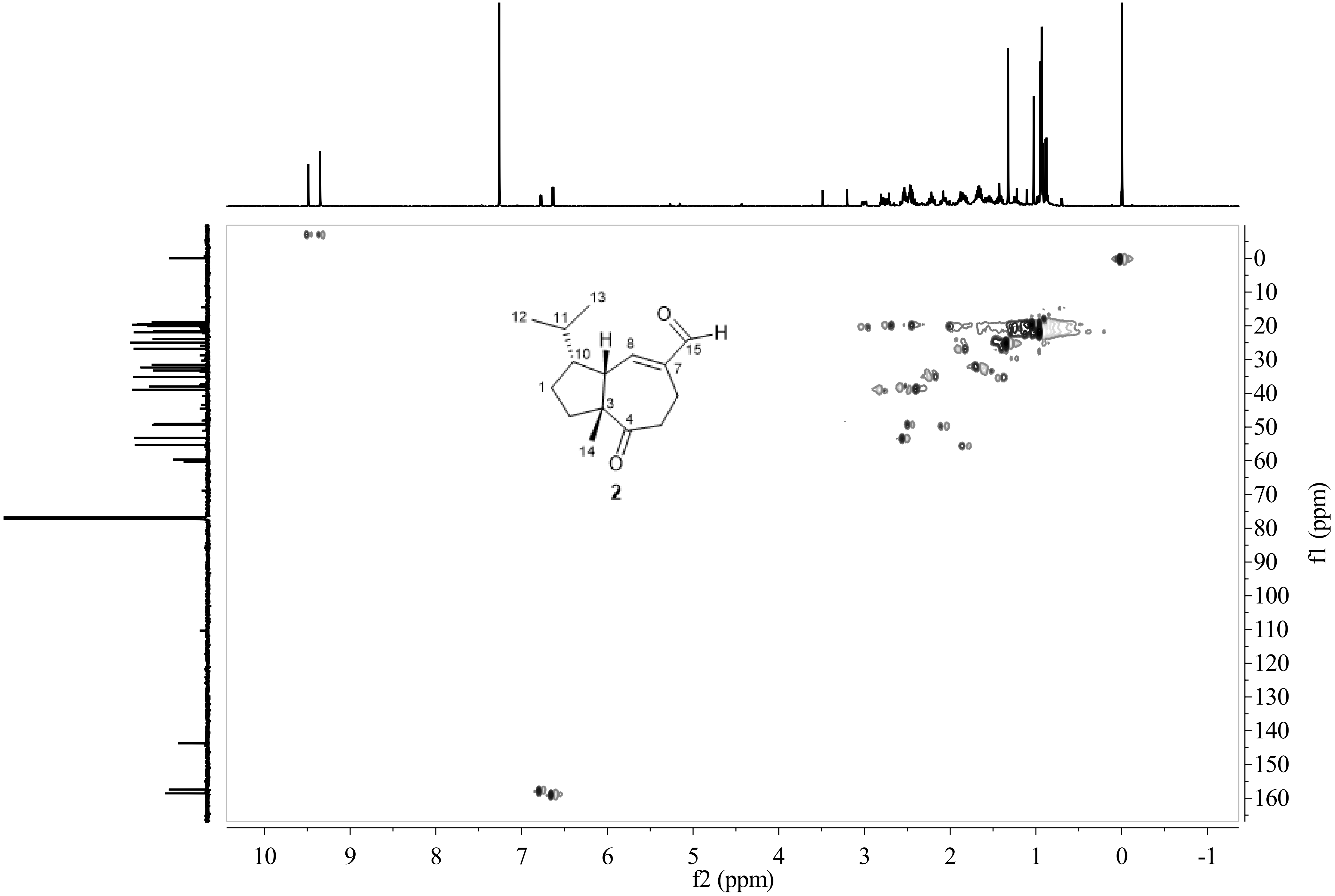

Figure SS21 HMQC spectrum of sinulin A (2) and compound (6)

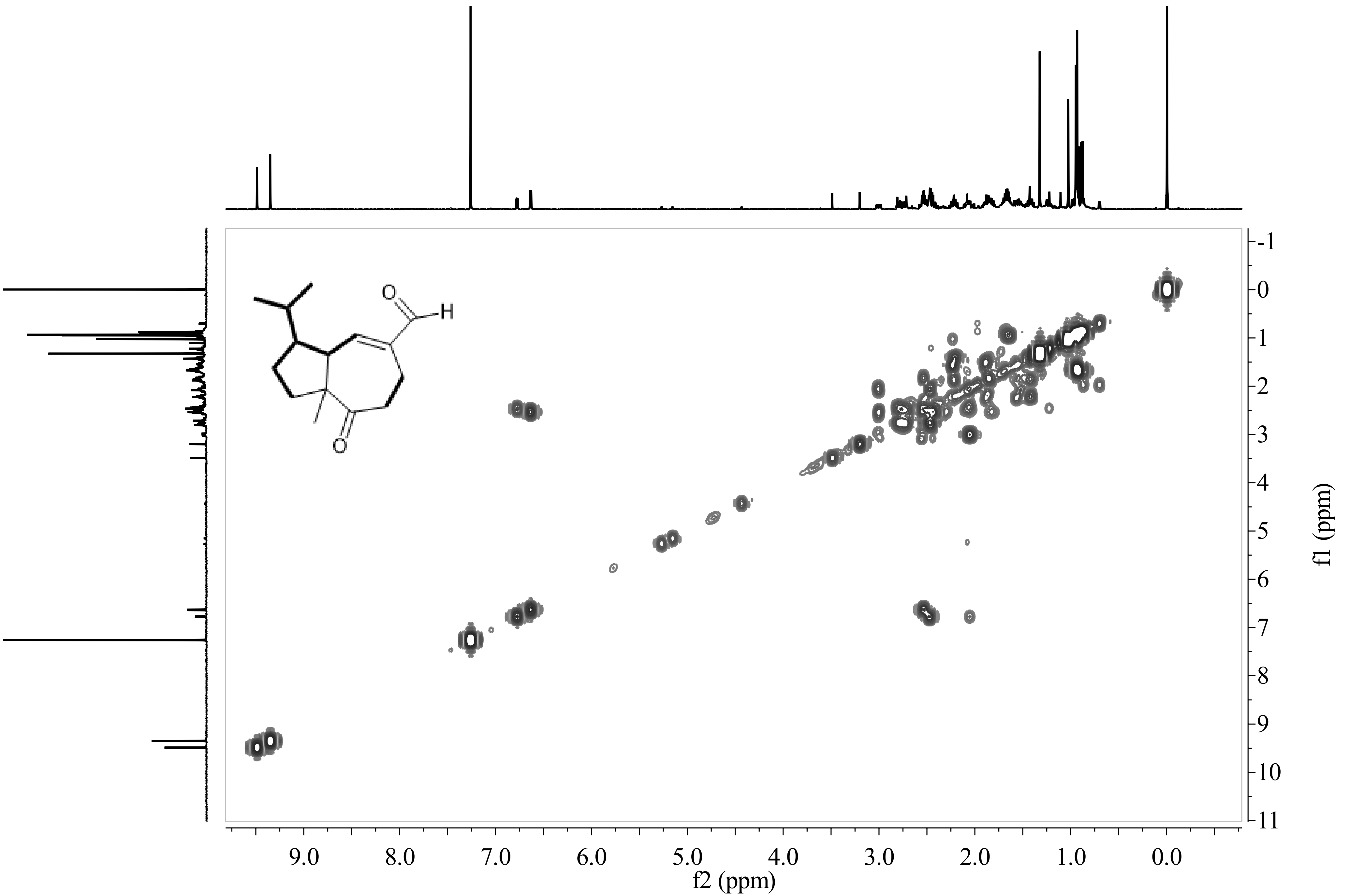

Figure SS22 ^{1}H-^{1}H COSY spectrum of sinulin A (2) and compound (6)

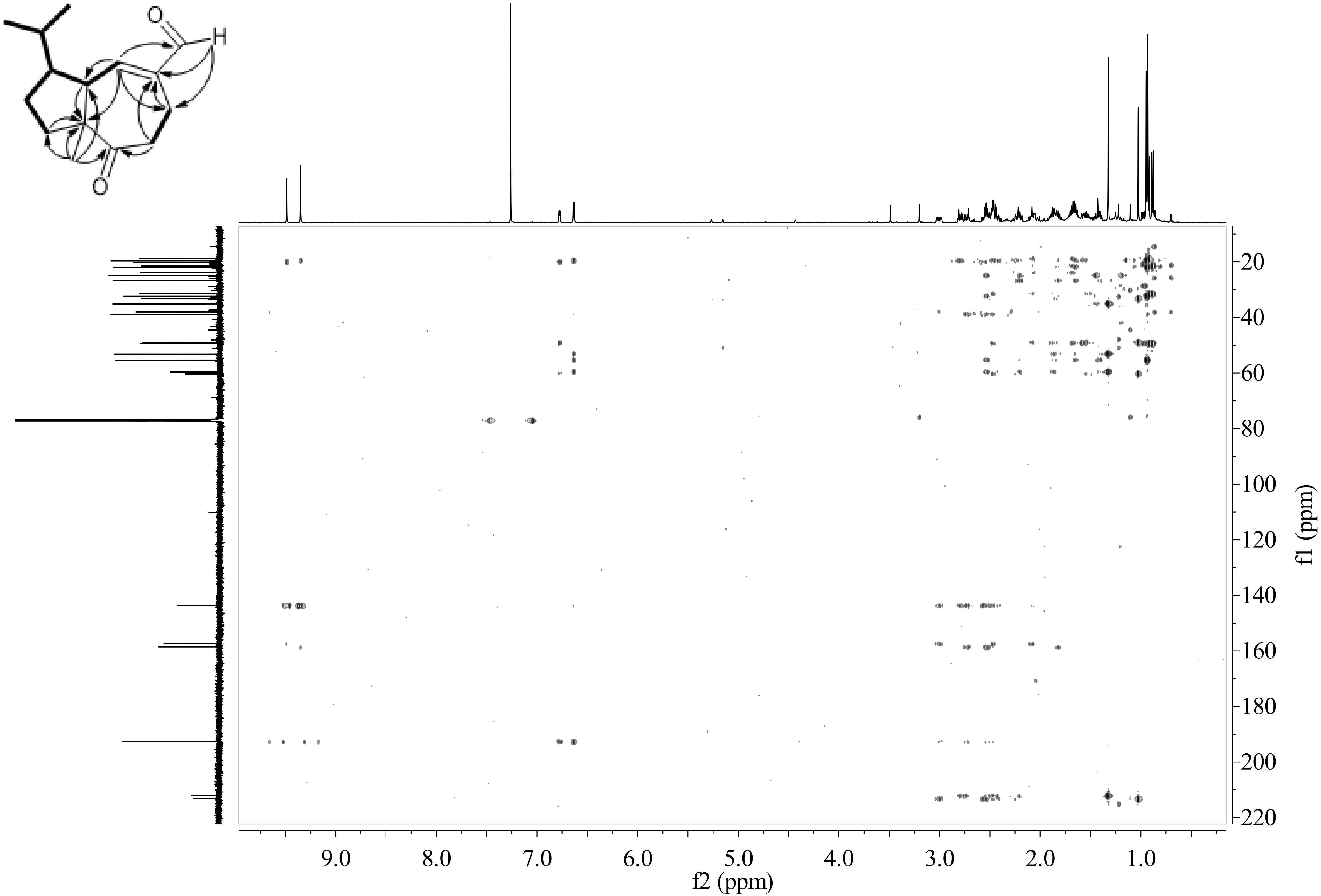

Figure SS23 HMBC spectrum of sinulin A (2) and compound (6)

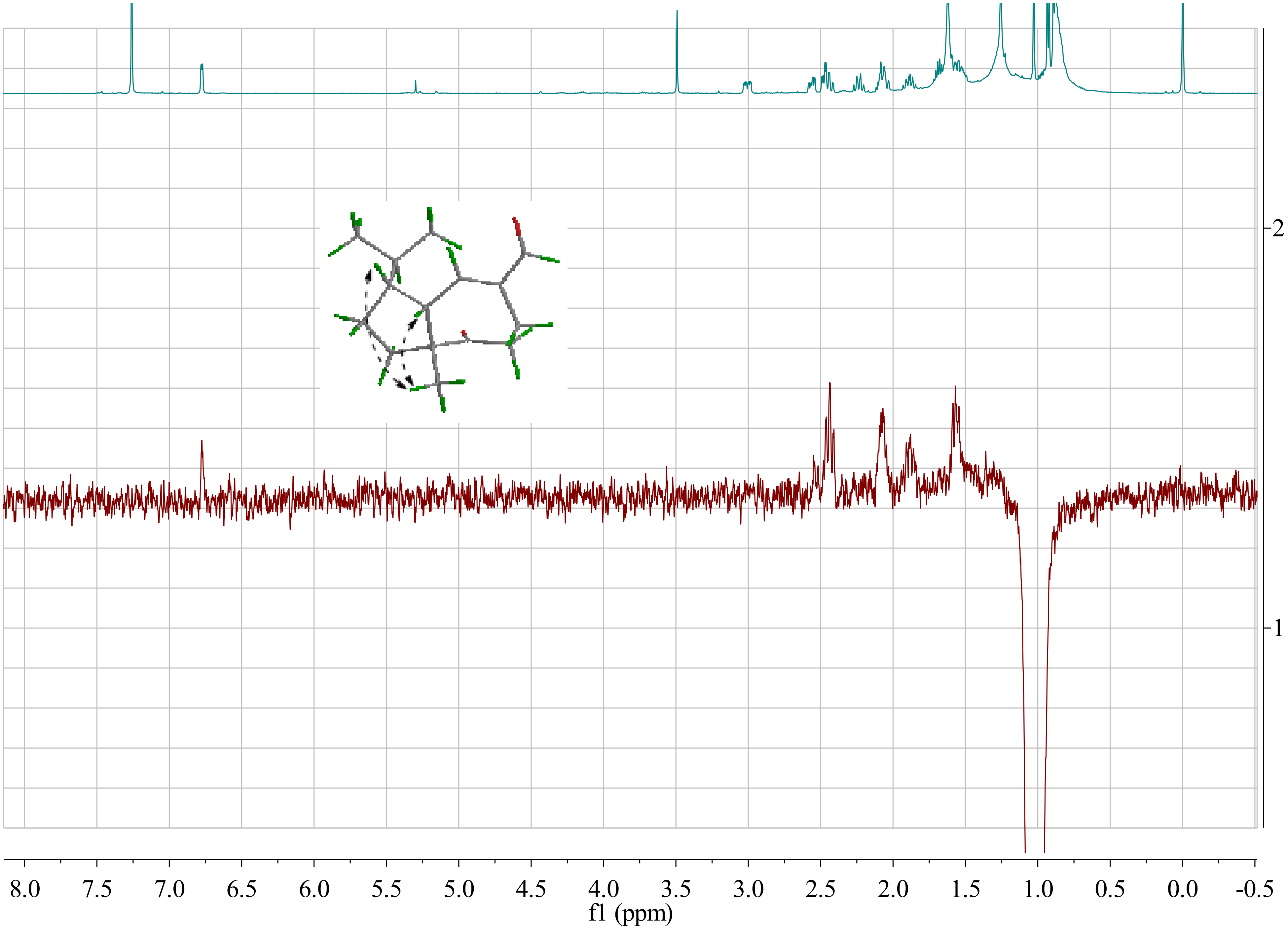

Figure SS24 NOE spectrum of sinulin A (2)

20160527-F5-5-2-2-2_160524155154 5/27/2016 9:05:01 AM F5-5-2-2-2

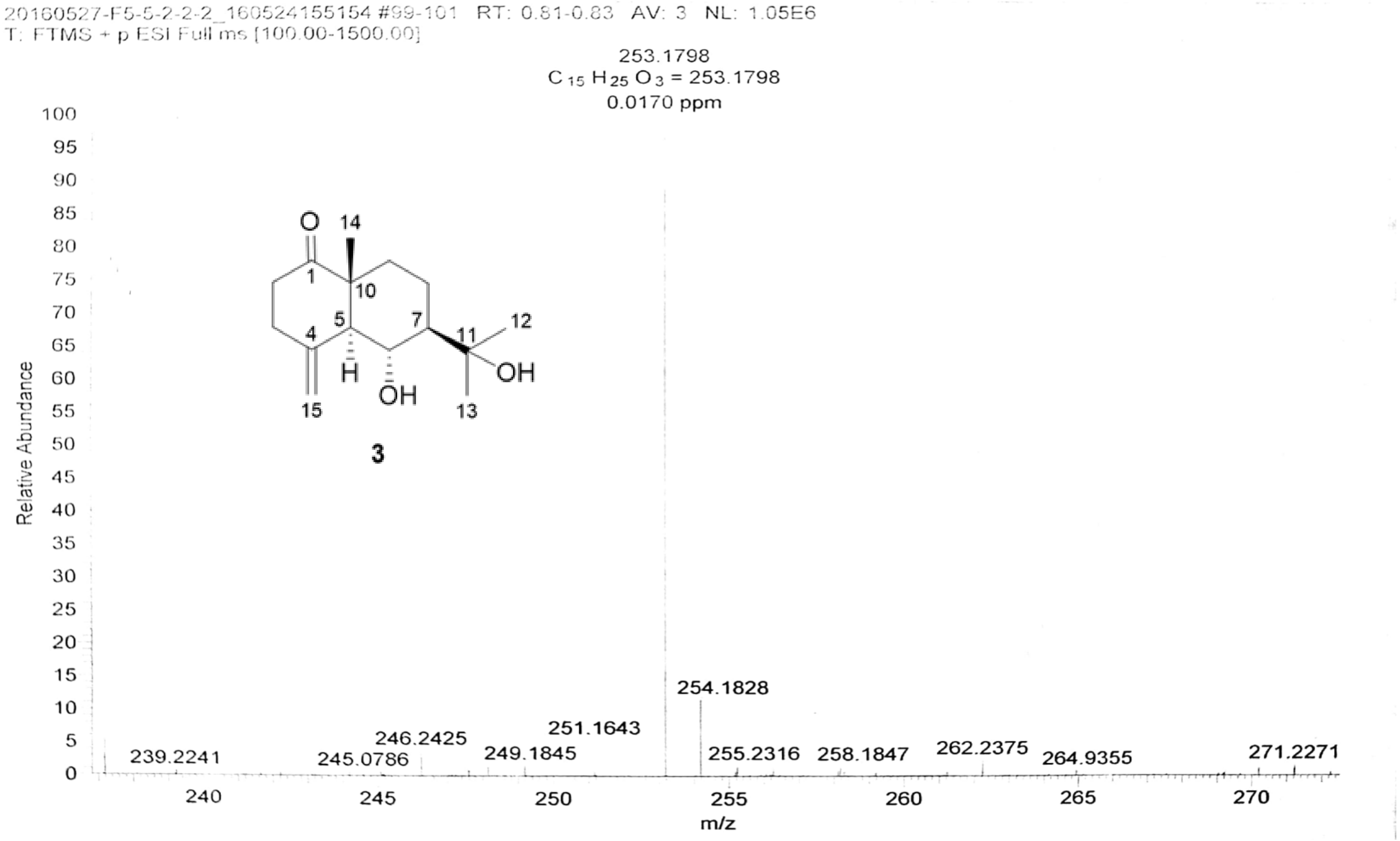

Figure SS25 The positive HRESIMS spectrum of sinulin B (3)

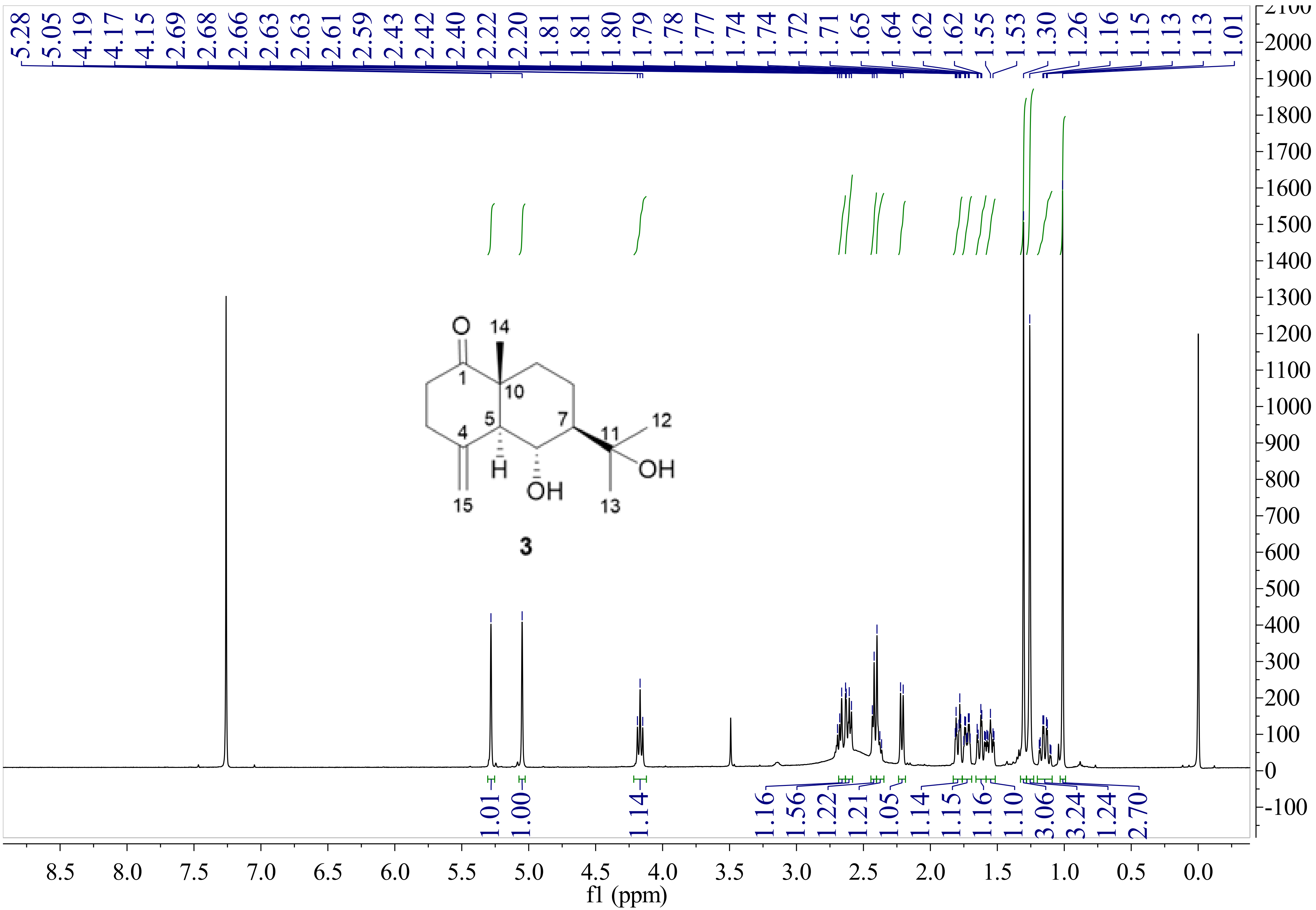

Figure SS26 ^{1}H NMR (500MHz, CDCl$_3$) spectrum of sinulin B (3)

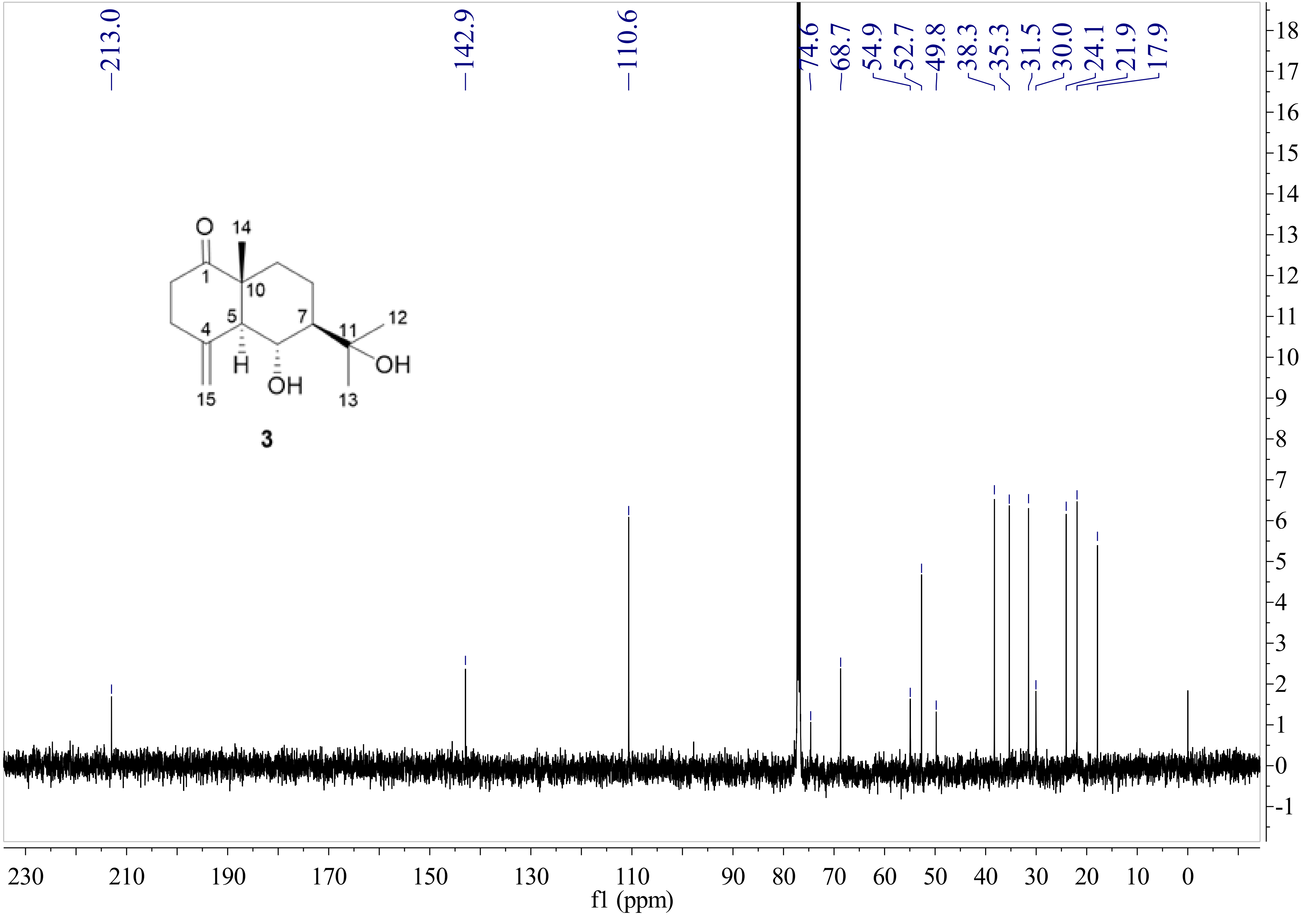

Figure SS27 ^{13}C NMR (125MHz, $CDCl_3$) spectrum of sinulin B (3)

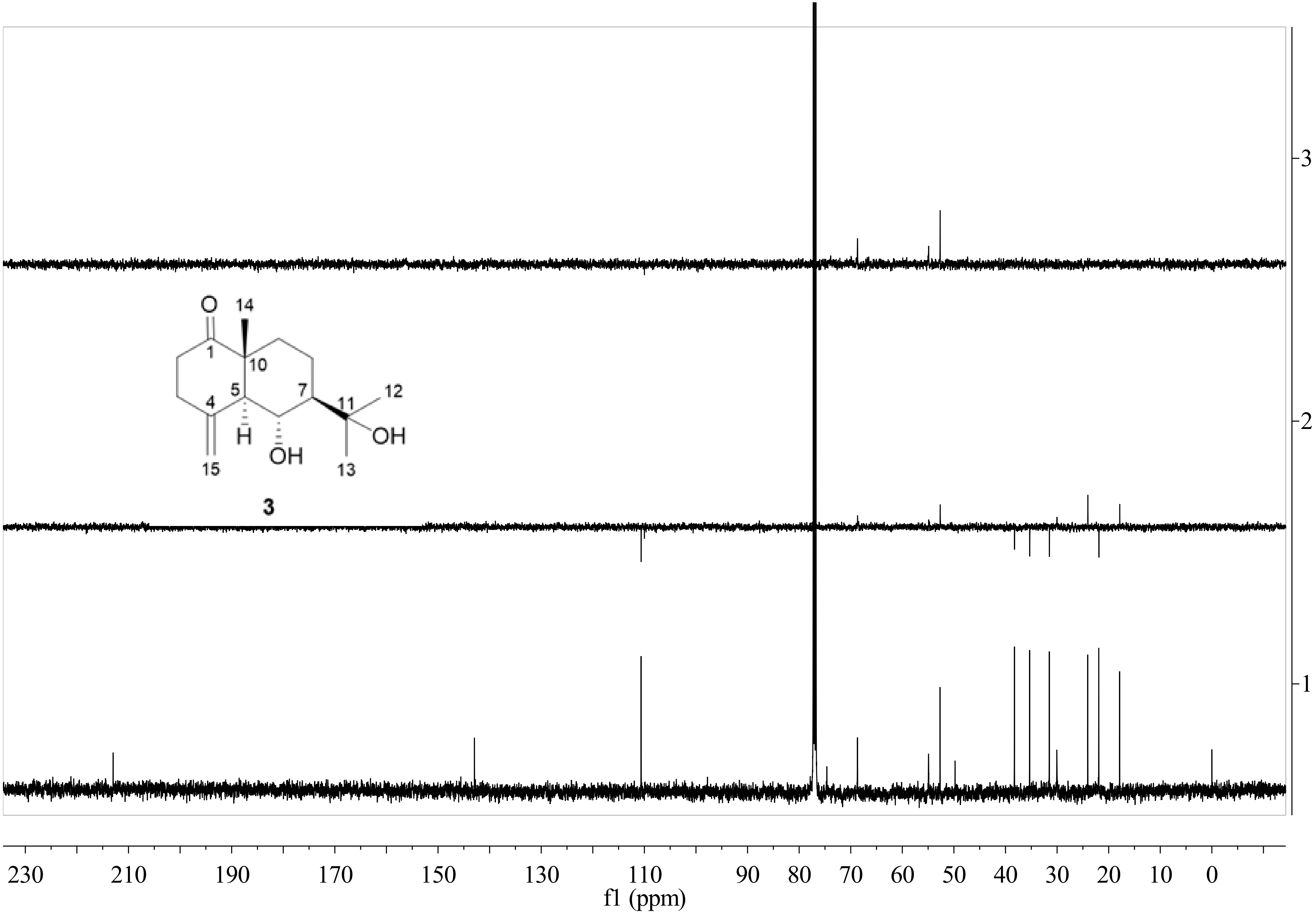

Figure SS28 DEPT spectrum of sinulin B (3)

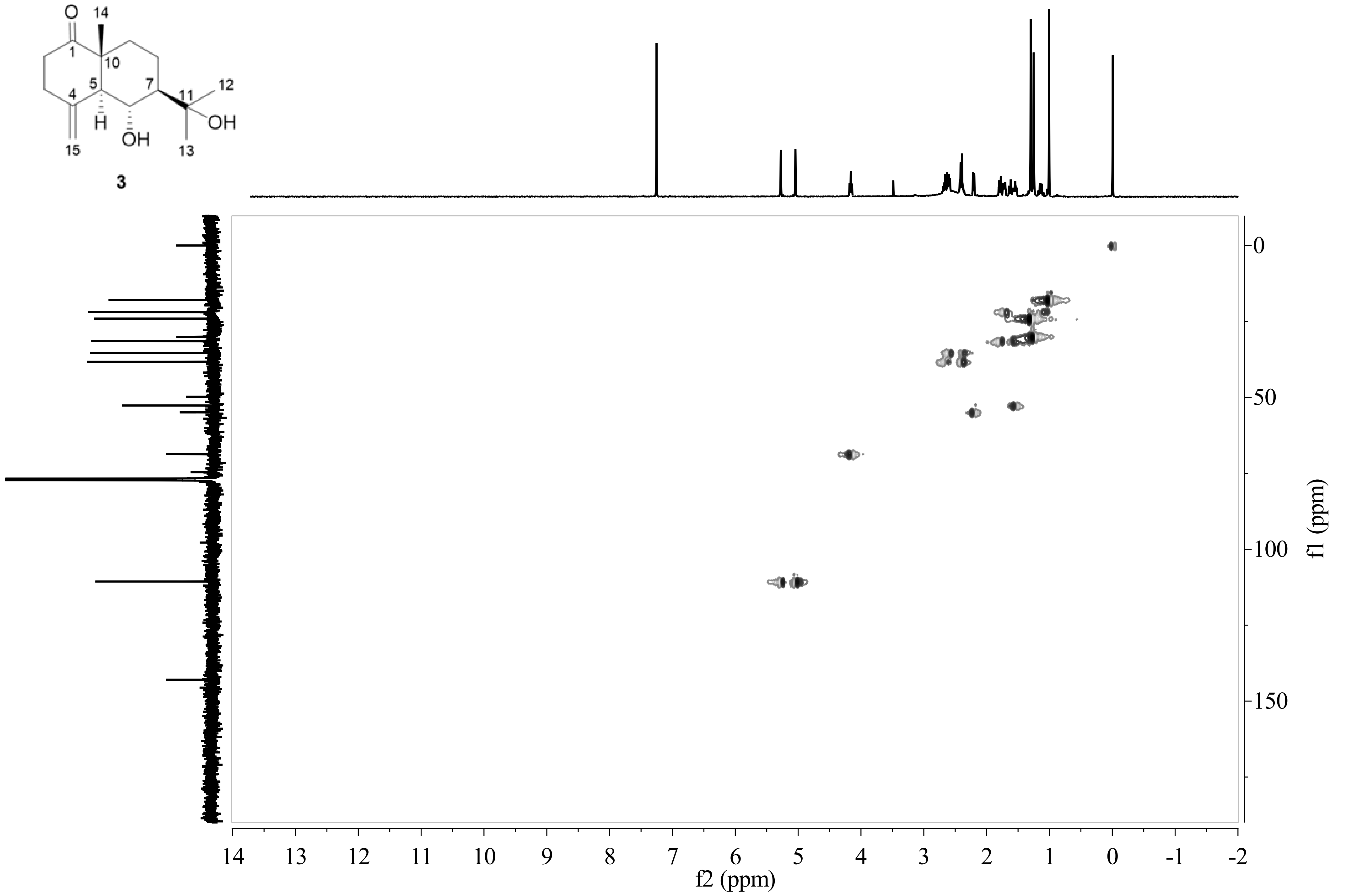

Figure SS29 HMQC spectrum of sinulin B (3)

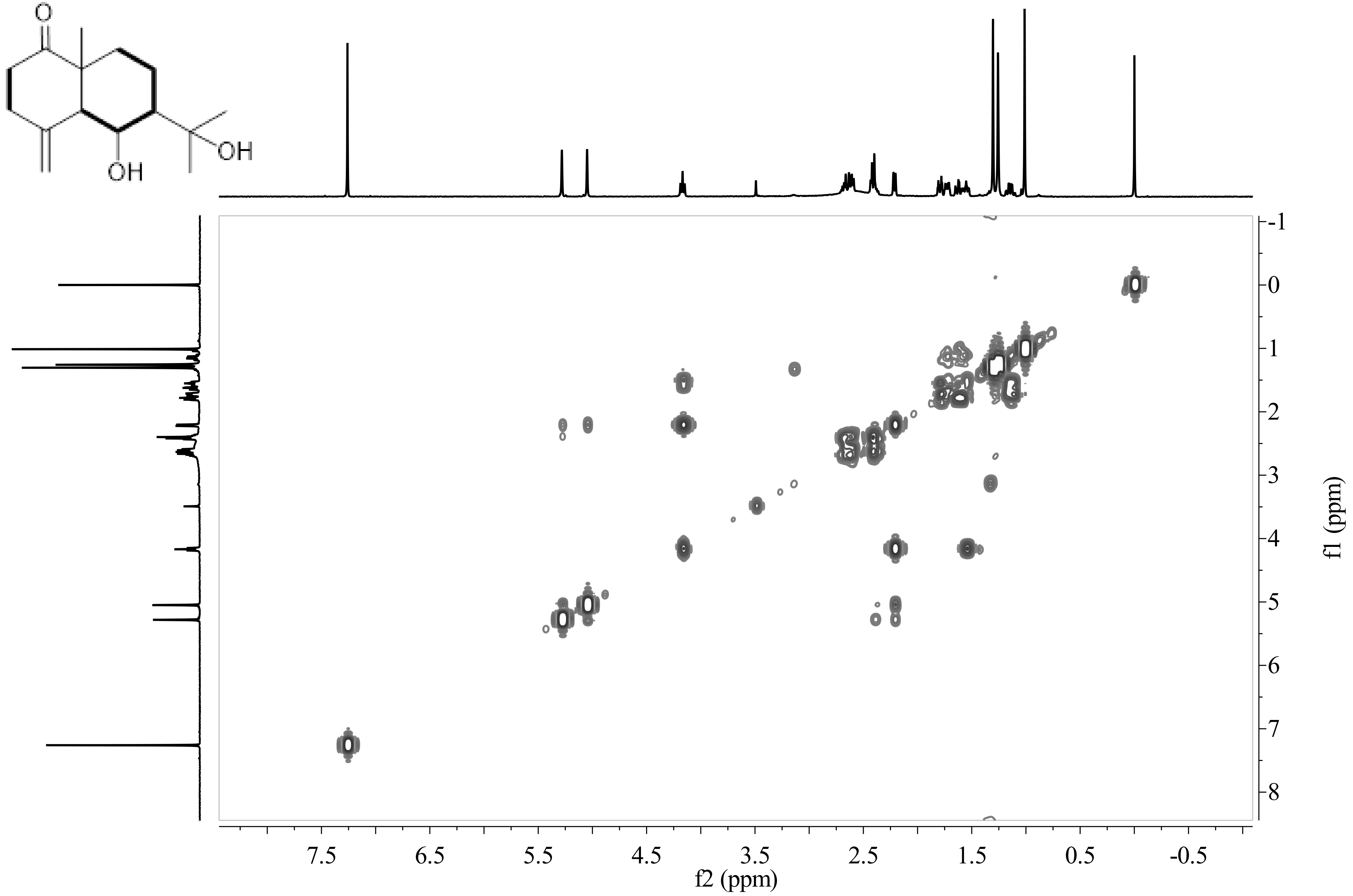

Figure SS30 ¹H-¹H COSY spectrum of sinulin B (3)

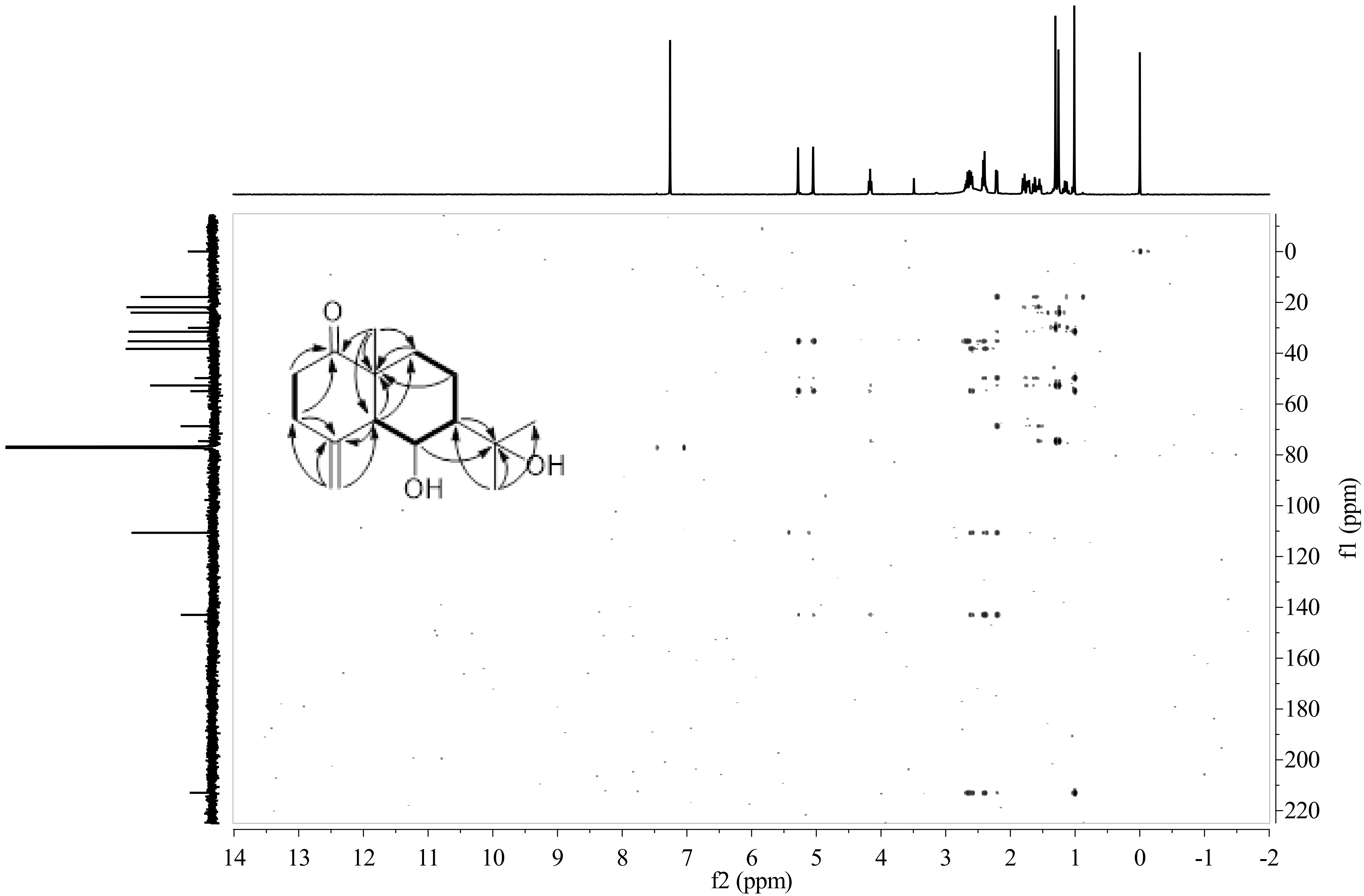

Figure SS31 HMBC spectrum of sinulin B (3)

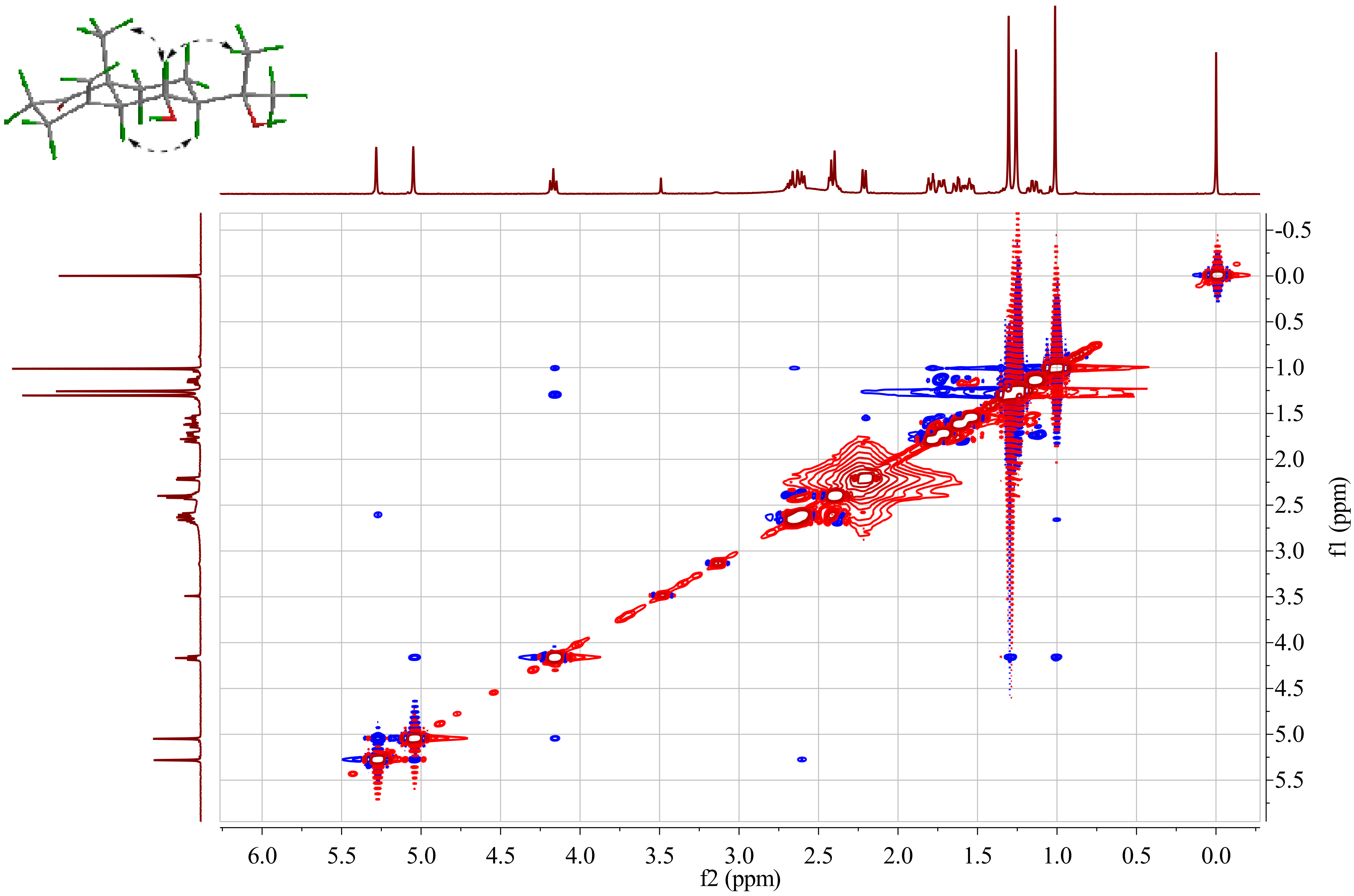

Figure SS32 NOESY spectrum of sinulin B (3)

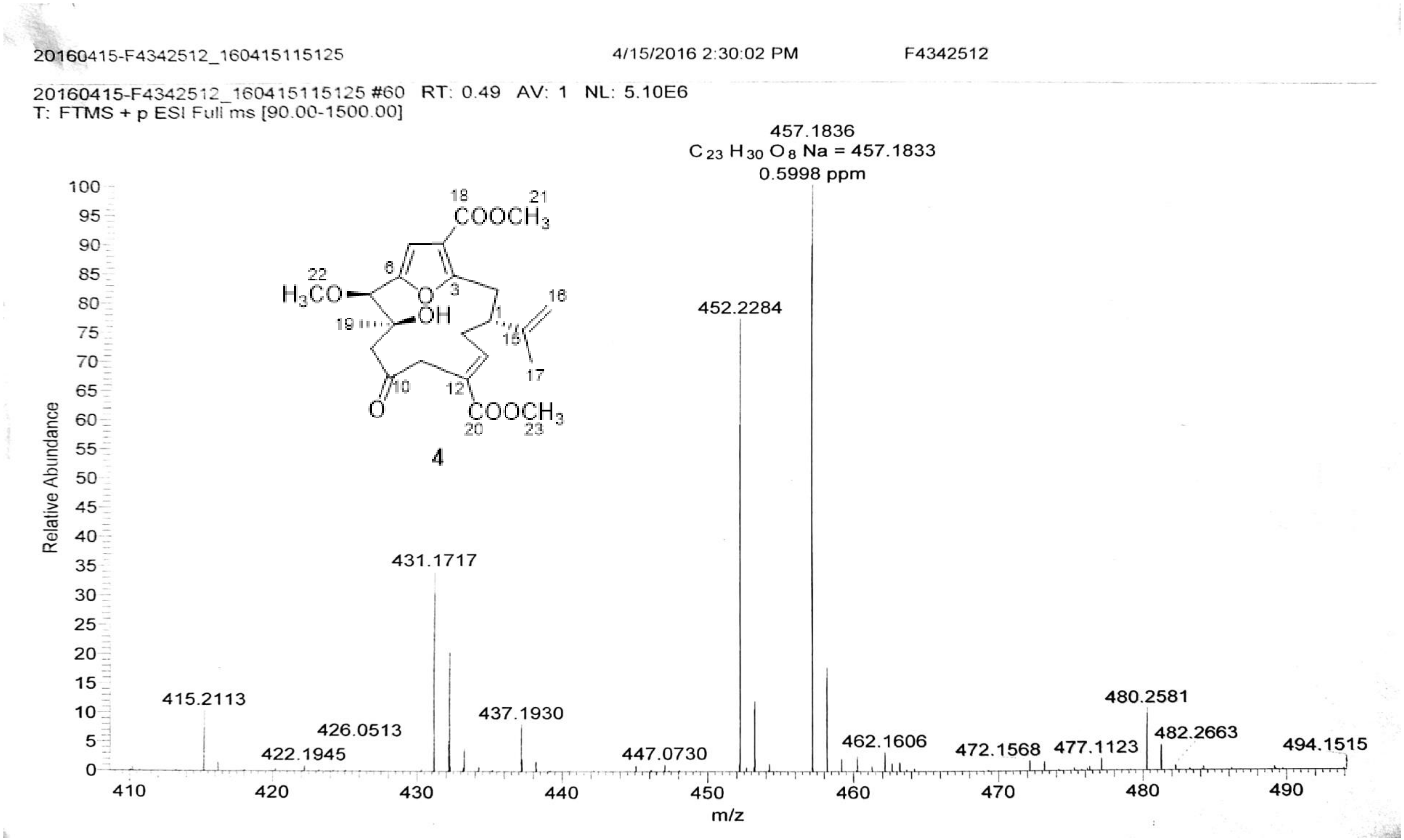

Figure SS33 The positive HRESIMS spectrum of sinulin C (4)

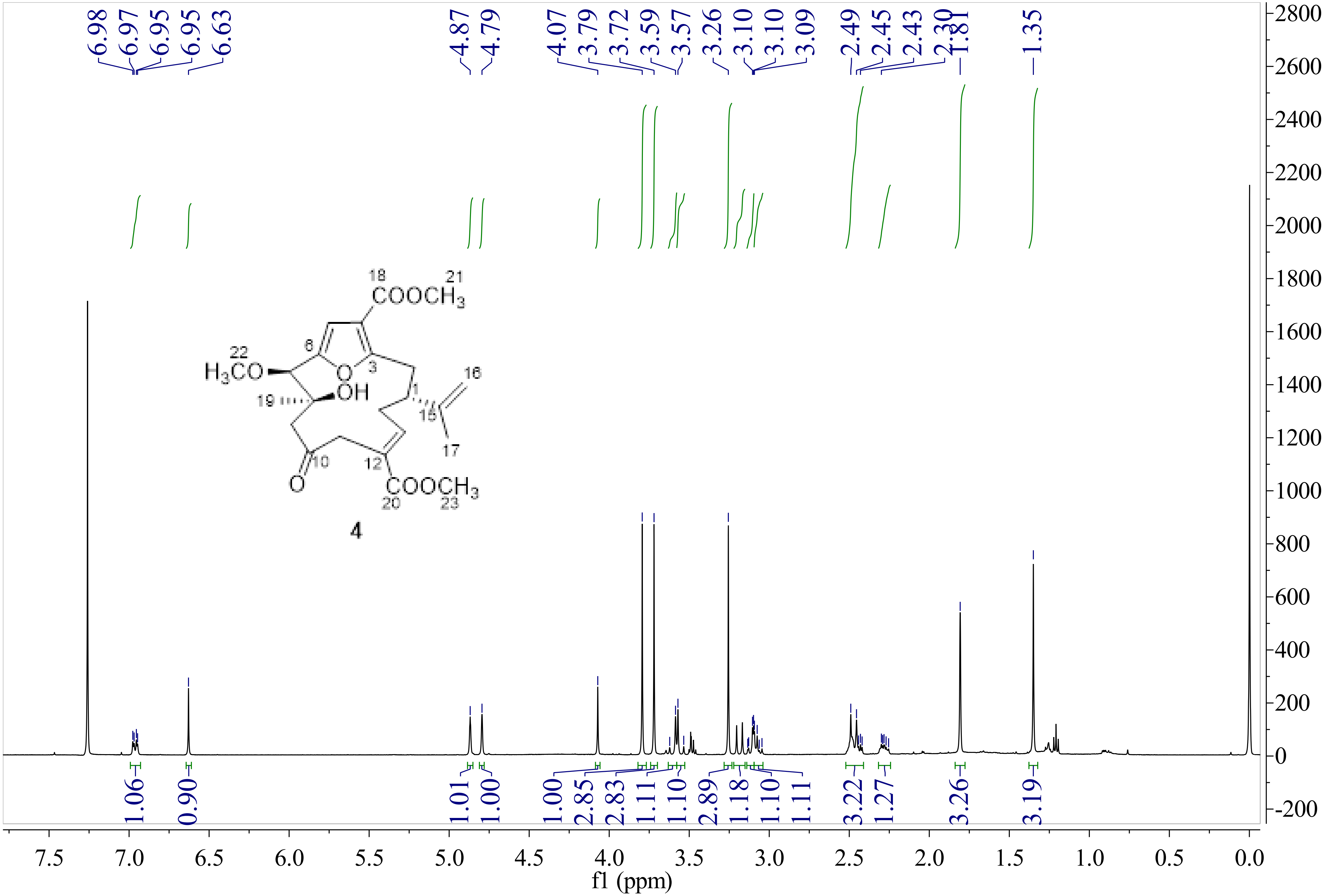

Figure SS34 ^{1}H NMR (500MHz, $CDCl_3$) spectrum of sinulin C (4)

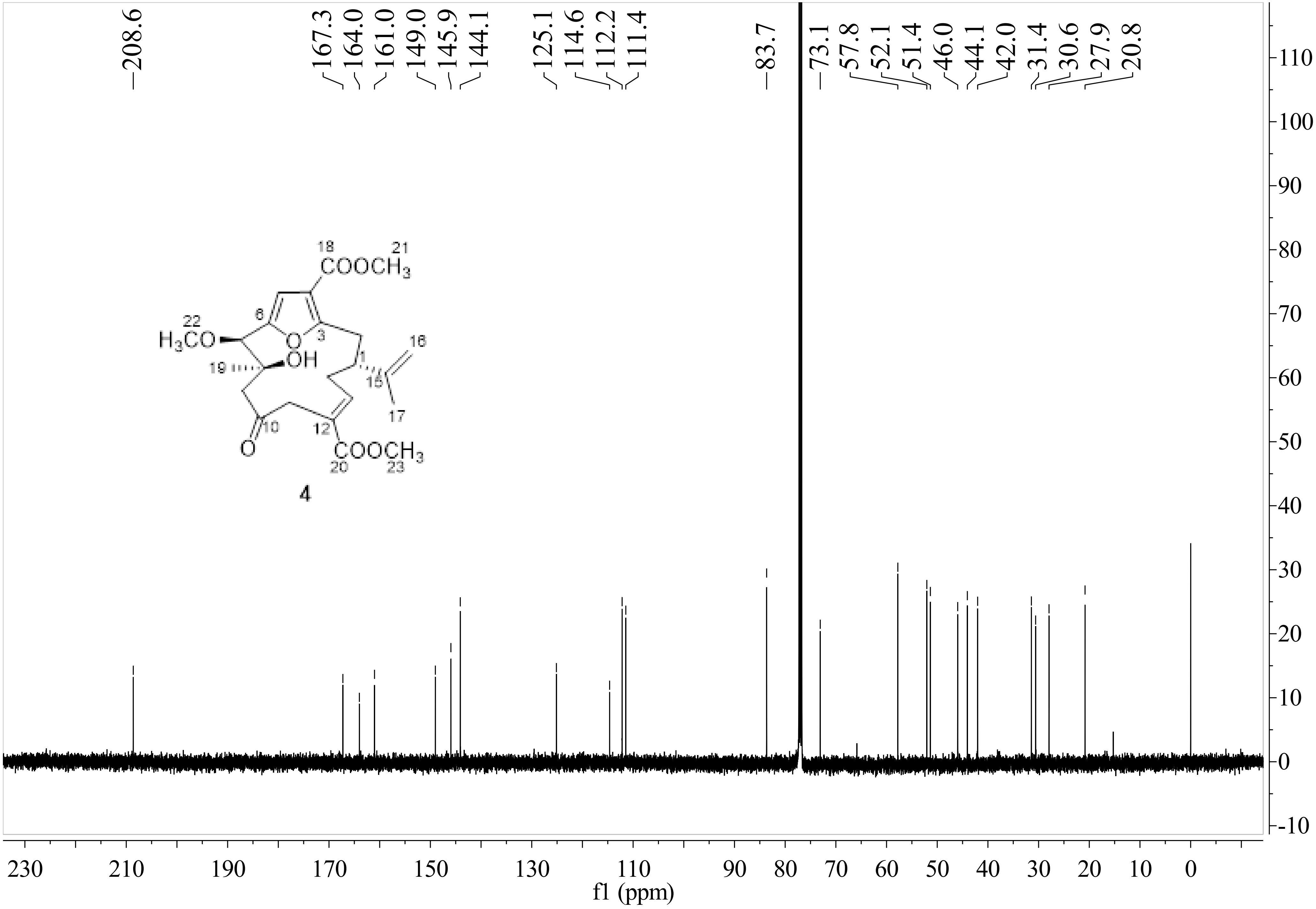

Figure SS35 ^{13}C NMR (125MHz, $CDCl_3$) spectrum of sinulin C (4)

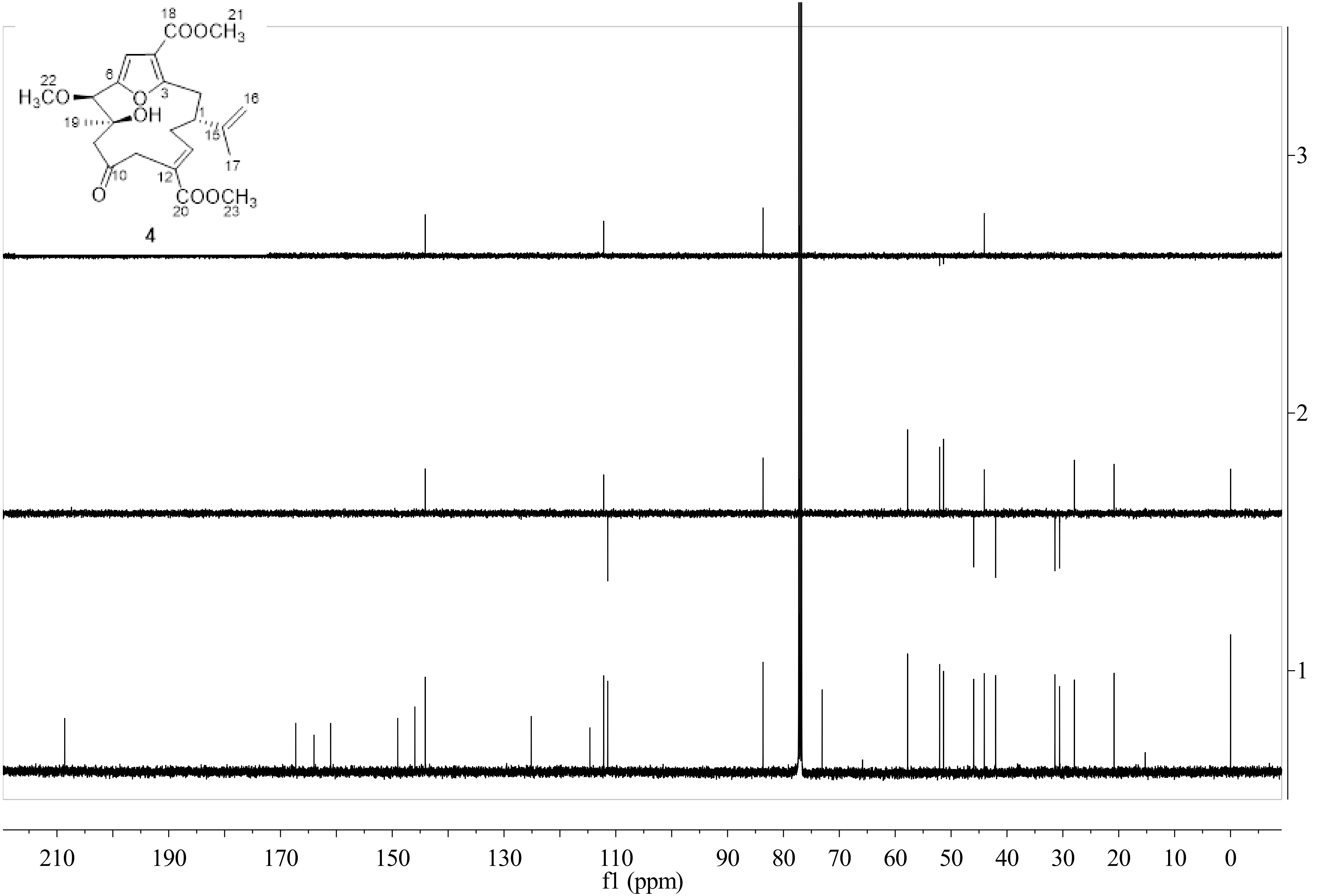

Figure SS36 DEPT spectrum of sinulin C (4)

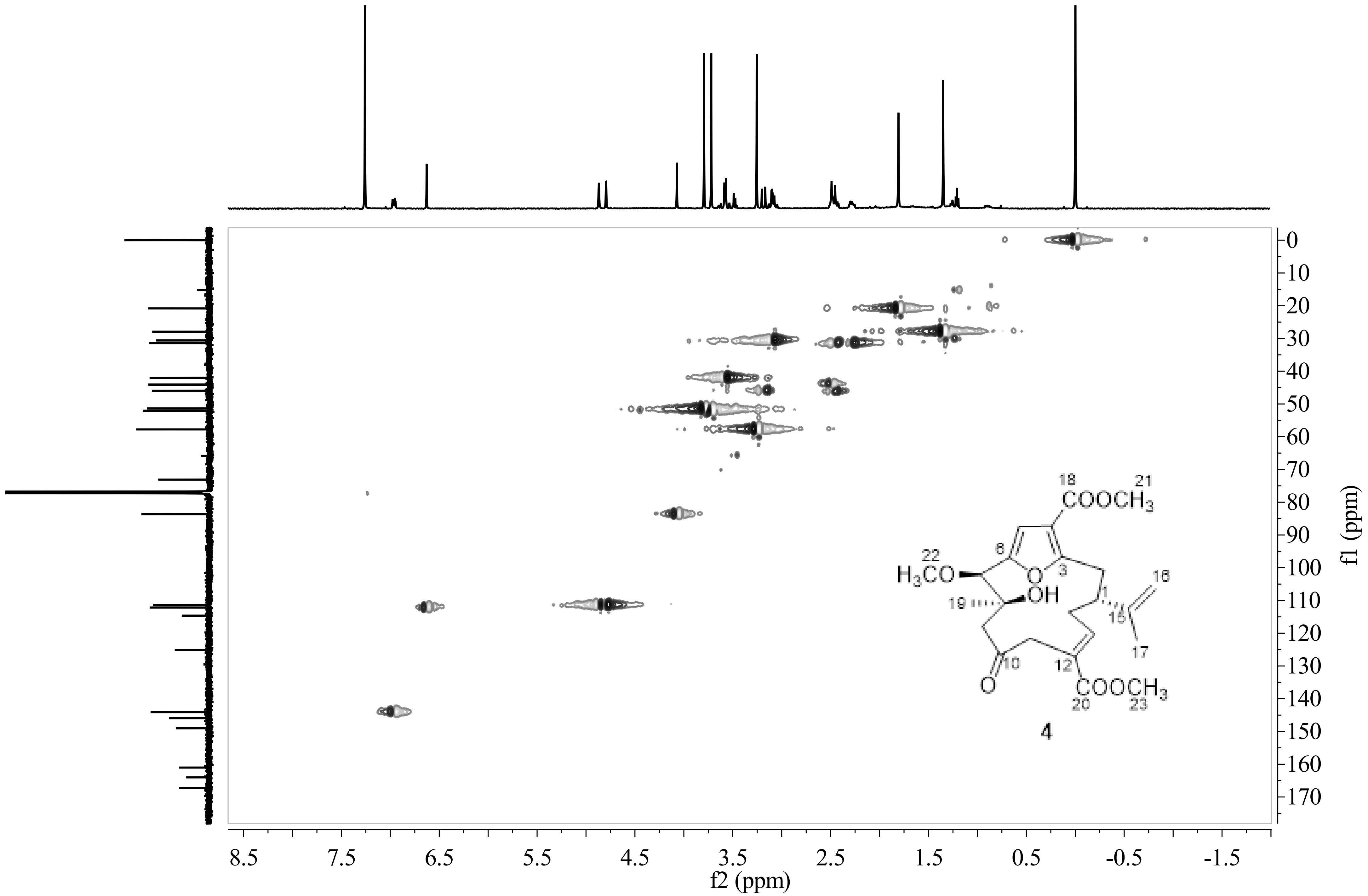

Figure SS37 HMQC spectrum of sinulin C (4)

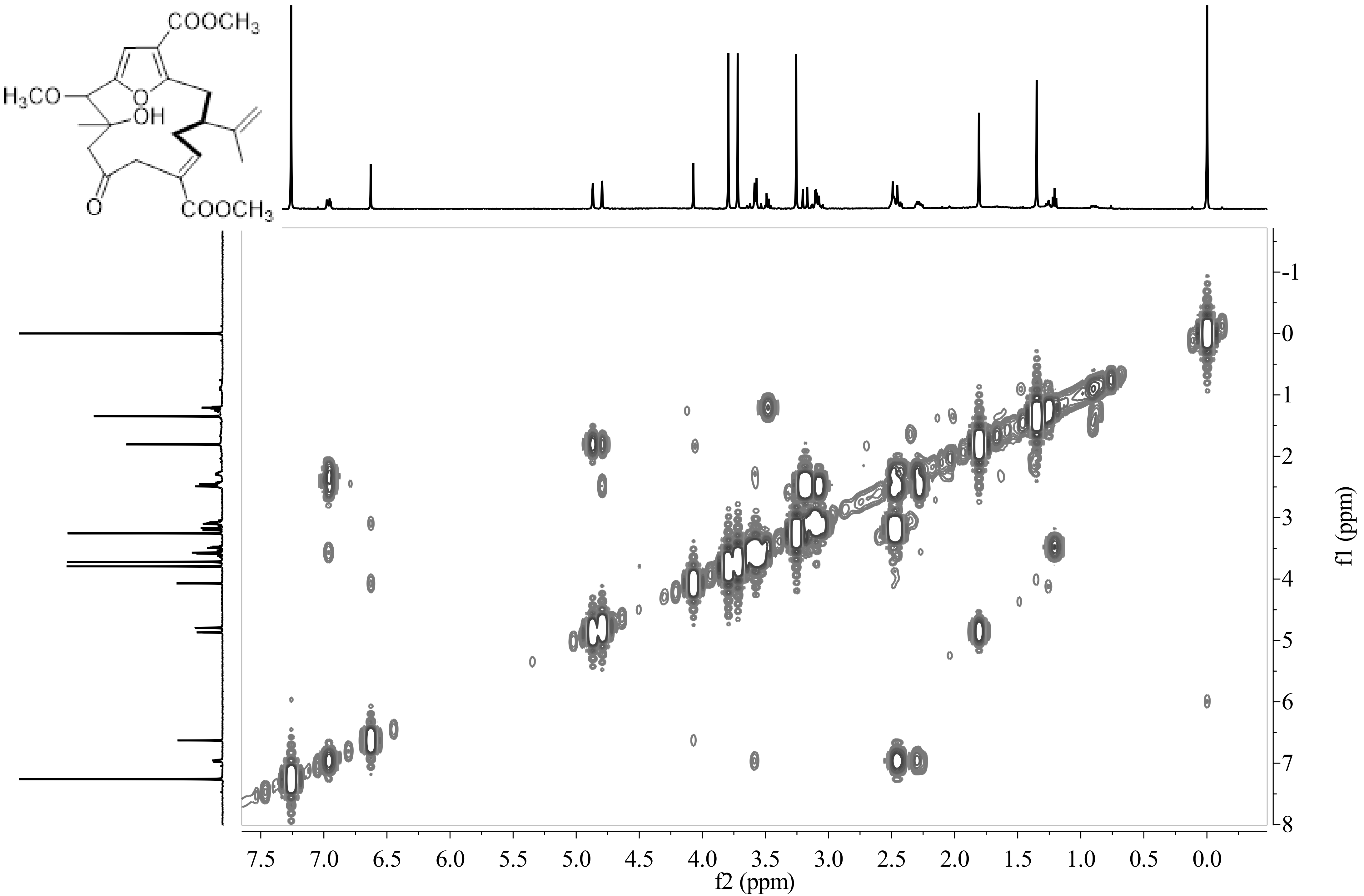

Figure SS38 ^{1}H-^{1}H COSY spectrum of sinulin C (4)

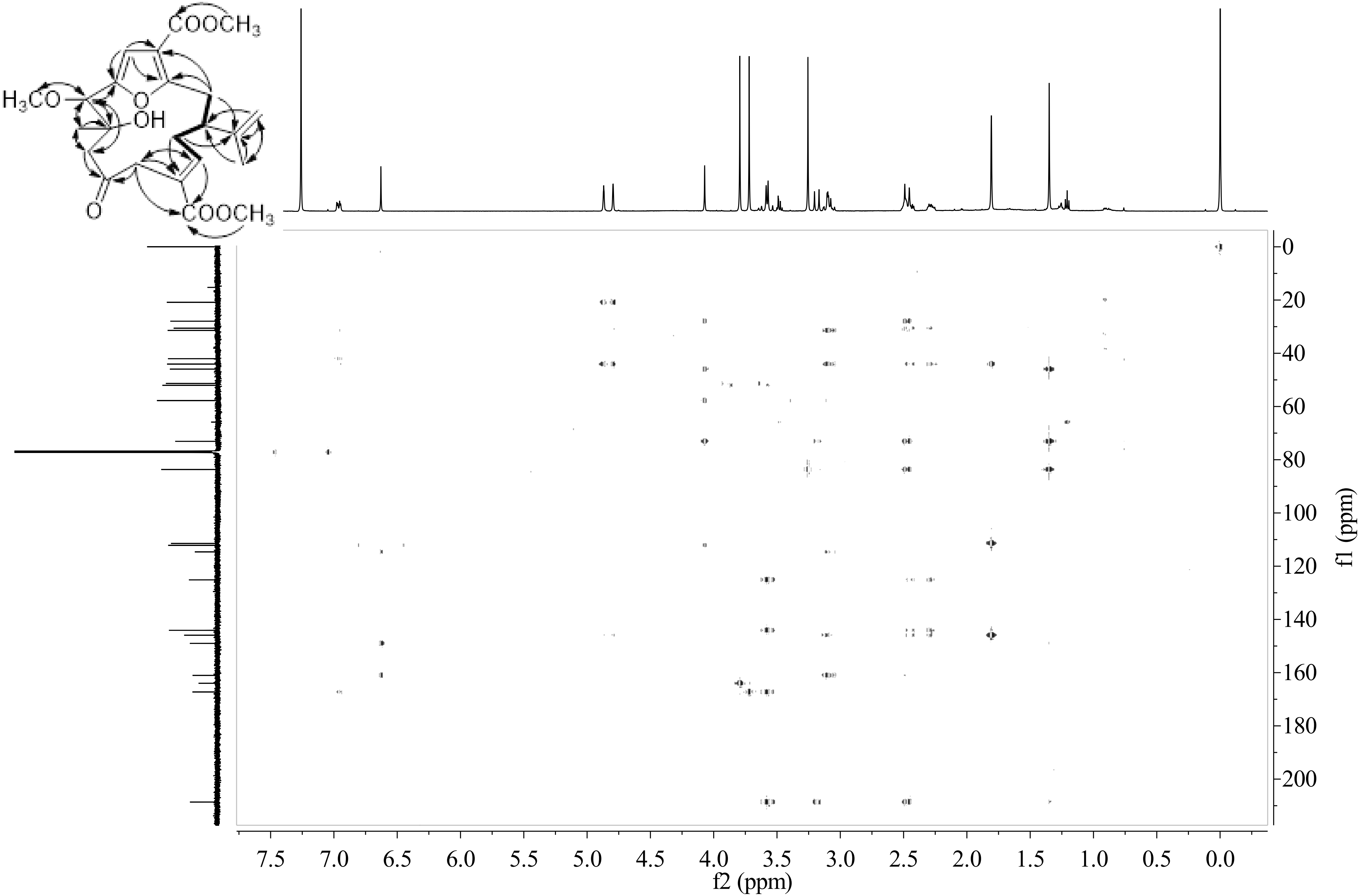

Figure SS39 HMBC spectrum of sinulin C (4)

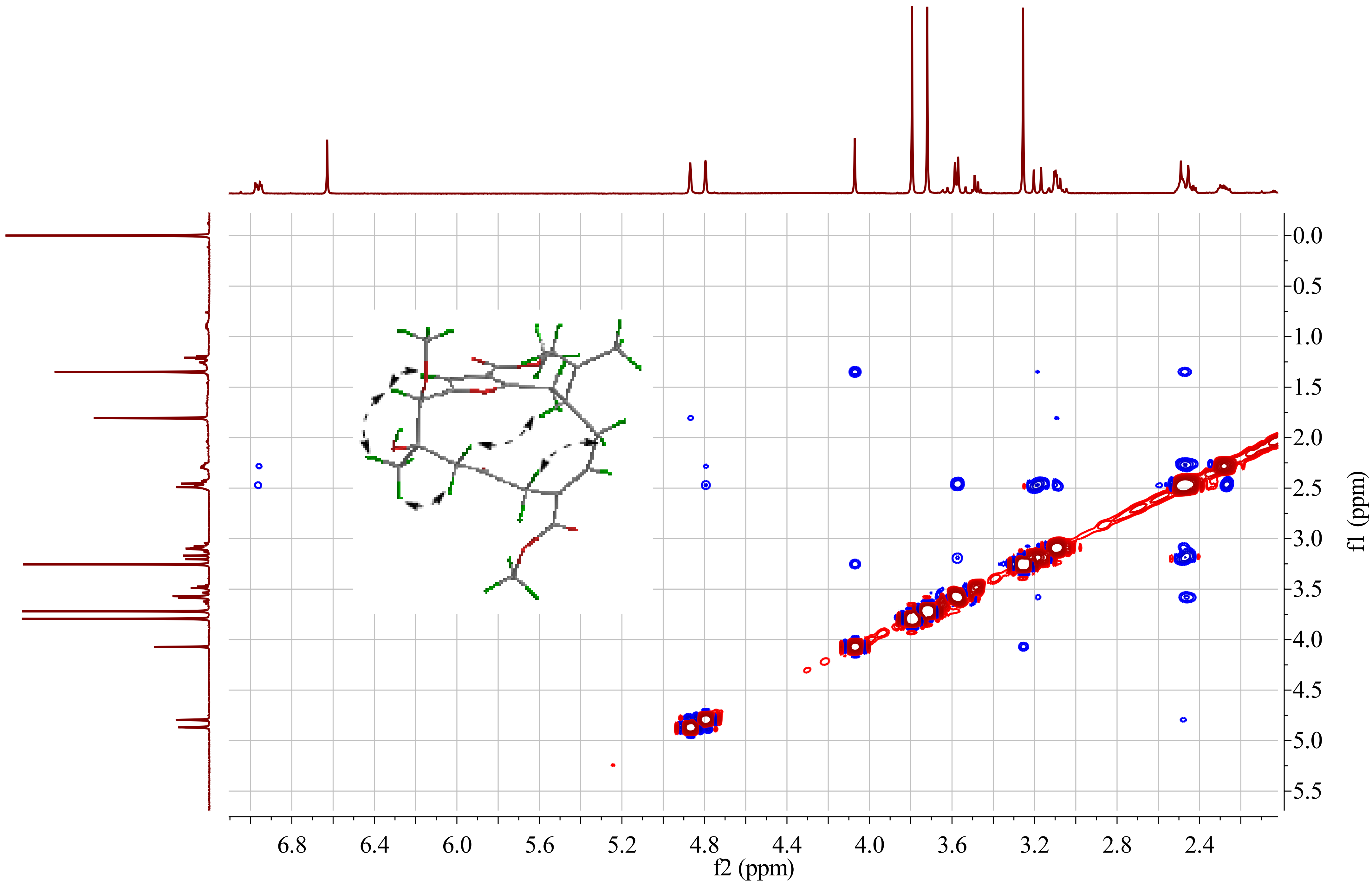

Figure SS40 NOESY spectrum of sinulin C (4)

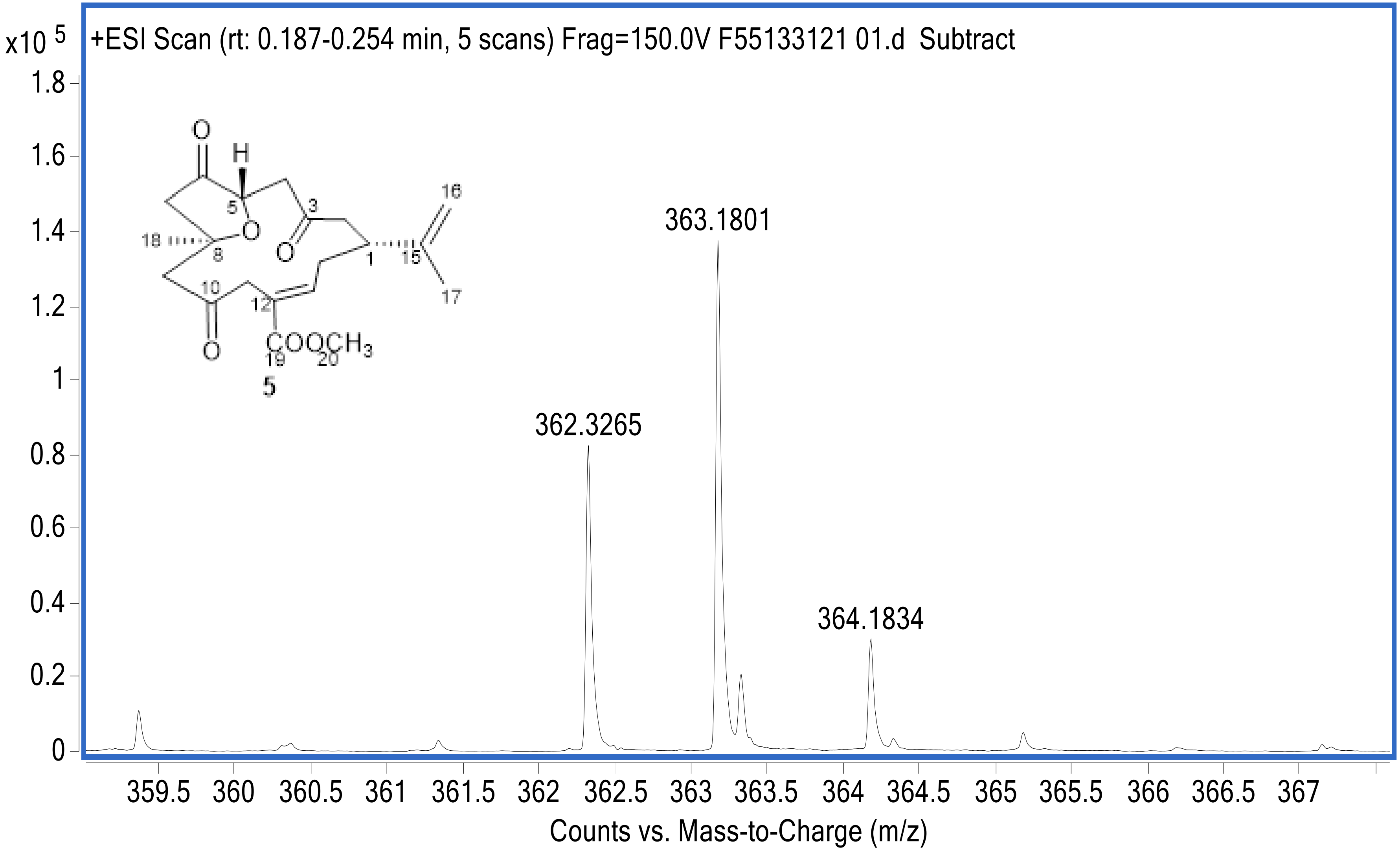

Figure SS41 The positive HRESIMS spectrum of sinulin D (5)

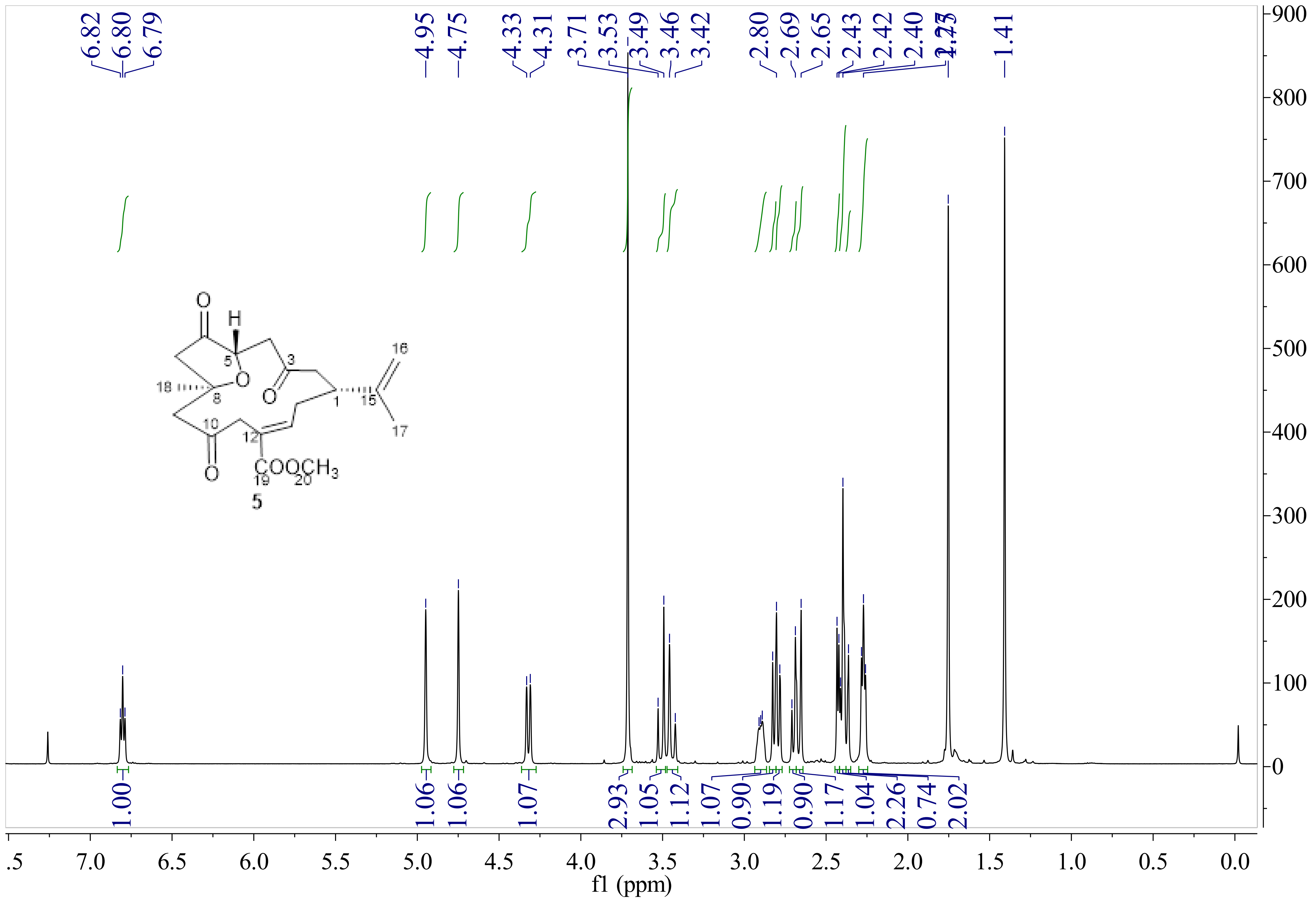

Figure SS42 ^{1}H NMR (500MHz, $CDCl_3$) spectrum of sinulin D (5)

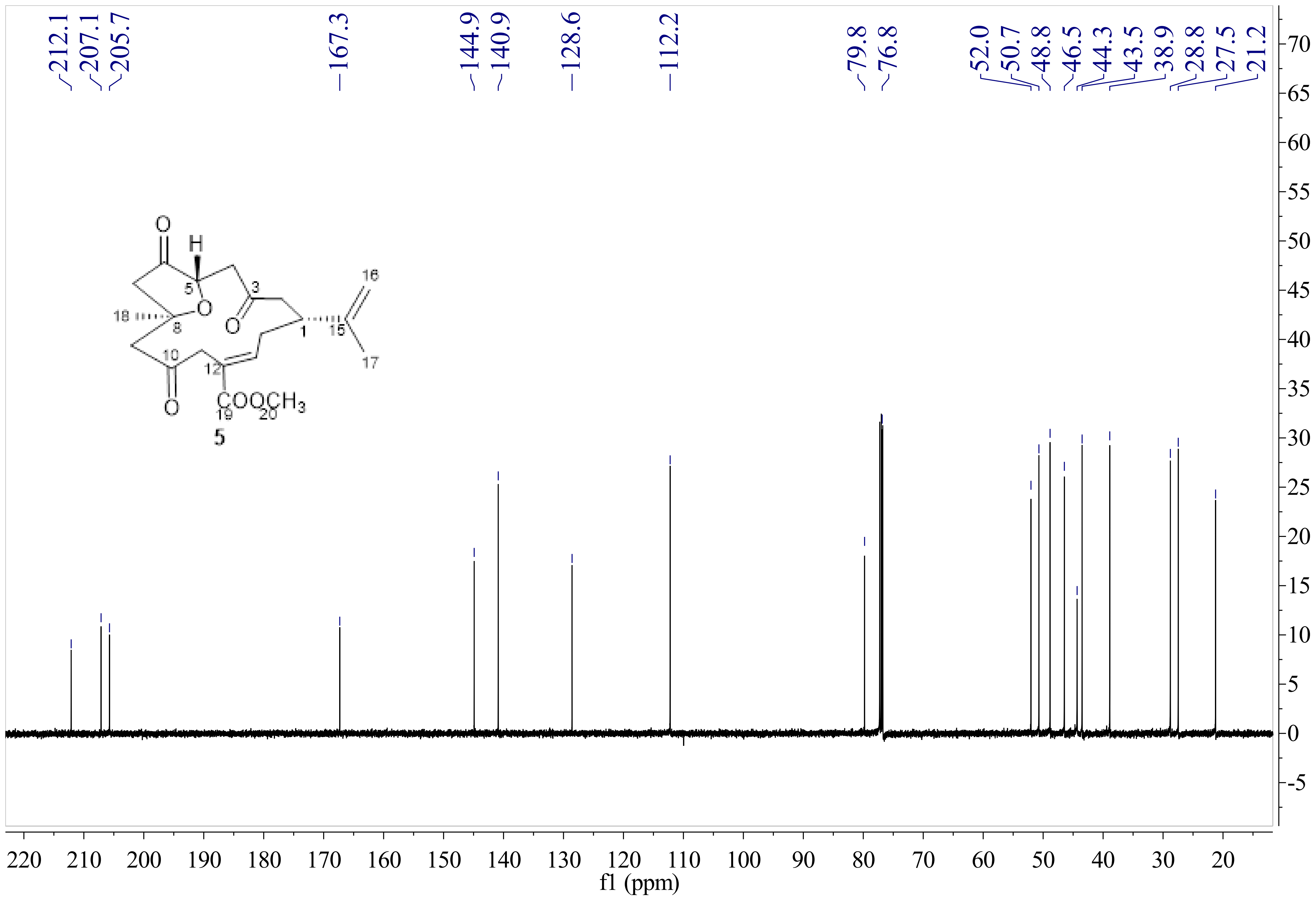

Figure SS43 ^{13}C NMR (125MHz, $CDCl_3$) spectrum of sinulin D (5)

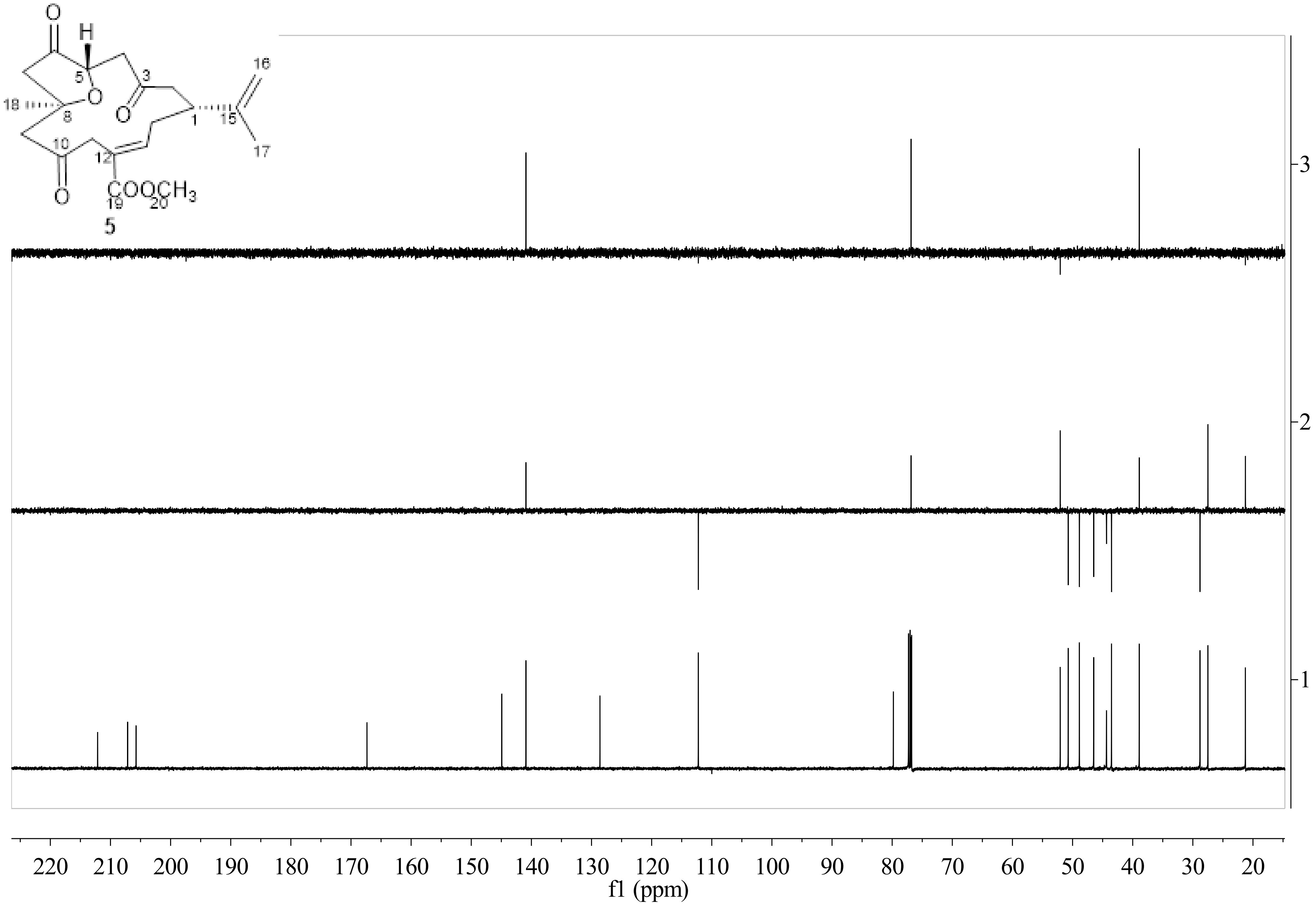

Figure SS44 DEPT spectrum of sinulin D (5)

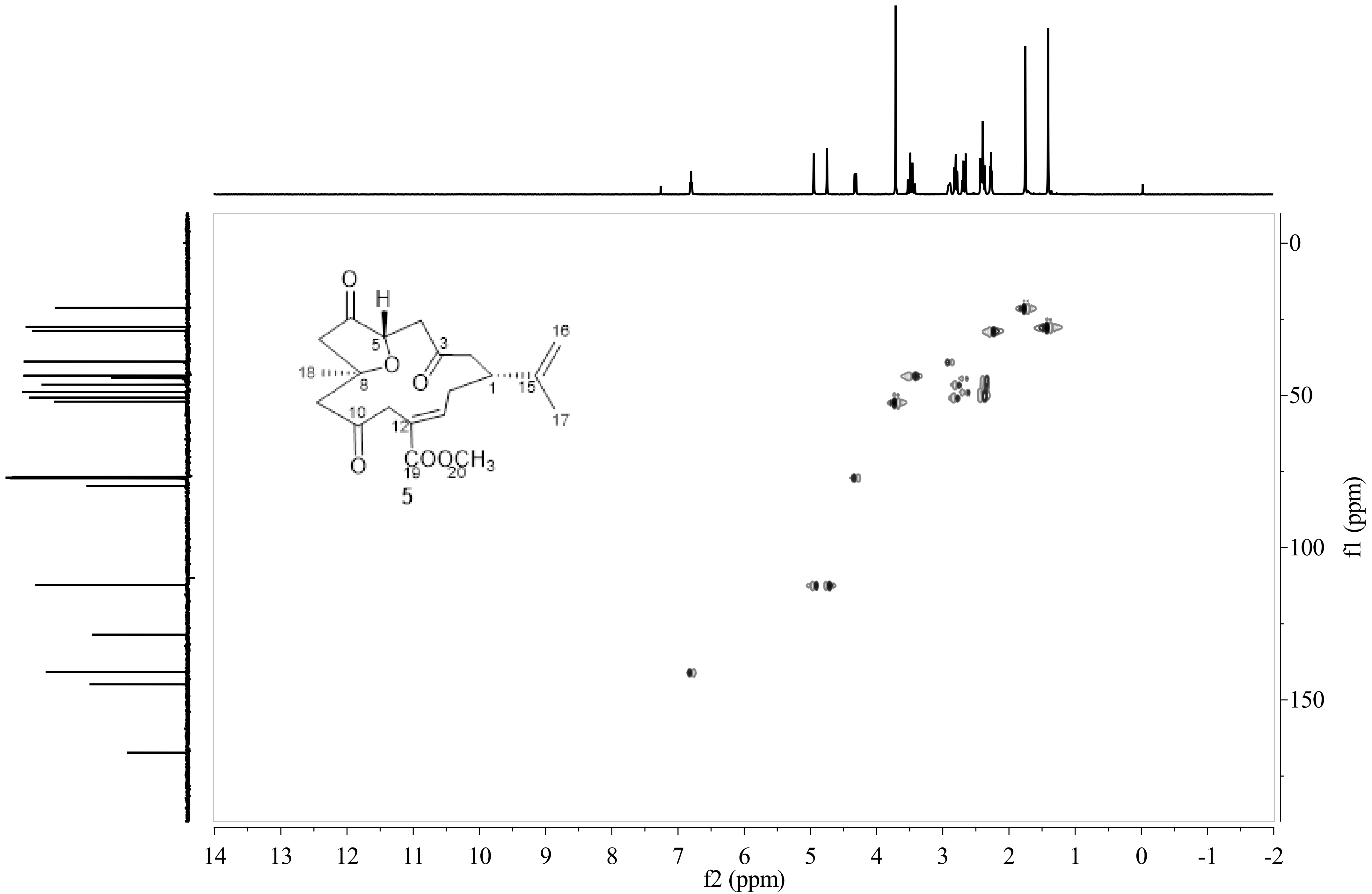

Figure SS45 HMQC spectrum of sinulin D (5)

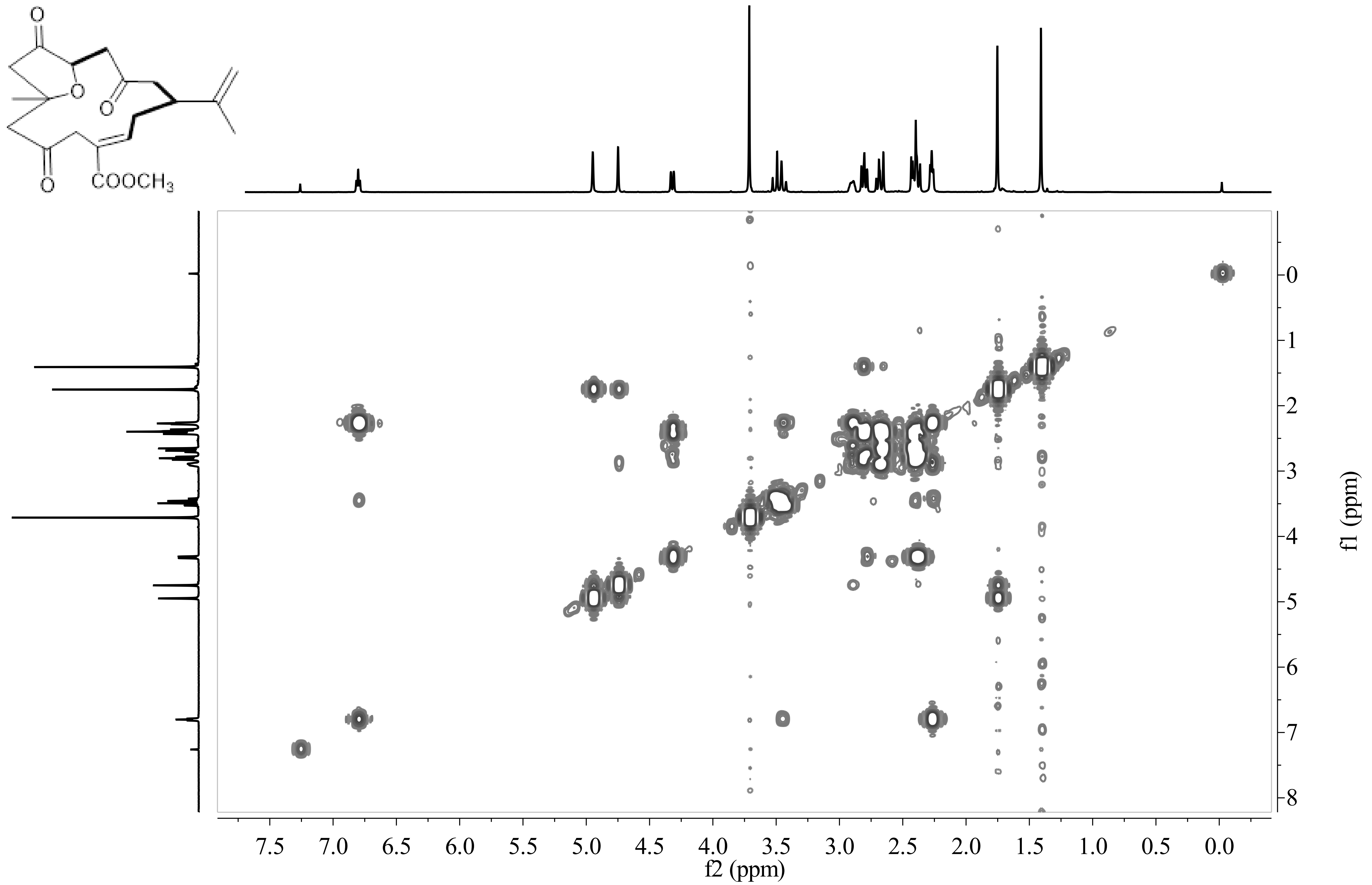

Figure SS46 ^{1}H-^{1}H COSY spectrum of sinulin D (5)

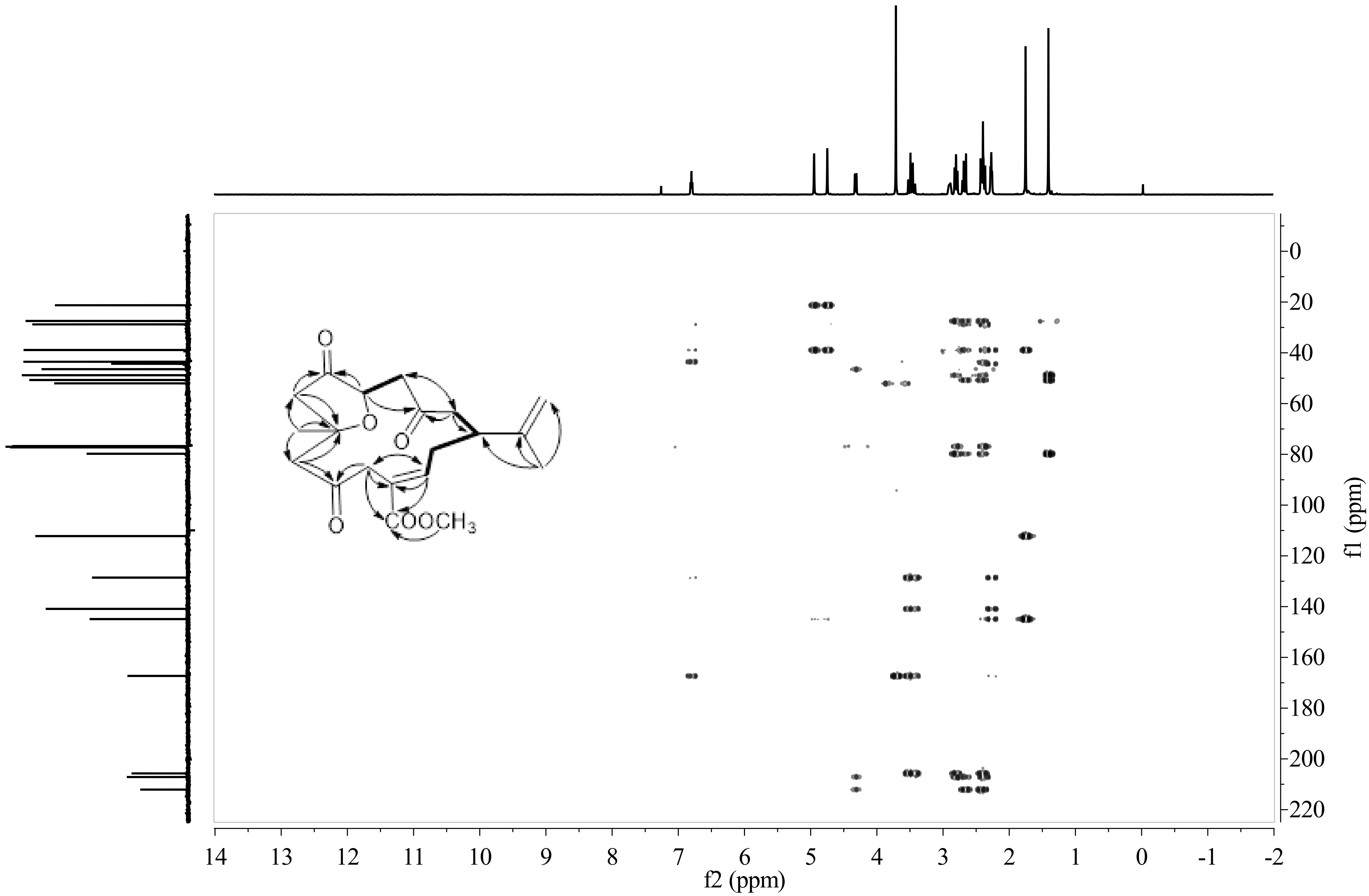

Figure SS47 HMBC spectrum of sinulin D (5)

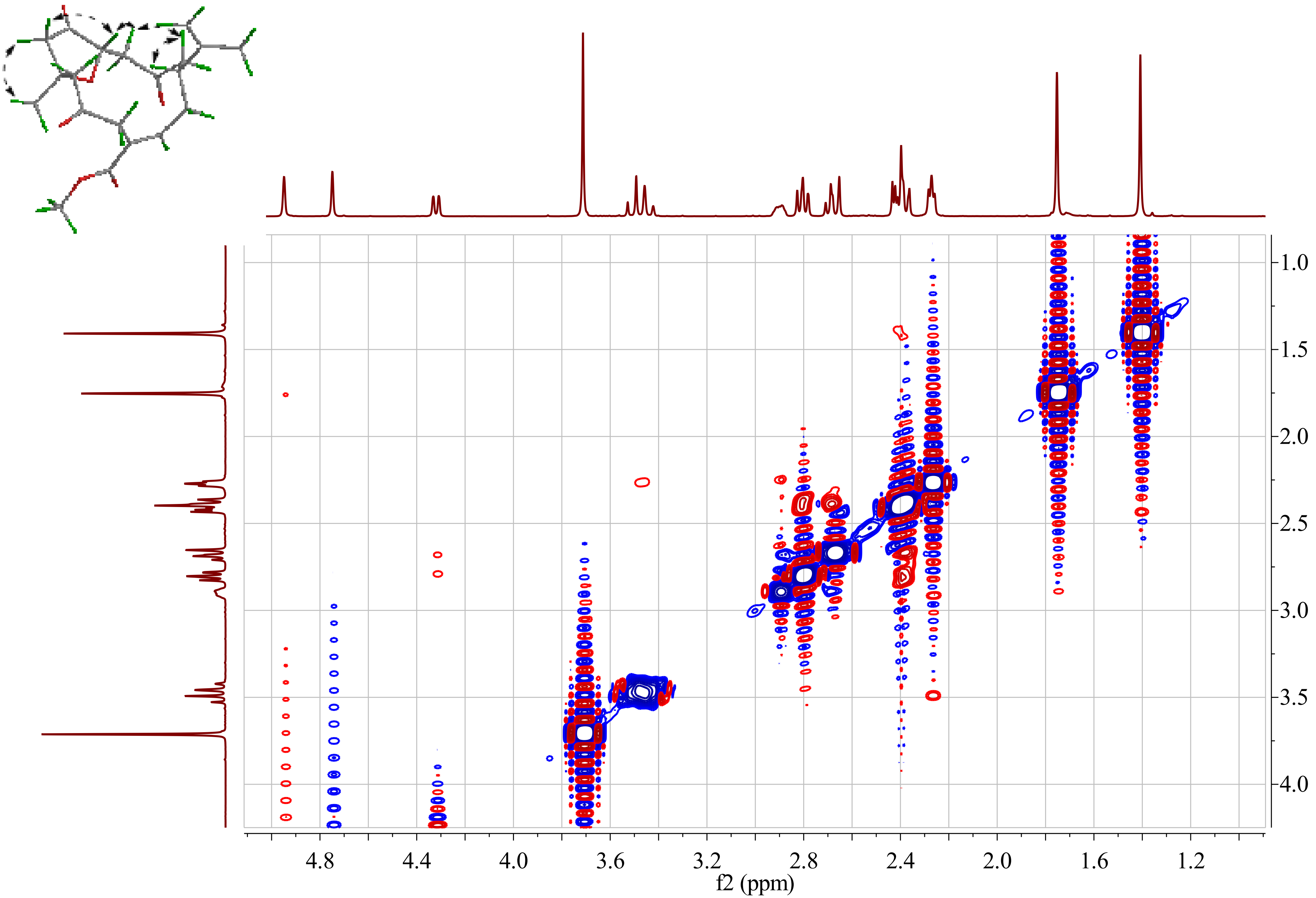

Figure SS48 NOESY spectrum of sinulin D (5)
